# Mapping socioeconomic factors driving antimicrobial resistance in humans: An umbrella review

**DOI:** 10.1016/j.onehlt.2025.100986

**Published:** 2025-02-10

**Authors:** Gunnar Ljungqvist, Robin van Kessel, Elias Mossialos, Victoria Saint, Jelena Schmidt, Alexander Mafi, Alison Shutt, Anuja Chatterjee, Esmita Charani, Michael Anderson

**Affiliations:** a LSE Health, Department of Health Policy, London School of Economics and Political Science, London, United Kingdom; b Department of International Health, Care and Public Health Research Institute (CAPHRI), Maastricht University, Maastricht, the Netherlands; c Institute of Global Health Innovation, Imperial College London, London, United Kingdom; d Department of Population Medicine and Health Services Research, School of Public Health, Bielefeld University, Germany; e British Medical Journal, London, United Kingdom; f Faculty of Medicine, Imperial College London, London, United Kingdom; g National Institute for Health and Care Excellence, London, United Kingdom; h Division of Infectious Diseases & HIV Medicine, Department of Medicine, Groote Schuur Hospital, University of Cape Town, South Africa; i Health Organisation, Policy, Economics (HOPE), Centre for Primary Care & Health Services Research, The University of Manchester, United Kingdom

**Keywords:** Antimicrobial resistance, Antibiotic use, Drug resistance, Socioeconomic factors, One health, Transmission

## Abstract

**Introduction:**

Antimicrobial resistance (AMR) is one of the biggest public health challenges of our time. National Action Plans have failed so far to effectively address socioeconomic drivers of AMR, including the animal and environmental health dimensions of One Health.

**Objective:**

To map what socioeconomic drivers of AMR exist in the literature with quantitative evidence.

**Methods:**

An umbrella review was undertaken across Medline, Embase, Global Health, and Cochrane Database of Systematic Reviews, supplemented by a grey literature search on Google Scholar. Review articles demonstrating a methodological search strategy for socioeconomic drivers of AMR were included. Two authors extracted drivers from each review article which were supported by quantitative evidence. Drivers were grouped thematically and summarised narratively across the following three layers of society: People & Public, System & Environment, and Institutions & Policies.

**Results:**

The search yielded 6300 articles after deduplication, with 23 review articles included. 27 individual thematic groups of drivers were identified. The People & Public dimensions contained the following themes: age, sex, ethnicity, migrant status, marginalisation, sexual behaviours, socioeconomic status, educational attainment, household composition, maternity, personal hygiene, lifestyle behaviours. System & Environment yielded the following themes: household transmission, healthcare occupation, urbanicity, day-care attendance, environmental hygiene, regional poverty, tourism, animal husbandry, food supply chain, water contamination, and climate. Institutions & Policies encompassed poor antibiotic quality, healthcare financing, healthcare governance, and national income. Many of these contained bidirectional quantitative evidence, hinting at conflicting pathways by which socioeconomic factors drive AMR.

**Conclusion:**

This umbrella review maps socioeconomic drivers of AMR with quantitative evidence, providing a macroscopic view of the complex pathways driving AMR. This will help direct future research and action on socioeconomic drivers of AMR.

## 1 Introduction

Antimicrobial resistance (AMR) has grown into a significant threat to the future of modern medicine, and one of the biggest public health challenges of our time [1]. It is estimated that 1.27 million people lost their lives in 2019 as a direct result of AMR, with projected estimates pushing this number to 10 million annually by 2050 [2]. Simultaneously, the world stands to lose approximately 3 trillion USD in GDP by 2050 because of AMR [3]. The cumulative volume and frequency of antibiotic use has steadily increased since 2000 [4,5], which has been observed over human, animal, and environmental sectors, and is responsible for ever increasing exposure of microorganisms to the selective pressure of antimicrobials. Simultaneously, antibiotic innovations have slowed down considerably [6,7], resulting in the increasingly concerning prospect of our current arsenal of antibiotics being rendered useless. Without any effective cure, previously treatable infections may result in significant and avoidable morbidity and mortality, compromising our ability to perform surgeries or provide immunosuppressive treatment [8].

The existing national action plans developed to date have failed to consider how socioeconomic and sociocultural drivers of health impact the emergence and spread of AMR, and antibiotic use [9]. This is despite growing evidence on interactions between social determinants of health and AMR. For example, adopting an intersectional lens is required to better understand how and why race, ethnicity, gender, education levels, and institutional power dynamics can impact infection related care, antibiotic access and use, and the spread of AMR [10]. Low- and middle-income countries (LMICs) are disproportionately affected by AMR due to an interplay between political, cultural, and infrastructural factors [11]. There is also growing attention on the animal and environmental drivers (i.e. the One Health drivers) of AMR. The use of antibiotics in animal farming and its dissemination into waterways is well-documented and has been highlighted as an area of concern in the WHO Global Action Plan for AMR [12]. This is supported by a growing body of evidence highlighting examples of animal spread and waterways contamination by AMR [13].

Understanding the full spectrum of socioeconomic factors that influence AMR is essential to inform research priorities and enable the development of comprehensive policies and strategies to manage its threat. Research on the socioeconomic drivers of AMR remains scattered, and at times conjectural. As such, this umbrella review aims to map what socioeconomic drivers of AMR in humans exist based upon a review of quantitative evidence and use this existing evidence to develop a conceptual framework to guide policy and practice.

## 2 Methods

### 2.1 Umbrella review methodology

We performed an umbrella review with thematic synthesis using methodological guidelines developed by the Joanna Briggs Institute Manual for Evidence Synthesis [14]. An umbrella review is a synthesis of existing systematic reviews and meta-analyses on a specific topic, intended for capturing a wide base of evidence on a large topic through a smaller number of review articles. We focused specifically on socioeconomic drivers of AMR with quantitative evidence for several reasons. First, previous reviews have focused on qualitative evidence on the drivers of AMR [15], and to date there has been no umbrella review focused specifically on socioeconomic drivers of AMR with quantitative evidence. Second, a mapping exercise of socioeconomic drivers of AMR with quantitative evidence can help inform the development of modelling exercises that examine transmission dynamics of resistant microorganisms, and the economic cost of resistant infections. Third, it is hoped this summary of socioeconomic drivers of AMR with quantitative evidence could be used alongside pre-existing summaries of qualitative evidence to leverage support from policymakers to implement and strengthen inter-sectoral approaches to tackling AMR as both types of evidence resonate with policymakers [16]. We reported the findings according to the Preferred Reporting Items for Overviews of Reviews (PRIOR) guidelines (Supplementary Table 1) [17]. Given the scoping nature of this review, no review protocol was published.

### 2.2 Eligibility criteria

Reviews were included based on the following criteria: (1) Any review where an explicit methodological search strategy was employed including systematic reviews, rapid reviews, umbrella reviews, scoping reviews; (2) reviews should explicitly report on AMR or an AMR behaviour (defined below); (3) the directional relationship of the socioeconomic factor to AMR was allowed to remain implicit and broad in order to maximise capture; (4) reviews needed to report quantitative data relating to drivers of AMR outlined either within the body of the article or within the supplementary material; (5) the main focus of the review needed to include socioeconomic drivers of AMR in humans, although this could include the human-animal interface, or the human-environment interface; and (6) studies should be written in English and published on or after the 1st January 2010. This timeframe was chosen because of feasibility constraints but significant evidence was summarised before this date as captured reviews included longer timeframes within their inclusion criterion. Reviews were excluded if they summarised quantitative evidence narratively or did not include specific point estimates of quantitative studies in either the manuscript or supplementary material. Furthermore, reviews focusing on AMR interventions were excluded, including reviews on antimicrobial stewardship.

Drivers are conceptualised as having a direct association with either a metric of AMR (such as aggregate indices, prevalence rates, resistance rates) or behaviours with a well-established causality (such as antibiotic use, or self-medication with antibiotics (SMA)). Evidence on drivers of AMR in animal and environmental health settings were captured if studies estimated the relationship between these drivers and the increased risk of AMR in humans. We did not disaggregate our findings related to AMR into individual strains of resistant pathogens.

### 2.3 Search strategy

We systematically searched four scientific databases: MEDLINE (OVID), Embase (OVID), Global Health, and the Cochrane Database of Systematic Reviews. These databases were chosen for their health-specific nature that covers literature from high-, middle-, and low-income countries. The scientific search was supplemented with a non-systematic search for grey literature using Google Scholar (first 200 hits) [18]. The full query for the scientific databases is shown in Supplementary Table 2. An information specialist at the London School of Economics and Political Science Library further validated the search strategy.

The search strategy was executed on 14 March 2023. The complete screening process was performed by 1 reviewer (GL). A second reviewer (RVK) screened a subset of the articles (1246/6300, 19.78 %) to improve the methodological robustness of the literature review and interrater reliability scores were computed. Any disagreements between the reviewers were resolved by an independent third reviewer (MA). The interrater agreement between the 2 reviewers was calculated using Cohen κ in R (version 4.1.2). Deduplication was performed using Endnote (version 22), and screening was performed using Covidence.

### 2.4 Data extraction and analysis

Two reviewers (GL, JS) identified key parameters, such as years reviewed and geographic scope, from each included article. Each review was appraised and scored for quality, in accordance with an adapted CASP (Critical Appraisal Skills Programme) tool [19], and was allocated a ranking of low, moderate, or high quality based on the average of the two scores. This exercise was purely an appraisal tool and was not used to exclude any reviews. Each review was then independently searched to extract any socioeconomic factor with a quantitative relationship with our AMR indicators. The reviewers extracted any relevant odds ratios or other quantitative data for each socioeconomic driver and their relationship with AMR for each review. Subsequently, a deductive thematic analysis was conducted, which captured the breadth of socioeconomic factors identified in the literature. A third reviewer (MA) addressed any discrepancies between socioeconomic drivers identified. An emphasis was placed on capturing as many different themes as possible during the thematic analysis, at the expense of depth of detail.

Socioeconomic drivers were extracted along with their quantitative metric, and then further subcategorised into 3 levels: People & Public, System & Environment, and Institutions & Policies. These levels were decided collaboratively among co-authors (GL, RVK, MA, EM) a priori based on established literature on the social determinants of infectious diseases. The selected framework, developed by Toro-Alzate et al. [20] was chosen over other existing frameworks for the following reasons: contemporaneous to our study; intuitive to understand; grounded in the social sciences; sufficiently flexible to adapt to our research question; and designed for a similar objective, which was to investigate the socioeconomic determinants of AMR. The “People & Public” dimension focused on the microscopic social dimensions, including individuals themselves as well as parameters directly influencing their individual lived behaviour. “Systems & Environment” is intended to capture community-wide parameters affecting individuals' lives. This includes social, logistical, and even natural parameters existing around households. This can be conceptualised as factors dictating the ways in which individuals interact with their environment. “Institutions & Policies” approaches AMR from a broader political perspective by focusing on the rules and regulations which, while removed from the individuals, affect the parameters which in turn impact the individuals. This conceptually includes the political machinery, laws, and regulations, and the wider scientific community. Once the socioeconomic factors were identified, they were consolidated through iterative discussion between the two reviewers to avoid duplication or overlap between drivers. This methodological approach has been validated in previous research [21]. Thematic analysis was performed in Atlas.TI version 23. We chose not to collate any quantitative findings for each socioeconomic driver across identified reviews, as the primary focus on this review was a mapping exercise to identify which socioeconomic factors are quantitatively related to AMR. Meta-analysis or pooling of quantitative evidence on the relative importance of specific quantitative socioeconomic drivers of AMR should be the subject of future research with more targeted reviews that have a narrower focus.

## 3 Results

### 3.1 Overview of search results

**Fig. 1:**
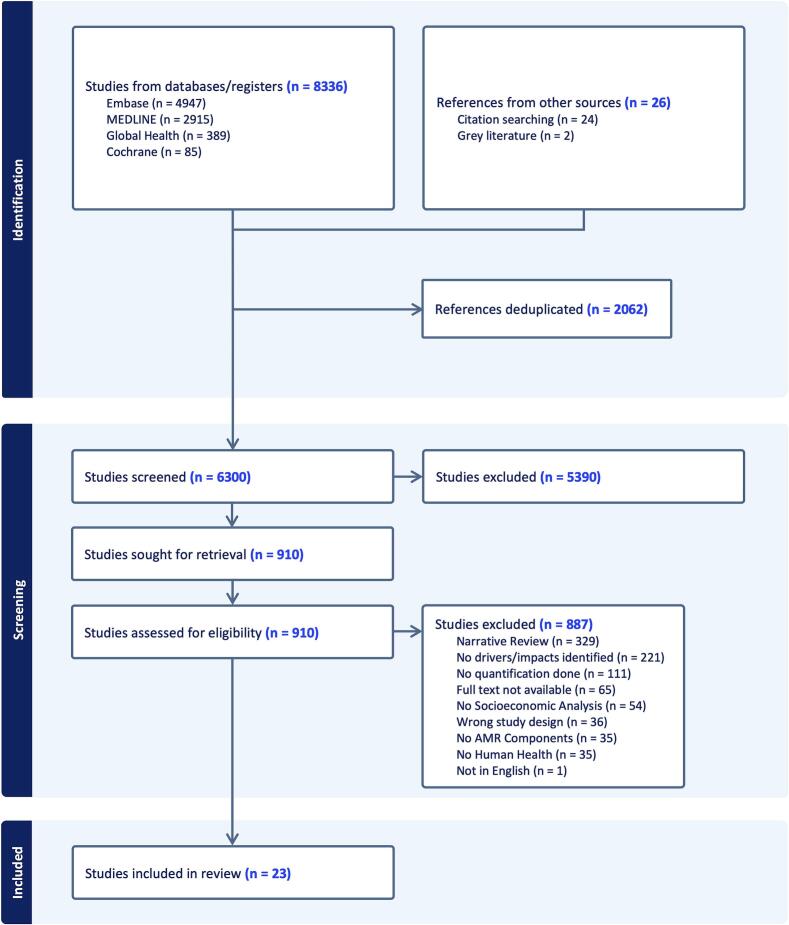
PRISMA (Preferred Reporting Items for Systematic Reviews and Meta-Analyses) flowchart outlining the data collection process.

The search strategy (Fig. 1) yielded a total of 8362 records from academic database searches (6300, 75.34 % after deduplication). This included 26 references from manual citation searching and a grey literature search. Ultimately, we included 23 articles in this review.

The publication years ranged from 2017 to 2022, with 52 % being published on or after 2021. The cumulative number of references captured by these reviews totaled to 2048 articles, not accounting for overlaps. In terms of geographic coverage, 18 of the reviews covered the Western Pacific Region [15,[22], [23], [24], [25], [26], [27], [28], [29], [30], [31], [32], [33], [34], [35], [36], [37], [38]], 14 covered the South-East Asian Region [15,22,23,27,28,[30], [31], [32], [33], [34], [35], [36], [37], [38]], 12 covered the Region of the Americas [15,[30], [31], [32], [33], [34], [35], [36], [37], [38], [39], [40]], 12 covered the European Region [15,[30], [31], [32], [33], [34], [35], [36], [37], [38],^41^,^42^], 12 covered the Eastern Mediterranean Region [15,22,23,[30], [31], [32], [33], [34], [35], [36], [37], [38]], and 11 covered the African Region [15,[30], [31], [32], [33], [34], [35], [36], [37], [38],^43^]. In terms of One Health coverage, 7 articles included the human-animal interface [15,24,28,29,[34], [35], [36]] and 6 articles covered human-environment interface [24,28,30,34,36,43], with 4 of these covering both human-animal and human-environment interfaces [24,28,34,36]. In the subset screening of the total sample by the second reviewer, we found a crude interrater agreement score of 82.83 % (1032/1246 observations) between the 2 reviewers. We also accounted for the possibility of reaching interrater agreement by chance by computing Cohen κ (0.352), which indicated a fair agreement between the observers. In terms of quality, 4 reviews scored low, 12 moderate, and 7 high quality. An extraction sheet of article characteristics and quality scores are included in Supplementary Tables 3 and 4. The full list of excluded articles at full-text screening with reason for exclusion is presented in Supplementary Table 5. The extracted socioeconomic drivers are summarised in Supplementary Table 6.

### 3.2 Drivers of AMR

#### 3.2.1 People and public

A wealth of data was found describing intrinsic core demographic factors, such as age, gender, ethnicity, or sexual behaviour. However, the directionality of the association was often difficult to establish. Sun et al. [15] found evidence that being male may be both a risk factor [44] and a protective factor [45] for antibiotic use: the former demonstrated by higher rates of SMA among males in Mozambique; the latter by male students with a lower tendency to store antibiotics at home. Age was similarly multidirectional in nature. For instance, older age (>65) was found in Portugal to be a risk factor for contracting resistant *Enterobacteriaceae* [34,46], whilst a study in Germany found older participants to be less likely to SMA [15,47]. Childhood was, in contrast, widely found to be positively correlated with AMR, both through drug-resistant infection prevalence rates, and SMA behaviour [15,23,24,29,32,34]. The correlation between ethnicity and AMR was sparse and contradictory, with some evidence that being African-American [34,39,[48], [49], [50]] or First Nations compared to Caucasian [40,51,52], but also that being Caucasian compared to non-Caucasian [34,53] were all potential risk factors for contracting Methicillin-resistant *Staphylococcus aureus* (MRSA). In contrast, there was more consistent evidence supporting a correlation between migration status and AMR, with recent immigration or forcible displacement being consistently established as a risk factor for SMA, antibiotic storage, and drug-resistant organism carriage or infection [15,25,42]. This was not clear cut however, as evidence from Hong Kong suggested immigrants were also less likely to SMA compared to local-born persons [15,54]. Other factors of societal marginalisation, such as intravenous drug use [30,34,[55], [56], [57], [58], [59]], present or previous homelessness [30,34,48,55,56], and prison [34,55,56] were also cited as risk factors for carrying MRSA. Men who have sex with men (MSM) were also found to be at higher odds of drug-resistant infections compared to non-MSM, although this was limited to HIV or Sexually Transmitted Disease coinfections [34,60,61].

Evidence for a relationship between AMR and educational status or socioeconomic status (SES) were mixed. Parents with a higher educational level were identified as being at higher risk of risky behaviours such as self-medicating their children prophylactically or pressurising doctors for prescriptions in various studies in China [15,32,34,62,63]. Other studies stated that higher parental educational attainment was protective against these very same behaviours [15,32,64]. Having a university-level education for instance, was identified in Jordan as being protective against self-medicating their children [34]. Higher income was similarly described as either increasing the risk of antibiotic use [15,65], or decreasing it, depending on the study [15,62]; although having a lower income and being of lower SES was unilaterally identified as a risk factor for SMA and contracting AMR infections [30,32,34]. Household composition was widely investigated, with evidence suggesting a significant correlation between overcrowding and AMR. This was described in situations with any number of children in the household causing an increased risk of contracting a drug-resistant infection [29], multiple-children households being at higher risk of SMA [15,25,32], and families with older children [25] as well as families with more than 5 family members of any age [30,36] in the household being at higher risks of contracting drug-resistant infections. Sharing space with people with known AMR was understandably also correlated with contracting drug-resistant infections [36]. Giving birth, mother-to-child contact, and breastfeeding were identified as risk factors for AMR transmission [36], although conversely instances were also found of breastfeeding protecting against MRSA transmission [23,29,36]. Personal hygiene such as daily showering and antibacterial hand soap use was found to be overall protective against drug-resistant organism transmission [36]. The sharing of hygiene equipment such as washcloths and ointments, as well as children wearing nappies, were possibly associated with carrying and transmitting drug-resistant organisms [34,36]. Finally, individual lifestyle behaviours such contact sport [36], sauna use [34], and not using condoms [34] were also positively correlated to carrying drug-resistant organisms. Evidence on smoking was somewhat more mixed: smoking appeared protective against MRSA carriage in adults [29], however being a heavy smoker was associated with a higher risk of infection by levofloxacin-resistant pneumococci [34].

#### 3.2.2 System and environment

Occupation was frequently reported in association with AMR, specifically healthcare and animal-related occupations. Multiple sources reported healthcare occupations as a risk factor not only for the individuals [15,25,36], but also for their families [15,25,29,36], through household transmission and behaviours such as SMA or antibiotic storage. Working in the farming industry was reported as a risk factor for developing AMR, with contact with cattle, swine, poultry, mink, horses, goats, and hogs all exhibiting significant correlation with the transmission of drug-resistant infections [29,34,36]. In contrast, the evidence on household pets such as cats and dogs was more mixed [34,36], with veterinarians facing higher risks of drug-resistant infections than the general population, while pet owners seem to face lower risks of transmission than non-pet owners. While various aspects of farm working were associated with drug-resistant organism transmission, including manure, pigsties, and regularly visiting farms, there was also evidence that working on smaller farms was protective when compared to bigger farms [36]. Working in the food supply chain was heavily associated with high rates of drug-resistant infections, with factors such as working in a slaughterhouse, handling raw meat, working in food distribution, and giving animals antibiotics all identified as factors associated with higher rates [36]. In certain contexts, eating pork, raw milk, and dried poultry was found to be associated with higher drug-resistant- organism carriage, further underlining the role of the food supply chain as a mode of transmission [36]. Sharing water sources with livestock was also identified as a risk factor for drug-resistant infections [36].

The evidence on urbanicity was mixed, with a multitude of research highlighting both urban [15,26,29,34] and rural areas [15,[24], [25], [26],^32^,^34^] as risk factors for drug-resistant infections. Prevalence studies (e.g. AMR was more prevalent in urban areas such as Northern Taiwan [29,34]) and behavioural surveys (SMA was more likely in rural Tanzania [32]) add detail to this picture, suggesting that while AMR might be more prevalent in denser urban settings, antibiotic usage and SMA might be more widespread further away from healthcare facilities. Children's day-care was also highlighted as a risk factor for drug-resistant infections [23,29,34]. Independently of population density, subregional income per capita was identified as a risk factor, with deprived neighbourhoods in the UK exhibiting a positive correlation with antibiotic resistant *E. coli* prevalence [30]. Tropical areas were also found to have higher rates of carbapenem-resistance when compared to non-tropical countries [22], although disentangling this from other macroeconomic parameters may be challenging. Recreational travel was consistently a risk factor for transmission and carriage of drug-resistant organisms, although the heaviest evidence related to travel to tropical regions within Asia [33,34,36], Africa [34,36], and Latin America [33,34,36]. Furthermore, travel behaviours including eating food with locals [36], mass pilgrimages [34], and using healthcare [33,36,37] were all risk factors for drug-resistant organism carriage.

#### 3.2.3 Institutions and policies

The use of expired antibiotics was found to be associated with higher rates of resistant strains, particularly in LMICs [35,66]. Specific contributing factors included the fiscal practices of HICs exporting near-expired drugs to LMICs, inadequate quality control of transport and storage conditions, and the increased degradation caused by light, heat, and humidity in tropical climates [35,67]. Healthcare financing was also related to AMR, with increases in out-of-pocket expenditures associated with an increase in prevalence of drug-resistant strains [34]. The relation that health insurance status had was only demonstrated in children, where children with health insurance were at higher risks of SMA, whereas adults seemed at lower risk of SMA with health insurance [15,25,32]. Healthcare governance was also found to be correlated with inappropriate antibiotic use: antibiotic dispensing increased significantly in the absence of licensed pharmacists; in village clinics compared to bigger clinics; and with private primary care practitioners compared to public [25].

At the macroeconomic scale, associations were found between lower national income and higher rates of AMR. This was found with resistance rates [22,43] with the notable exception of MRSA infections. While MRSA is positively associated with gross national income, the proportion of methicillin resistance among *Staphylococcus aureus* infections was inversely correlated with gross national income [22,27]. There has also been an association between lower national income and behavioural parameters such as increased SMA [15].

### 3.3 Mapping of socioeconomic drivers of AMR in humans

**Fig. 2:**
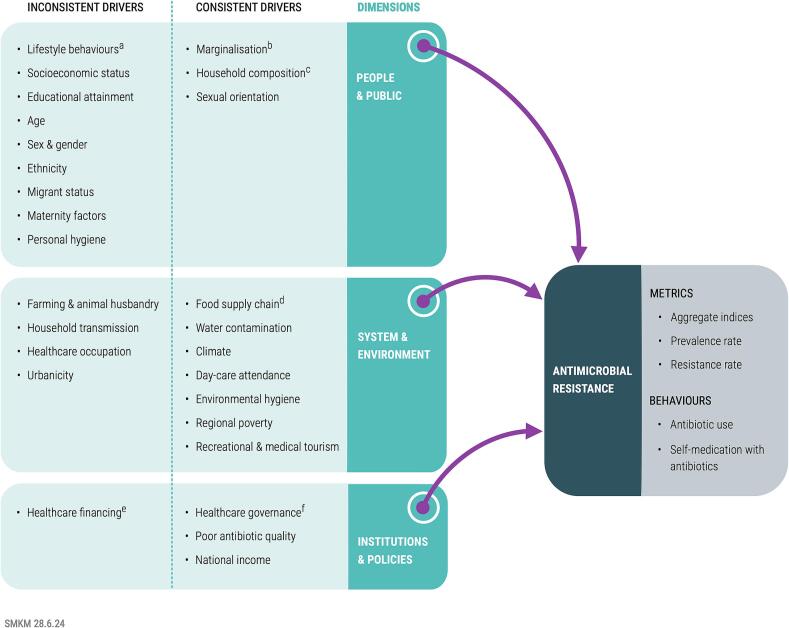
Mapping of Socioeconomic Drivers of AMR: Drivers were divided according to three distinct socioeconomic dimensions: People & Public, System & Environment, and Institutions & Policies. Drivers were further subdivided according to “consistency” of the evidence: “consistent” drivers exhibited homogenous data driving AMR or antibiotic use; “inconsistent” drivers showed more bidirectional data, suggesting at times a causative, and at other times a preventative relation to AMR and antibiotic use. The type of data these drivers influenced is outlined under the “Antimicrobial Resistance” box, and can be summarised as either metrics of resistance, or behaviours. a: eg smoking, sauna use, contact sports; b: eg intravenous drug use, homelessness, incarceration; c: eg no children, multiple children, grandparents as caregivers; d: eg slaughterhouse worker, foodhandler, raw milk drinking; e: eg child health insurance, out-of-pocket financing; f: eg private sector vs public sector primary care, pharmacists being licensed.

From the 23 included documents, we extracted 27 groups of AMR drivers. While there were variations in strength, direction, and consistency of correlation, all demonstrated some association with AMR metrics (such as resistance rates or prevalence data) or behaviours with a well-established causality (such as antibiotic use or SMA). These factors and have been collated into a conceptual framework in Fig. 2. Socioeconomic drivers of AMR were classified as either “consistent” drivers which had a unidirectional relationship with an AMR indicator, or “inconsistent” drivers which had bidirectional or mixed relationships with AMR indicators. The detailed drivers in each theme and relevant quantitative evidence are contained in supplementary material Supplementary Table 7.

## 4 Discussion

Our review provides an overview of the main socioeconomic factors driving AMR, and demonstrates an abundance of data points in the existing literature to pull from. While not an exhaustive effort, our findings deliver quantitative evidence echoing research in the wider literature, highlighting remoteness [68], migration and travel [69], GDP [70], regional poverty [71], age [72], and various social constructs such as gender and race [10]. Similarly, our findings are congruent with sector-specific efforts which describe risks of AMR transmission through food supply chain workers [73], the animal-human interface [74], and through water contamination [75].

There were notable gaps in the data elicited by our review, with factors otherwise understood to be associated with AMR in the broader scientific literature. Gender, for instance, is a widely discussed driver of AMR through shaping of health-seeking behaviours, traditionally gendered occupations such as healthcare or animal husbandry, and physiological risks associated with biological sex [10,[76], [77], [78]]. While our research did highlight gender as a frequently addressed socioeconomic driver, the data focused heavily on health-seeking behaviours, and failed to capture the breadth of the intersectionality of gender as it pertains to other AMR risks (i.e. occupation, education level, ethnicity) and exposure. Climate change is frequently discussed in the context of AMR, with growing evidence of rising temperatures increasing the survivability and transmissibility of resistant organisms, and climate-induced migratory changes generating new global transmission routes [70,[79], [80], [81], [82]]. While our project did uncover evidence on climate and its correlation to AMR [22], there were no quantitative data points elicited in relation to climate change. Corruption in both healthcare administration and broader governmental administration, has been linked with AMR rates in the scientific literature. This was the case even when measuring for subjective measures such as the perceived presence of corruption within a system [[83], [84], [85]]. Pollution is also described in the literature as a driver of AMR, through antibiotic waste contamination of waterways or airborne particulate matter carrying antibiotic resistance elements [79,[86], [87], [88]]. This driver was entirely absent from our study. Conflict and geopolitical instability have been suggested as potentially driving AMR, evidenced by prevalence studies in areas of conflict. Mechanisms such as disruption of routine or preventative healthcare, displacement of populations, and higher risk of surgical wound infections due to traumatic injuries, have all been posited as mechanisms for this association [[89], [90], [91], [92]]. The evidence is sparse however on this point, explaining its absence from our findings. Finally, while we discussed national income in our findings, little quantitative evidence was found on per capita expenditure on health, despite this being discussed in the wider literature [70,93]. Future research efforts targeted at these drivers would be beneficial in understanding the relative impact these drivers have on the AMR crisis.

The quantitative data highlighted in our paper was highly variable in external validity and in the direction of association, with certain factors being described as both causative and protective with relation to AMR. This phenomenon can be explained due to the two distinct driving forces of AMR described by Collignon: the volume of antimicrobial used, and contagion [94]. Socioeconomic factors can thus be analyzed through either of these two lenses. Factors driving the consumption of antimicrobials lead to AMR through driving mutation of resistance genes under selective pressure. Any factor resulting in an increase in antimicrobial consumption, whether clinically indicated or not, would fall in this category. It is important to note here that incorrect or insufficient antibiotic usage when otherwise clinically indicated may also drive resistances, thus it would be inaccurate to infer a simple volume-dependent relation between antimicrobial usage and AMR emergence. Our review highlighted multiple factors at the “People & Public” level which were found to increase the risk of SMA, including immigration [15,25], lower socioeconomic status [15,32], and younger and older age [15,24].

Conversely, factors driving contagion lead to higher rates of AMR through the spread of resistant organisms or genes from human-to-human, or between human, animal, and environmental reservoirs. This has been evidenced in droplet and fomite transmission on a human-to-human basis, but also in animal contact, agriculture, food supply chain, and waterways [95]. Many of the factors described at the “System & Environment” level of our mapping exercise fall into this category, including urbanicity, household transmission, and environmental hygiene.

Dividing the socioeconomic drivers of AMR into these two distinct types is helpful in understanding how AMR is driven forward. However, many of these socioeconomic factors interact with both forces simultaneously and separately. Persons living in urban areas may for instance enjoy higher access to healthcare and thus antibiotics than their rural counterparts [96], but may also be subjected to higher population density and thus higher risk of contagion [97]. The independent relationship that each of these two forces has with each socioeconomic factor helps explain some of the complexities and incongruences noted in the data summarised in this review.

While the AMR crisis has garnered increasing attention in recent years, many National Action Plans fail to address the importance of socioeconomic factors in generating AMR [9]. This research provides quantitative data on socioeconomic drivers of AMR, and may thus be of interest to policymakers working on integrating the socioeconomic dimension into AMR policies. This paper illustrates the kind of data which exists, providing examples of how one might measure the association between socioeconomic and sociocultural factors and AMR within a local context. This could also translate into mechanisms to measure the impact of future policies addressing the socioeconomic determinants of AMR.

### 4.1 Strengths and limitations

To our knowledge this is the first paper that maps socioeconomic drivers of AMR with quantitative evidence from reviews using a One Health perspective. The strength of this review lies in the broad scope of the search strategy. By aiming for an exhaustive list of search terms, this review was able to pull through a large volume of papers from a variety of geographies and One Health dimensions. This ensured a broad scope to inform the thematic analysis with a wealth of different perspectives not limited to the human biomedical model. This is evidenced by the multitude of identified themes relating to societal structures and systems, animal health, environmental health, and cross-reservoir transmission. Furthermore, in grounding itself in recognised frameworks, this conceptual model builds upon established literature to provide a concise and peer-reviewed overview of the current state of knowledge.

Limitations of this review need to be considered. First, the findings should be interpreted as scoping, meaning it provides a high-level overview of the literature and may not capture more intricate and field-specific factors. Second, we acknowledge that this review only captures the drivers that are quantified within the current AMR literature, thus missing out on the body of qualitative evidence on this topic or theorised driving factors. Thirdly, we did not attempt to quantify the relative contribution of different drivers to increased AMR as the objective of this review was to map these drivers. A comparative analysis of each factor such as a meta-analysis was considered beyond the scope of this project, but would have allowed a more nuanced conversation on each identified socioeconomic driver. This could be the focus of future research. Finally, we acknowledge that this review makes broad conclusions about the drivers and impacts of AMR holistically and may not be applicable to individual situations.

## 5 Conclusion

This review illustrated a breadth of quantitative data from human, animal, and environmental sectors, providing more contextualised information for national policymakers to understand the ways in which policy levers may influence AMR in their setting. We highlight the complex interconnectivity between socioeconomic factors operating across these sectors, and thus the need to move away from siloed committees and towards a multisectoral approach to tackling the AMR crisis at a national and international level, fostering a sense of shared purpose and shared accountability across disciplines and sectors.

## Funding

Funding for the development of this document was kindly provided by the World Economic Forum, which was supported by the 10.13039/501100009708Novo Nordisk Foundation.

## CRediT authorship contribution statement

**Gunnar Ljungqvist:** Writing – review & editing, Writing – original draft, Methodology, Investigation, Formal analysis, Data curation, Conceptualization. **Robin van Kessel:** Writing – review & editing, Validation, Project administration, Methodology, Investigation, Formal analysis, Data curation, Conceptualization. **Elias Mossialos:** Writing – review & editing, Supervision, Project administration, Funding acquisition, Conceptualization. **Victoria Saint:** Writing – review & editing, Validation, Conceptualization. **Jelena Schmidt:** Writing – review & editing, Methodology, Data curation. **Alexander Mafi:** Writing – review & editing, Methodology, Investigation. **Alison Shutt:** Writing – review & editing, Methodology, Conceptualization. **Anuja Chatterjee:** Writing – review & editing, Methodology, Investigation, Data curation, Conceptualization. **Esmita Charani:** Writing – review & editing, Validation, Conceptualization. **Michael Anderson:** Writing – review & editing, Validation, Supervision, Project administration, Methodology, Investigation, Formal analysis, Data curation, Conceptualization.

## Declaration of competing interest

The authors declare the following financial interests/personal relationships which may be considered as potential competing interests.

Elias Mossialos reports financial support was provided by World Economic Forum. Victoria Saint reports a relationship with World Health Organization that includes: consulting or advisory. Victoria Saint reports a relationship with German Alliance for Global Health Research that includes: funding grants. Victoria Saint reports a relationship with ERASMUS Programme that includes: travel reimbursement. Alison Shutt reports a relationship with National Institute for Health Research that includes: funding grants. Esmita Charani reports a relationship with Pfizer that includes: speaking and lecture fees. Esmita Charani reports a relationship with bioMérieux Inc. that includes: speaking and lecture fees. Esmita Charani reports a relationship with World Health Organization that includes: funding grants. Esmita Charani reports a relationship with Wellcome Trust that includes: funding grants. Michael Anderson reports a relationship with World Health Organization Regional Office for Europe that includes: consulting or advisory. If there are other authors, they declare that they have no known competing financial interests or personal relationships that could have appeared to influence the work reported in this paper.

## Data Availability

No data was used for the research described in the article.

## Appendix A Supplementary data

Supplementary material: PRIOR checklist; Overview of the search string; Descriptive table of included articles; Quality assessment of reviewed articles; List of excluded articles at full-text review; Detailed list of drivers; Full quantitative data extraction table Image 1

## References

[1] Gerhard D. (2023). https://www.the-scientist.com/news-opinion/antimicrobial-resistance-the-silent-pandemic-71196.

[2] Antimicrobial Resistance Collaborators (2022).

[3] Taylor J., Hafner M., Yerushalmi E., Smith R., Bellasio J., Vardavas R., Binkowska-Gibbs T., Rubin J. (2014).

[4] Browne A.J., Chipeta M.G., Haines-Woodhouse G., Kumaran E.P.A., Hamadani B.H.K., Zaraa S., Henry N.J., Deshpande A., Reiner R.C., Day N.P.J., Lopez A.D., Dunachie S., Moore C.E., Stergachis A., Hay S.I., Dolecek C. (2021). Global antibiotic consumption and usage in humans, 2000–18: a spatial modelling study. The Lancet Planet. Health.

[5] T.P. Van Boeckel, S. Gandra, A. Ashok, Q. Caudron, B.T. Grenfell, S.A. Levin, R. Laxminarayan, Global antibiotic consumption 2000 to 2010: an analysis of national pharmaceutical sales data, Lancet Infect. Dis. 14 (2014) 742–750. doi:10.1016/S1473-3099(14)70780-7. 25022435

[6] Anderson M., Panteli D., van Kessel R., Ljungqvist G., Colombo F., Mossialos E. (2023). Challenges and opportunities for incentivising antibiotic research and development in Europe. The Lancet Regional Health - Europe.

[7] Shafiq N., Pandey A.K., Malhotra S., Holmes A., Mendelson M., Malpani R., Balasegaram M., Charani E. (2021). Shortage of essential antimicrobials: a major challenge to global health security. BMJ Glob. Health.

[8] Anderson M., Cecchini M., Mossialos E. (2020).

[9] Charani E., Mendelson M., Pallett S.J.C., Ahmad R., Mpundu M., Mbamalu O., Bonaconsa C., Nampoothiri V., Singh S., Peiffer-Smadja N., Anton-Vazquez V., Moore L.S.P., Schouten J., Kostyanev T., Vlahovic-Palcevski V., Kofteridis D., Correa J.S., Holmes A.H. (2023). An analysis of existing national action plans for antimicrobial resistance-gaps and opportunities in strategies optimising antibiotic use in human populations. Lancet Glob. Health.

[10] Charani E., Mendelson M., Ashiru-Oredope D., Hutchinson E., Kaur M., McKee M., Mpundu M., Price J.R., Shafiq N., Holmes A. (2021). Navigating sociocultural disparities in relation to infection and antibiotic resistance-the need for an intersectional approach. JAC-Antimicr. Res..

[11] Sulis G., Sayood S., Gandra S. (2022). Antimicrobial resistance in low- and middle-income countries: current status and future directions. Expert Rev. Anti-Infect. Ther..

[12] World Health Organization (2015).

[13] White A., Hughes J.M. (2019). Critical importance of a one health approach to antimicrobial resistance. EcoHealth.

[14] Aromataris E., Fernandez R., Godfrey C., Holly C., Khalil H., Tungpunkom P. (2020). https://jbi-global-wiki.refined.site/space/MANUAL/355829653/9.+Umbrella+reviews.

[15] Sun R., Yao T., Zhou X., Harbarth S., Lin L. (2022). Non-biomedical factors affecting antibiotic use in the community: a mixed-methods systematic review and meta-analysis. Clin. Microbiol. Infect..

[16] Mays N., Pope C., Popay J. (2005). Systematically reviewing qualitative and quantitative evidence to inform management and policy-making in the health field. J. Health Serv. Res. Policy.

[17] Gates M., Gates A., Pieper D., Fernandes R.M., Tricco A.C., Moher D., Brennan S.E., Li T., Pollock M., Lunny C., Sepúlveda D., McKenzie J.E., Scott S.D., Robinson K.A., Matthias K., Bougioukas K.I., Fusar-Poli P., Whiting P., Moss S.J., Hartling L. (2022). Reporting guideline for overviews of reviews of healthcare interventions: development of the PRIOR statement. BMJ.

[18] Haddaway N.R., Collins A.M., Coughlin D., Kirk S. (2015). The role of Google scholar in evidence reviews and its applicability to Grey literature searching. PLoS One.

[19] Spittlehouse C., Acton M., Enock K. (2000). Introducing critical appraisal skills training in UK social services: another link between health and social care?. J. Interprof. Care.

[20] Toro-Alzate L., Hofstraat K., de Vries D.H. (2021). The pandemic beyond the pandemic: a scoping review on the social relationships between COVID-19 and antimicrobial resistance. Int. J. Environ. Res. Public Health.

[21] van Kessel R., Roman-Urrestarazu A., Anderson M., Kyriopoulos I., Field S., Monti G., Reed S.D., Pavlova M., Wharton G., Mossialos E. (2023). Mapping factors that affect the uptake of digital therapeutics within health systems: scoping review. J. Med. Internet Res..

[22] Bonell A., Azarrafiy R., Huong V.T.L., Le Viet T., Phu V.D., Dat V.Q., Wertheim H., Van Doorn H.R., Lewycka S., Nadjm B. (2019). A systematic review and Meta-analysis of ventilator-associated pneumonia in adults in Asia: an analysis of National Income Level on incidence and etiology. Clin. Infect. Dis..

[23] Chan Y.Q., Chen K., Chua G.T., Wu P., Tung K.T.S., Tsang H.W., Lung D., Ip P., Chui C.S.L. (2022). Risk factors for carriage of antimicrobial-resistant bacteria in community dwelling-children in the Asia-Pacific region: a systematic review and meta-analysis. JAC-Antimicr. Res..

[24] Chansamouth V., Mayxay M., Dance D.A.B., Roberts T., Phetsouvanh R., Vannachone B., Vongsouvath M., Davong V., Inthavong P., Khounsy S., Keohavong B., Keoluangkhot V., Choumlivong K., Day N.P.J., Turner P., Ashley E.A., van Doorn H.R., Newton P.N. (2021). Antimicrobial use and resistance data in human and animal sectors in the Lao PDR: evidence to inform policy. BMJ Glob. Health.

[25] Coope C., Schneider A., Zhang T., Kadetz P., Feng R., Lambert H., Wang DeBin I., Oliver S., Michie C. Cabral (2022). Identifying key influences on antibiotic use in China: a systematic scoping review and narrative synthesis. BMJ Open.

[26] Duan L., Liu C., Wang D. (2021). The general population’s inappropriate behaviors and misunderstanding of antibiotic use in China: a systematic review and meta-analysis. Antibiotics.

[27] Lim W.W., Wu P., Bond H.S., Wong J.Y., Ni K., Seto W.H., Jit M., Cowling B.J. (2019). Determinants of methicillin-resistant Staphylococcus aureus (MRSA) prevalence in the Asia-Pacific region: a systematic review and meta-analysis. J. Global Antimicrob. Resist..

[28] Pham-Duc P., Sriparamananthan K. (2021). Exploring gender differences in knowledge and practices related to antibiotic use in Southeast Asia: a scoping review. PLoS One.

[29] Wu W., Tong X., Liu S., Wang D., Wang L., Fan H. (2019). Prevalence of methicillin-resistant Staphylococcus aureus in healthy Chinese population: a system review and meta-analysis. PLoS One.

[30] Alividza V., Mariano V., Ahmad R., Charani E., Rawson T.M., Holmes A.H., Castro-Sanchez E. (2018). Investigating the impact of poverty on colonization and infection with drug-resistant organisms in humans: a systematic review. Infect. Dis. Povert..

[31] Ang H., Sun X. (2018). Risk factors for multidrug-resistant gram-negative bacteria infection in intensive care units: a meta-analysis. Int. J. Nurs. Pract..

[32] Bert F., Previti C., Calabrese F., Scaioli G., Siliquini R. (2022). Antibiotics self medication among children: A Systematic Review. Antibiotics (Basel).

[33] Bokhary H., Pangesti K.N.A., Rashid H., Abd El Ghany M., Hill-Cawthorne G.A. (2021). Travel-Related Antimicrobial Resistance: A Systematic Review. Trop. Med. Ingect. Dis..

[34] Chatterjee A., Modarai M., Naylor N.R., Boyd S.E., Atun R., Barlow J., Holmes A.H., Johnson A., Robotham J.V. (2018). Quantifying drivers of antibiotic resistance in humans: a systematic review. Lancet Infect. Dis..

[35] Chokshi A., Sifri Z., Cennimo D., Horng H. (2019). Global contributors to antibiotic resistance. J. Global Infect. Dis..

[36] Godijk N.G., Bootsma M.C.J., Bonten M.J.M. (2022). Transmission routes of antibiotic resistant bacteria: a systematic review. BMC Infect. Dis..

[37] Wuerz T.C., Kassim S.S., Atkins K.E. (2020). Acquisition of extended-spectrum beta-lactamase-producing Enterobacteriaceae (ESBL-PE) carriage after exposure to systemic antimicrobials during travel: systematic review and meta-analysis. Travel Med. Infect. Dis..

[38] Zhu D.-M., Li Q.-H., Shen Y., Zhang Q. (2020). Risk factors for quinolone-resistant Escherichia coli infection: a systematic review and meta-analysis. Antimicrob. Resist. Infect. Control.

[39] Chiang H.-Y., Perencevich E.N., Nair R., Nelson R.E., Samore M., Khader K., Chorazy M.L., Herwaldt L.A., Blevins A., Ward M.A., Schweizer M.L. (2017). Incidence and outcomes associated with infections caused by vancomycin-resistant enterococci in the United States: systematic literature review and Meta-analysis. Infect. Control Hosp. Epidemiol..

[40] Mitevska E., Wong B., Surewaard B.G.J., Jenne C.N. (2021). The prevalence, risk, and management of methicillin-resistant Staphylococcus aureus infection in diverse populations across Canada: a systematic review. Pathogens.

[41] de Las M., de la Cruz M.V., Giesen C., Diaz-Menendez M. (2022). International travels and transmission of multidrug resistant Neisseria gonorrhoeae in Europe: a systematic review. Travel Med. Infect. Dis..

[42] Nellums L.B., Thompson H., Holmes A., Castro-Sanchez E., Otter J.A., Norredam M., Friedland J.S., Hargreaves S. (2018). Antimicrobial resistance among migrants in Europe: a systematic review and meta-analysis. Lancet Infect. Dis..

[43] Kariuki S., Kering K., Wairimu C., Onsare R., Mbae C. (2022). Antimicrobial resistance rates and surveillance in sub-Saharan Africa: where are we now?. Infect. Drug Resist..

[44] Mate I., Come C.E., Gonçalves M.P., Cliff J., Gudo E.S. (2019). Knowledge, attitudes and practices regarding antibiotic use in Maputo City, Mozambique. PLoS One.

[45] Peng D., Wang X., Xu Y., Sun C., Zhou X. (2018). Antibiotic misuse among university students in developed and less developed regions of China: a cross-sectional survey. Glob. Health Action.

[46] Manageiro V., Ferreira E., Jones-Dias D., Louro D., Pinto M., Diogo J., Caniça M. (2012). Emergence and risk factors of β-lactamase-mediated resistance to oxyimino-β-lactams in Enterobacteriaceae isolates. Diagn. Microbiol. Infect. Dis..

[47] Schneider S., Salm F., Schröder C., Ludwig N., Hanke R., Gastmeier P. (2016). Antibiotikaeinnahme und Resistenzentwicklung – Wissen, Erfahrungen und Einnahmeverhalten innerhalb der deutschen Allgemeinbevölkerung. Bundesgesundheitsbl.

[48] Skiest D.J., Brown K., Cooper T.W., Hoffman-Roberts H., Mussa H.R., Elliott A.C. (2007). Prospective comparison of methicillin-susceptible and methicillin-resistant community-associated Staphylococcus aureus infections in hospitalized patients. J. Inf. Secur..

[49] Hota B., Ellenbogen C., Hayden M.K., Aroutcheva A., Rice T.W., Weinstein R.A. (2007). Community-associated methicillin-resistant Staphylococcus aureus skin and soft tissue infections at a public hospital: do public housing and incarceration amplify transmission?. Arch. Intern. Med..

[50] Fritz S.A., Garbutt J., Elward A., Shannon W., Storch G.A. (2008). Prevalence of and risk factors for community-acquired methicillin-resistant and methicillin-sensitive staphylococcus aureus colonization in children seen in a practice-based research network. Pediatrics.

[51] V. Li, L. Chui, K. Simmonds, T. Nguyen, G.R. Golding, W. Yacoub, C. Ferrato, M. Louie, Emergence of New CMRSA7/USA400 Methicillin-Resistant *Staphylococcus aureus* spa Types in Alberta, Canada, from 2005 to 2012, J. Clin. Microbiol. 52 (2020) 2439–2446. doi:10.1128/jcm.00505-14. PMC4097756 24789179

[52] Jeong D., Nguyen H.N.T., Tyndall M., Schreiber Y.S. (2020). Antibiotic use among twelve Canadian first nations communities: a retrospective chart review of skin and soft tissue infections. BMC Infect. Dis..

[53] McCarthy N.L., Sullivan P.S., Gaynes R., Rimland D. (2010). Health care-associated and community-associated methicillin-resistant Staphylococcus aureus infections: a comparison of definitions. Am. J. Infect. Control.

[54] T. Lam, K. Lam, P. Ho, W. Yung. Knowledge, attitude, and behaviour toward antibiotics among Hong Kong people: local-born versus immigrants. Hong Kong Med J. 2015 Dec;21 Suppl 7:S41-7. PMID: 26908273. 26908273

[55] Gilbert M., MacDonald J., Gregson D., Siushansian J., Zhang K., Elsayed S., Laupland K., Louie T., Hope K., Mulvey M., Gillespie J., Nielsen D., Wheeler V., Louie M., Honish A., Keays G., Conly J. (2006). Outbreak in Alberta of community-acquired (USA300) methicillin-resistant Staphylococcus aureus in people with a history of drug use, homelessness or incarceration. CMAJ.

[56] Haley C.C., Mittal D., Laviolette A., Jannapureddy S., Parvez N., Haley R.W. (2007). Methicillin-resistant Staphylococcus aureus infection or colonization present at hospital admission: multivariable risk factor screening to increase efficiency of surveillance culturing. J. Clin. Microbiol..

[57] Huang H., Cohen S.H., King J.H., Monchaud C., Nguyen H., Flynn N.M. (2008). Injecting drug use and community-associated methicillin-resistant Staphylococcus aureus infection. Diagn. Microbiol. Infect. Dis..

[58] Chen S.-Y., Wang J.-L., Chen T.H.-H., Chiang W.-C., Wang J.-T., Chen S.-C., Chang S.-C., Hsueh P.-R. (2010). Differences between methicillin-resistant Staphylococcus aureus bacteremic isolates harboring type IV and type V staphylococcal cassette chromosome mec genes based on prior patient healthcare exposure. Eur. J. Clin. Microbiol. Infect. Dis..

[59] Jenkins T.C., Sakai J., Knepper B.C., Swartwood C.J., Haukoos J.S., Long J.A., Price C.S., Burman W.J. (2012). Risk factors for drug-resistant Streptococcus pneumoniae and antibiotic prescribing practices in outpatient community-acquired pneumonia. Acad. Emerg. Med..

[60] Morris S.R., Knapp J.S., Moore D.F., Trees D.L., Wang S.A., Bolan G., Bauer H.M. (2008). Using strain typing to characterise a fluoroquinolone-resistant Neisseria gonorrhoeae transmission network in southern California. Sex. Transm. Infect..

[61] Mathews W.C., Caperna J.C., Barber R.E., Torriani F.J., Miller L.G., May S., McCutchan J.A. (2005). Incidence of and risk factors for clinically significant methicillin-resistant Staphylococcus aureus infection in a cohort of HIV-infected adults. J. Acquir. Immune Defic. Syndr..

[62] Sun C., Hu Y.J., Wang X., Lu J., Lin L., Zhou X. (2019). Influence of leftover antibiotics on self-medication with antibiotics for children: a cross-sectional study from three Chinese provinces. BMJ Open.

[63] Xu Y., Lu J., Sun C., Wang X., Hu Y.J., Zhou X. (2020). A cross-sectional study of antibiotic misuse among Chinese children in developed and less developed provinces. J. Infect. Devel. Countries.

[64] Li R., Xiao F., Zheng X., Yang H., Wang L., Yin D., Yin T., Xin Q., Chen B. (2016). Antibiotic misuse among children with diarrhea in China: results from a national survey. PeerJ.

[65] Pan H., Cui B., Zhang D., Farrar J., Law F., Ba-Thein W. (2012). Prior knowledge, Older Age, and Higher Allowance Are Risk Factors for Self-Medication with Antibiotics among University Students in Southern China. PLoS One.

[66] Ogunshe A., Adinmonyema P. (2014). Evaluation of bacteriostatic potency of expired oral paediatric antibiotics and implications on infant health. Pan Afr. Med. J..

[67] Okeke E.S., Chukwudozie K.I., Nyaruaba R., Ita R.E., Oladipo A., Ejeromedoghene O., Atakpa E.O., Agu C.V., Okoye C.O. (2022). Antibiotic resistance in aquaculture and aquatic organisms: a review of current nanotechnology applications for sustainable management. Environ. Sci. Pollut. Res. Int..

[68] Wozniak T.M., Cuningham W., Ledingham K., McCulloch K. (2022). Contribution of socio-economic factors in the spread of antimicrobial resistant infections in Australian primary healthcare clinics. J. Glob. Antimicrob. Resistan..

[69] Nguyen-Thanh L., Wernli D., Målqvist M., Graells T., Jørgensen P.S. (2024). Characterising proximal and distal drivers of antimicrobial resistance: an umbrella review. J. Glob. Antimicrob. Resistan..

[70] Collignon P., Beggs J.J., Walsh T.R., Gandra S., Laxminarayan R. (2018). Anthropological and socioeconomic factors contributing to global antimicrobial resistance: a univariate and multivariable analysis. The Lancet Planet. Health.

[71] Cooper L.N., Beauchamp A.M., Ingle T.A., Diaz M.I., Wakene A.D., Katterpalli C., Keller T., Walker C., Blumberg S., Kanjilal S., Chen J.H., Radunsky A.P., Most Z.M., Hanna J.J., Perl T.M., Lehmann C.U., Medford R.J. (2024). Socioeconomic disparities and the prevalence of antimicrobial resistance. Clin. Infect. Dis..

[72] Orlando V., Monetti V.M., Juste A.M., Russo V., Mucherino S., Trama U., Guida A., Menditto E. (2020). Drug utilization pattern of antibiotics: the role of age, sex and municipalities in determining variation. RMHP.

[73] Founou L.L., Founou R.C., Essack S.Y. (2021). Antimicrobial resistance in the farm-to-plate continuum: more than a food safety issue. Future Sci. OA.

[74] Allel K., Day L., Hamilton A., Lin L., Furuya-Kanamori L., Moore C.E., Boeckel T.V., Laxminarayan R., Yakob L. (2023). Global antimicrobial-resistance drivers: an ecological country-level study at the human–animal interface. The Lancet Planet. Health.

[75] Thornber K., Bashar A., Ahmed Md.S., Bell A., Trew J., Hasan M., Hasan N.A., Alam Md.M., Chaput D.L., Haque M.M., Tyler C.R. (2022). Antimicrobial resistance in aquaculture environments: unravelling the complexity and connectivity of the underlying societal drivers. Environ. Sci. Technol..

[76] Gautron J.M.C., Tu Thanh G., Barasa V., Voltolina G. (2023). Using intersectionality to study gender and antimicrobial resistance in low- and middle-income countries. Health Policy Plan..

[77] Saint V., Mohsenpour A., Mühling C., Bozorgmehr K. (2020). Exploring equity, gender and social determinants aspects of antimicrobial resistance: scoping review. Eur. J. Pub. Health.

[78] Schröder W., Sommer H., Gladstone B.P., Foschi F., Hellman J., Evengard B., Tacconelli E. (2016). Gender differences in antibiotic prescribing in the community: a systematic review and meta-analysis. J. Antimicrob. Chemother..

[79] Lio R. Magnano San, Favara G., Maugeri A., Barchitta M., Agodi A. (2023). How antimicrobial resistance is linked to climate change: an overview of two intertwined global challenges. Int. J. Environ. Res. Public Health.

[80] Reverter M., Sarter S., Caruso D., Avarre J.-C., Combe M., Pepey E., Pouyaud L., Vega-Heredía S., de Verdal H., Gozlan R.E. (2020). Aquaculture at the crossroads of global warming and antimicrobial resistance. Nat. Commun..

[81] van Bavel B., Berrang-Ford L., Moon K., Gudda F., Thornton A.J., Robinson R.F.S., King R. (2024). Intersections between climate change and antimicrobial resistance: a systematic scoping review. The Lancet Planet. Health.

[82] Fernández Salgueiro M., Cernuda Martínez J.A., Gan R.K., Arcos González P. (2024). Climate change and antibiotic resistance: a scoping review. Environ. Microbiol. Rep..

[83] Collignon P., Athukorala P., Senanayake S., Khan F. (2015). Antimicrobial resistance: the major contribution of poor governance and corruption to this growing problem. PLoS One.

[84] Rönnerstrand B., Lapuente V. (2017). Corruption and use of antibiotics in regions of Europe. Health Polic..

[85] Maugeri A., Barchitta M., Agodi A. (2023). Association between quality of governance, antibiotic consumption, and antimicrobial resistance: an analysis of Italian regions. Antimicrob. Resist. Infect. Control.

[86] Barathe P., Kaur K., Reddy S., Shriram V., Kumar V. (2024). Antibiotic pollution and associated antimicrobial resistance in the environment. J. Hazard. Mater. Lett..

[87] Zhou Z., Shuai X., Lin Z., Yu X., Ba X., Holmes M.A., Xiao Y., Gu B., Chen H. (2023). Association between particulate matter (PM)2·5 air pollution and clinical antibiotic resistance: a global analysis. The Lancet Planet. Health.

[88] Sorn S., Sulfikar M.-Y., Lin M., Shuto M., Noguchi R., Honda R., Yamamoto-Ikemoto T. (2022). Watanabe, potential impact factors on the enhancement of antibiotic resistance in a lake environment. J. Water Health.

[89] Abbara A., Rawson T.M., Karah N., El-Amin W., Hatcher J., Tajaldin B., Dar O., Dewachi O., Abu Sitta G., Uhlin B.E., Sparrow A. (2018). A summary and appraisal of existing evidence of antimicrobial resistance in the Syrian conflict. Int. J. Infect. Dis..

[90] Granata G., Petersen E., Capone A., Donati D., Andriolo B., Gross M., Cicalini S., Petrosillo N. (2024). The impact of armed conflict on the development and global spread of antibiotic resistance: a systematic review. Clin. Microbiol. Infect..

[91] Abbara A., Rawson T.M., Karah N., El-Amin W., Hatcher J., Tajaldin B., Dar O., Dewachi O., Abu Sitta G., Uhlin B.E., Sparrow A. (2018). Antimicrobial resistance in the context of the Syrian conflict: drivers before and after the onset of conflict and key recommendations. Int. J. Infect. Dis..

[92] Kanapathipillai R., Malou N., Hopman J., Bowman C., Yousef N., Michel J., Hussein N., Herard P., Ousley J., Mills C., Seguin C., Saim M. (2019). Antibiotic resistance in conflict settings: lessons learned in the Middle East. JAC-Antimicrob. Resistan..

[93] Silva A.C., Nogueira P.J., Paiva J.-A. (2021). Determinants of antimicrobial resistance among the different European countries: more than human and animal antimicrobial consumption. Antibiotics.

[94] Collignon P. (2015). Antibiotic resistance: are we all doomed?. Intern. Med. J..

[95] Collignon P., Beggs J.J. (2019). Socioeconomic enablers for contagion: factors impelling the antimicrobial resistance epidemic. Antibiotics.

[96] Rousham E.K., Nahar P., Uddin M.R., Islam M.A., Nizame F.A., Khisa N., Akter S.M.S., Munim M.S., Rahman M., Unicomb L. (2023). Gender and urban-rural influences on antibiotic purchasing and prescription use in retail drug shops: a one health study. BMC Public Health.

[97] Vassallo A., Kett S., Purchase D., Marvasi M. (2022). The bacterial urban Resistome: recent advances. Antibiotics (Basel).

