# Supplementary material for: Mapping socioeconomic factors driving antimicrobial resistance in humans: An umbrella review
Source: One Health. 2025 Feb 10;20:100986. doi: 10.1016/j.onehlt.2025.100986 (PMC11872410; doi:10.1016/j.onehlt.2025.100986)
Supplement: Supplementary file 1 — Supplementary material: PRIOR checklist; Overview of the search string; Descriptive table of included articles; Quality assessment of reviewed articles; List of excluded articles at full-text review; Detailed list of drivers; Full quantitative data extraction table [file mmc1.docx]

**Supplementary Material**

Supplementary Table 1: PRIOR checklist

| **Section**  Topic | **#** | **Item** | **Location reported** |
| --- | --- | --- | --- |
| **TITLE** | | |  |
| Title | 1 | Identify the report as an overview of reviews. | Page 1 |
| **ABSTRACT** | | |  |
| Abstract | 2 | Provide a comprehensive and accurate summary of the purpose, methods, and results of the overview of reviews. | Page 2 |
| **INTRODUCTION** | | |  |
| Rationale | 3 | Describe the rationale for conducting the overview of reviews in the context of existing knowledge. | Page 3 |
| Objectives | 4 | Provide an explicit statement of the objective(s) or question(s) addressed by the overview of reviews. | Page 3 |
| **METHODS** | | |  |
| Eligibility criteria | 5a | Specify the inclusion and exclusion criteria for the overview of reviews. If supplemental primary studies were included, this should be stated, with a rationale. | Pages 3-4 |
|  | 5b | Specify the definition of ‘systematic review’ as used in the inclusion criteria for the overview of reviews. | Page 3 |
| Information sources | 6 | Specify all databases, registers, websites, organizations, reference lists, and other sources searched or consulted to identify systematic reviews and supplemental primary studies (if included).  Specify the date when each source was last searched or consulted. | Page 4 |
| Search strategy | 7 | Present the full search strategies for all databases, registers and websites, such that they could be reproduced. Describe any search filters and limits applied. | Page 4, Supplementary eTable 2 |
| Selection process | 8a | Describe the methods used to decide whether a systematic review or supplemental primary study (if included) met the inclusion criteria of the overview of reviews. | Page 4 |
|  | 8b | Describe how overlap in the populations, interventions, comparators, and/or outcomes of systematic reviews was identified and managed during study selection. | NA |
| Data collection process | 9a | Describe the methods used to collect data from reports. | Page 4 |
|  | 9b | If applicable, describe the methods used to identify and manage primary study overlap at the level  of the comparison and outcome during data collection. For each outcome, specify the method used to illustrate and/or quantify the degree of primary study overlap across systematic reviews. | NA |
|  | 9c | If applicable, specify the methods used to manage discrepant data across systematic reviews during data collection. | NA |
| Data items | 10 | List and define all variables and outcomes for which data were sought. Describe any assumptions made and/or measures taken to identify and clarify missing or unclear information. | NA |
| Risk of bias assessment | 11a | Describe the methods used to *assess* risk of bias or methodological quality of the included systematic reviews. | NA |
|  | 11b | Describe the methods used to *collect* data on (from the systematic reviews) and/or *assess* the risk of bias of the primary studies included in the systematic reviews. Provide a justification for instances where flawed, incomplete, or missing assessments are identified but not re-assessed. | NA |
|  | 11c | Describe the methods used to *assess* the risk of bias of supplemental primary studies (if included). | NA |
| Synthesis methods | 12a | Describe the methods used to summarize or synthesize results and provide a rationale for the choice(s). | Page 4 |
|  | 12b | Describe any methods used to explore possible causes of heterogeneity among results. | NA |
|  | 12c | Describe any sensitivity analyses conducted to assess the robustness of the synthesized results. | NA |
| Reporting bias assessment | 13 | Describe the methods used to *collect* data on (from the systematic reviews) and/or *assess* the risk of bias due to missing results in a summary or synthesis (arising from reporting biases at the levels of the systematic reviews, primary studies, and supplemental primary studies, if included). | NA |
| Certainty assessment | 14 | Describe the methods used to *collect* data on (from the systematic reviews) and/or *assess* certainty (or confidence) in the body of evidence for an outcome. | NA |
| **RESULTS** | | |  |
| Systematic review and supplemental primary study selection | 15a | Describe the results of the search and selection process, including the number of records screened, assessed for eligibility, and included in the overview of reviews, ideally with a flow diagram. | Pages 4-5 |
|  | 15b | Provide a list of studies that might appear to meet the inclusion criteria, but were excluded, with the main reason for exclusion. | Page 5 |

| **Section**  Topic | **#** | **Item** | **Location reported** |
| --- | --- | --- | --- |
| Characteristics of systematic reviews and supplemental primary studies | 16 | Cite each included systematic review and supplemental primary study (if included) and present its characteristics. | Supplementary eTable 3 |
| Primary study overlap | 17 | Describe the extent of primary study overlap across the included systematic reviews. | NA |
| Risk of bias in systematic reviews, primary studies, and  supplemental primary studies | 18a | Present assessments of risk of bias or methodological quality for each included systematic review. | NA |
|  | 18b | Present assessments (*collected* from systematic reviews or *assessed* anew) of the risk of bias of the primary studies included in the systematic reviews. | NA |
|  | 18c | Present assessments of the risk of bias of supplemental primary studies (if included). | NA |
| Summary or synthesis of results | 19a | For all outcomes, summarize the evidence from the systematic reviews and supplemental primary studies (if included). If meta-analyses were done, present for each the summary estimate and its precision and measures of statistical heterogeneity. If comparing groups, describe the direction of the effect. | Pages 5-9 |
|  | 19b | If meta-analyses were done, present results of all investigations of possible causes of heterogeneity. | NA |
|  | 19c | If meta-analyses were done, present results of all sensitivity analyses conducted to assess the robustness of synthesized results. | NA |
| Reporting biases | 20 | Present assessments (*collected* from systematic reviews and/or *assessed* anew) of the risk of bias due to missing primary studies, analyses, or results in a summary or synthesis (arising from reporting biases at the levels of the systematic reviews, primary studies, and supplemental primary  studies, if included) for each summary or synthesis assessed. | NA |
| Certainty of evidence | 21 | Present assessments (*collected* or *assessed* anew) of certainty (or confidence) in the body of evidence for each outcome. | NA |
| **DISCUSSION** | | |  |
| Discussion | 22a | Summarize the main findings, including any discrepancies in findings across the included systematic reviews and supplemental primary studies (if included). | Pages 9-10 |
|  | 22b | Provide a general interpretation of the results in the context of other evidence. | Page 10 |
|  | 22c | Discuss any limitations of the evidence from systematic reviews, their primary studies, and supplemental primary studies (if included) included in the overview of reviews. Discuss any limitations of the overview of reviews methods used. | Page 10 |
|  | 22d | Discuss implications for practice, policy, and future research (both systematic reviews and primary research). Consider the relevance of the findings to the end users of the overview of reviews, e.g., healthcare providers, policymakers, patients, among others. | Pages 10-11 |
| **OTHER INFORMATION** | | |  |
| Registration and protocol | 23a | Provide registration information for the overview of reviews, including register name and registration number, or state that the overview of reviews was not registered. | NA |
|  | 23b | Indicate where the overview of reviews protocol can be accessed, or state that a protocol was not prepared. | NA |
|  | 23c | Describe and explain any amendments to information provided at registration or in the protocol. Indicate the stage of the overview of reviews at which amendments were made. | NA |
| Support | 24 | Describe sources of financial or non-financial support for the overview of reviews, and the role of the funders or sponsors in the overview of reviews. |  |
| Competing interests | 25 | Declare any competing interests of the overview of reviews' authors. |  |
| Author information | 26a | Provide contact information for the corresponding author. | Page 1 |
|  | 26b | Describe the contributions of individual authors and identify the guarantor of the overview of reviews. |  |
| Availability of data and other materials | 27 | Report which of the following are available, where they can be found, and under which conditions they may be accessed: template data collection forms; data collected from included systematic reviews and supplemental primary studies; analytic code; any other materials used in the overview of reviews. |  |

Supplementary Table 2: Overview of the search string

| **Database** | **Search string** | **Hits** |
| --- | --- | --- |
| MEDLINE | 1            (AMR or ABR or AFR or (antibiotic* adj3 resist*) or ((anti?microbial* or "anti microbial*") adj3 resist*) or ((anti?bacterial* or "anti bacterial*") adj3 resist*) or ((anti?fungal* or "anti fungal*") adj3 resist*) or ((anti?viral* or "anti viral*") adj3 resist*) or (microb* adj3 resist*)).ti,ab.  2            exp Drug Resistance, Bacterial/ or exp Drug Resistance, Viral/ or exp Drug Resistance, Fungal/ or exp Drug Resistance, Microbial/  3            or/1-2  4            (socio?economic* or social* or economic* or inequalit* or inequit* or poverty or corrupt* or financ*).ti,ab.  5            exp Low Socioeconomic Status/ or exp Socioeconomic Disparities in Health/ or exp Socioeconomic Factors/ or exp Social Status/ or exp Social Class/ or exp Health Inequities/ or exp Poverty/ or exp Child Poverty/ or exp Poverty Areas/ or exp Health Status Disparities/ or exp Healthcare Disparities/  6            (employ* or unemploy* or occupation* or income or salar* or pay).ti,ab.  7            exp Workforce/ or exp Employment/ or exp "Salaries and Fringe Benefits"/  8            (education* or school* or tuition* or training* or literacy).ti,ab.  9            exp Educational Status/ or exp Literacy/ or exp Health Literacy/ or exp Information Literacy/ or exp Computer Literacy/ or exp Health Education/  10          (house* or housing or residen* or home* or neighbo?rhood*).ti,ab.  11          exp Housing/ or exp Residence Characteristics/ or exp Catchment Area, Health/ or exp Home Environment/ or exp Neighborhood Characteristics/  12          ("Health* Seek*" or "Seek* Behav*" or (Health* adj3 Access*) or (Health* adj3 afford*) or "Health* insur*" or Self?medicat* or "self medicat*").ti,ab.  13          exp Health Knowledge, Attitudes, Practice/ or exp Health Behavior/ or exp Health Services Accessibility/ or exp Insurance, Health/ or exp Insurance Coverage/ or exp Self Medication/ or exp Nonprescription Drugs/  14          (ethnic* or race* or racis* or "minorit* group*" or racial* or asian* or black* or white* or latin* or hispanic* or "African American*" or Afro?caribbean* or "Afro Caribbean*" or Caribbean* or "Afro Latin*" or "American Indian*" or "Han Chinese" or "Native Hawaiian*" or Caucasian* or "Native American* Alaskan Native*" or "Pacific Islander*" or "Mixed Race" or "African Ancestry" or Roma or Gypsy or Aborigin* or Indigen* or "First Nation*" or "Middle Eastern" or Jew* or Hindu* or Islam* or Muslim* or Sikh* or BME or BAME or POC).ti,ab.  15          exp Ethnicity/ or exp "Ethnic and Racial Minorities"/ or exp Racial Groups/ or exp White People/ or exp "Black or African American"/ or exp Black People/ or exp Southeast Asian/ or exp Asian People/ or exp West Asian People/ or exp Southeast Asian People/ or exp Central Asian People/ or exp East Asian People/ or exp Asian/ or exp South Asian People/ or exp Alaskan Natives/ or exp "American Indian or Alaska Native"/ or exp "Hispanic or Latino"/ or exp "Native Hawaiian or Other Pacific Islander"/ or exp Pacific Island People/ or exp Arabs/ or exp Jews/ or exp Indigenous Peoples/ or exp "Australian Aboriginal and Torres Strait Islander Peoples"/ or exp Indians, North American/ or exp Roma/ or exp Indians, South American/  16          or/4-15  17          (One?health or "One Health" or Sanitation* or Hygien* or EcoHealth or "One World" or "One Medicine" or Stewards* or Governance or Zoono* or animal-human or human-animal or environment-human or human-environment or animal-environment or environment-animal or "Environmental Health" or ecosystem* or soil or wastewater or surfacewater or groundwater or "drinking water" or manure or biosolid*).ti,ab.  18          exp One Health/ or exp Zoonoses/ or exp Environmental Health/ or exp Ecosystem/ or exp Soil/ or exp Soil Microbiology/ or exp Wastewater/ or exp Groundwater/ or exp manure/ or exp Drinking Water/ or exp Biosolids/  19          (Agriculture or Livestock or Farm* or veterinar* or Bovine or Cattle or Dairy or Beef or Feedlot or Cow* or Sheep or Lamb* or Goat* or Dog or Dogs or Canine* or Cat or Cats or Feline* or "Domestic Animal*" or "Companion Animal*" or Swine or Hog* or Pig or Pigs or Porcine or Pork or Caprine or Ovine or Horse* or Equine or Poultry or Turkey* or Broiler or Hen or Hens or Duck* or Flock* or Avian or Chicken* or "Animal Health" or "Animal Welfare" or "Animal Production*" or Fish or Aquaculture or Tilapia or Shrimp or Mussel* or Salmon or Albacore or Trout* or Seafood or carp or "Cat Fish" or catfish or Shellfish or Mollusk* or Clams or Oyster* or Scallop* or Walleye or Perch or Halibut or Cod or "Sea Bass" or Tuna or "Farmed Fish" or Mink or Rabbit* or Lepus or Hare or Buffalo or Bison or "Bos Taurus" or "Bos Indicus" or elk or Deer or Cervid* or Camel* or Wildlife or Zoo or Carcass* or Abattoir* or Slaughter*).ti,ab.  20          exp Agriculture/ or exp Observational Study, Veterinary/ or exp Veterinary Drugs/ or exp Veterinary Medicine/ or exp Animal Diseases/ or exp Livestock/ or exp Animals/ or exp Cattle/ or exp Cattle Diseases/ or exp Goats/ or exp Cats/ or exp Dogs/ or exp Animals, Domestic/ or exp Swine Diseases/ or exp Horses/ or exp Chickens/ or exp Poultry/ or exp Poultry Diseases/ or exp Animal Husbandry/ or exp Animal Welfare/ or exp Fish Diseases/ or exp Aquaculture/ or exp Camelus/ or exp Animals, Wild/ or exp Abattoirs/  21          or/17-20  22          3 and (16 or (16 and 21))  23          limit 22 to (english language and "review articles" and yr="2010 -Current") | 2915 |
| Embase | 1            (AMR or ABR or AFR or (antibiotic* adj3 resist*) or ((anti?microbial* or "anti microbial*") adj3 resist*) or ((anti?bacterial* or "anti bacterial*") adj3 resist*) or ((anti?fungal* or "anti fungal*") adj3 resist*) or ((anti?viral* or "anti viral*") adj3 resist*) or (microb* adj3 resist*)).ti,ab.  2            exp antibiotic resistance/  3            or/1-2  4            (socio?economic* or social* or economic* or inequalit* or inequit* or poverty or corrupt* or financ*).ti,ab.  5            exp socioeconomics/ or exp social segregation/ or exp social status/ or exp health disparity/ or exp child poverty/ or exp poverty/ or exp health care disparity/  6            (employ* or unemploy* or occupation* or income or salar* or pay).ti,ab.  7            exp Employment/ or exp Employment Status/ or exp Salary/ or exp "Salaries and Fringe Benefits"/  8            (education* or school* or tuition* or training* or literacy).ti,ab.  9            exp Education/ or exp Literacy/ or exp Health Literacy/ or exp Information Literacy/ or exp Computer Literacy/ or exp Health Education/  10          (house* or housing or residen* or home* or neighbo?rhood*).ti,ab.  11          exp Housing/ or exp Residence Characteristics/ or exp "Catchment Area (Health)"/ or exp Home Environment/ or exp Neighborhood Characteristic/  12          ("Health* Seek*" or "Seek* Behav*" or (Health* adj3 Access*) or (Health* adj3 afford*) or "Health* insur*" or Self?medicat* or "self medicat*").ti,ab.  13          exp attitude to health/ or exp Health Behavior/ or exp health care access/ or exp health insurance/ or exp Insurance Coverage/ or exp Self Medication/ or exp Non Prescription Drugs/  14          (ethnic* or race* or racis* or "minorit* group*" or racial* or asian* or black* or white* or latin* or hispanic* or "African American*" or Afro?caribbean* or "Afro Caribbean*" or Caribbean* or "Afro Latin*" or "American Indian*" or "Han Chinese" or "Native Hawaiian*" or Caucasian* or "Native American*" or "Alaskan Native*" or "Pacific Islander*" or "Mixed Race" or "African Ancestry" or Roma or Gypsy or Aborigin* or Indigen* or "First Nation*" or "Middle Eastern" or Jew* or Hindu* or Islam* or Muslim* or Sikh* or BME or BAME or POC).ti,ab.  15          exp Ethnicity/ or exp "Ethnic and Racial Aspects"/ or exp Ethnic Group/ or exp Caucasian/ or exp Black Person/ or exp Southeast Asian/ or exp Asian/ or exp West Asian/ or exp Central Asian/ or exp East Asian/ or exp South Asian/ or exp Alaska Natives/ or exp American Indian/ or exp Hispanic/ or exp Pacific Islander/ or exp Arab/ or exp Jew/ or exp Indigenous People/ or exp "Romani (people)"/  16          or/4-15  17          (One?health or "One Health" or Sanitation* or Hygien* or EcoHealth or "One World" or "One Medicine" or Stewards* or Governance or Zoono* or animal-human or human-animal or environment-human or human-environment or animal-environment or environment-animal or "Environmental Health" or ecosystem* or soil or wastewater or surfacewater or groundwater or "drinking water" or manure or biosolid*).ti,ab.  18          exp One Health/ or exp Zoonosis/ or exp Environmental Health/ or exp Ecosystem/ or exp Soil/ or exp Wastewater/ or exp Ground water/ or exp manure/ or exp Drinking Water/ or exp Biosolid/  19          (Agriculture or Livestock or Farm* or veterinar* or Bovine or Cattle or Dairy or Beef or Feedlot or Cow* or Sheep or Lamb* or Goat* or Dog or Dogs or Canine* or Cat or Cats or Feline* or "Domestic Animal*" or "Companion Animal*" or Swine or Hog* or Pig or Pigs or Porcine or Pork or Caprine or Ovine or Horse* or Equine or Poultry or Turkey* or Broiler or Hen or Hens or Duck* or Flock* or Avian or Chicken* or "Animal Health" or "Animal Welfare" or "Animal Production*" or Fish or Aquaculture or Tilapia or Shrimp or Mussel* or Salmon or Albacore or Trout* or Seafood or carp or "Cat Fish" or catfish or Shellfish or Mollusk* or Clams or Oyster* or Scallop* or Walleye or Perch or Halibut or Cod or "Sea Bass" or Tuna or "Farmed Fish" or Mink or Rabbit* or Lepus or Hare or Buffalo or Bison or "Bos Taurus" or "Bos Indicus" or elk or Deer or Cervid* or Camel* or Wildlife or Zoo or Carcass* or Abattoir* or Slaughter*).ti,ab.  20          exp Agriculture/ or exp Animal Disease/ or exp Veterinary Medicine/ or exp Livestock/ or exp Bovine/ or exp Cattle Disease/ or exp Goat/ or exp Cat/ or exp Dog/ or exp Domestic Animal/ or exp Swine Disease/ or exp Horse/ or exp Chicken/ or exp Poultry/ or exp Bird Disease/ or exp Animal Husbandry/ or exp Animal Welfare/ or exp Fish Disease/ or exp Aquaculture/ or exp Camel/ or exp Wild Animal/ or exp slaughterhouse/  21          or/17-20  22          3 and (16 or (16 and 21))  23          exp review/  24          (literature adj3 review$).ti,ab.  25          exp "Systematic Review"/  26          exp meta analysis/  27          or/23-26  28          22 and 27  29          limit 28 to (english language and yr="2010 -Current") | 4947 |
| Global health | 1            (AMR or ABR or AFR or (antibiotic* adj3 resist*) or ((anti?microbial* or "anti microbial*") adj3 resist*) or ((anti?bacterial* or "anti bacterial*") adj3 resist*) or ((anti?fungal* or "anti fungal*") adj3 resist*) or ((anti?viral* or "anti viral*") adj3 resist*) or (microb* adj3 resist*)).ti,ab.  2            exp antibiotic resistance/  3            or/1-2  4            (socio?economic* or social* or economic* or inequalit* or inequit* or poverty or corrupt* or financ*).ti,ab.  5            exp socioeconomic status/ or exp socioeconomics/ or exp social status/ or exp social classes/ or exp poverty/ or exp health inequalities/  6            (employ* or unemploy* or occupation* or income or salar* or pay).ti,ab.  7            exp low income/ or exp employment/ or exp workers/ or exp salaries/  8            (education* or school* or tuition* or training* or literacy).ti,ab.  9            exp literacy/ or exp education/  10          (house* or housing or residen* or home* or neighbo?rhood*).ti,ab.  11          exp housing/ or exp neighborhoods/  12          ("Health* Seek*" or "Seek* Behav*" or (Health* adj3 Access*) or (Health* adj3 afford*) or "Health* insur*" or Self?medicat* or "self medicat*").ti,ab.  13          exp health behaviour/ or exp health insurance/  14          (ethnic* or race* or racis* or "minorit* group*" or racial* or asian* or black* or white* or latin* or hispanic* or "African American*" or Afro?caribbean* or "Afro Caribbean*" or Caribbean* or "Afro Latin*" or "American Indian*" or "Han Chinese" or "Native Hawaiian*" or Caucasian* or "Native American* Alaskan Native*" or "Pacific Islander*" or "Mixed Race" or "African Ancestry" or Roma or Gypsy or Aborigin* or Indigen* or "First Nation*" or "Middle Eastern" or Jew* or Hindu* or Islam* or Muslim* or Sikh* or BME or BAME or POC).ti,ab.  15          exp ethnic groups/ or exp ethnicity/ or exp black people/ or exp white people/ or exp Hispanics/ or exp ethnic groups/ or exp Asians/ or exp Inuit/ or exp indigenous people/ or exp Alaska Natives/ or exp Native Americans/ or exp Jews/ or exp Asians/ or exp Pacific Islanders/ or exp aborigines/ or exp Roma/  16          or/4-15  17          (One?health or "One Health" or Sanitation* or Hygien* or EcoHealth or "One World" or "One Medicine" or Stewards* or Governance or Zoono* or animal-human or human-animal or environment-human or human-environment or animal-environment or environment-animal or "Environmental Health" or ecosystem* or soil or wastewater or surfacewater or groundwater or "drinking water" or manure or biosolid*).ti,ab.  18          exp zoonoses/ or exp environmental health/ or exp ecosystems/ or exp soil/ or exp soil bacteria/ or exp wastewater/ or exp groundwater/ or exp surface water/ or exp animal manures/ or exp drinking water/  19          (Agriculture or Livestock or Farm* or veterinar* or Bovine or Cattle or Dairy or Beef or Feedlot or Cow* or Sheep or Lamb* or Goat* or Dog* or Canine* or Cat* or Feline* or "Domestic Animal*" or "Companion Animal*" or Swine or Hog* or Pig* or Porcine or Pork or Caprine or Ovine or Horse* or Equine or Poultry or Turkey* or Broiler or Hen or Hens or Duck* or Flock* or Avian or Chicken* or "Animal Health" or "Animal Welfare" or "Animal Production*" or Fish or Aquaculture or Tilapia or Shrimp or Mussel* or Salmon or Albacore or Trout* or Seafood or carp or "Cat Fish" or catfish or Shellfish or Mollusk* or Clams or Oyster* or Scallop* or Walleye or Perch or Halibut or Cod or "Sea Bass" or Tuna or "Farmed Fish" or Mink or Rabbit* or Lepus or Hare or Buffalo or Bison or "Bos Taurus" or "Bos Indicus" or elk or Deer or Cervid* or Camel* or Wildlife or Zoo or Carcass* or Abattoir* or Slaughter*).ti,ab.  20          exp agriculture/ or exp veterinary medicine/ or exp animal diseases/ or exp livestock/ or exp cattle/ or exp cattle diseases/ or exp goats/ or exp cats/ or exp dogs/ or exp domestic animals/ or exp swine diseases/ or exp horses/ or exp fowls/ or exp poultry diseases/ or exp poultry/ or exp animal husbandry/ or exp animal welfare/ or exp fish diseases/ or exp aquaculture/ or exp wild animals/ or exp abattoirs/  21          or/17-20  22          3 and 16  23          21 and 16  24          3 and 23  25          22 or 24  26          (Evidence review or evidence reviews or evidence synthesis or evidence-based review or evidence report or "Evidence Reports - Agency for Healthcare Research and Quality" or publication bias or campbell collaboration).mp. [mp=abstract, title, original title, heading words, cabicodes words]  27          (meta-analysis or meta-analyses or metaanalyses or metaanalysis).mp. [mp=abstract, title, original title, heading words, cabicodes words] | 389 |
| Cochrane library | #1          (AMR OR ABR OR AFR OR (Antibiotic* NEAR/3 Resist*) OR ((Anti?microbial* OR "Anti Microbial*") NEAR/3 Resist*) OR ((Anti?bacterial* OR "Anti bacterial*") NEAR/3 Resist*) OR ((Anti?viral* OR "Anti viral*") NEAR/3 Resist*) OR ((Anti?fungal* OR "Anti fungal*") NEAR/3 Resist*) OR (Microbial* NEAR/3 resist*)):ti,ab  #2          MeSH descriptor: [Drug Resistance, Bacterial] explode all trees  #3          MeSH descriptor: [Drug Resistance, Fungal] explode all trees  #4          MeSH descriptor: [Drug Resistance, Microbial] explode all trees  #5          MeSH descriptor: [Drug Resistance, Viral] explode all trees  #6          #1 OR #2 OR #3 OR #4 OR #5  #7          (Socio?economic* OR social* OR economic* OR inequalit* OR inequit* OR poverty OR corrupt* OR financ*):ti,ab  #8          MeSH descriptor: [Social Class] explode all trees  #9          MeSH descriptor: [Poverty] explode all trees  #10        MeSH descriptor: [Health Status Disparities] explode all trees  #11        MeSH descriptor: [Socioeconomic Factors] explode all trees  #12        MeSH descriptor: [Poverty Areas] explode all trees  #13        #7 OR #8 OR #9 OR #10 OR #11 OR #12  #14        (*employ* OR occupation* OR income OR salar* or pay):ti,ab  #15        MeSH descriptor: [Workforce] explode all trees  #16        MeSH descriptor: [Employment] explode all trees  #17        MeSH descriptor: [Salaries and Fringe Benefits] explode all trees  #18        #14 OR #15 OR #16 OR #17  #19        (education* OR school* OR tuition* OR training OR literacy):ti,ab  #20        MeSH descriptor: [Education] explode all trees  #21        MeSH descriptor: [Literacy] explode all trees  #22        MeSH descriptor: [Health Literacy] explode all trees  #23        MeSH descriptor: [Information Literacy] explode all trees  #24        MeSH descriptor: [Computer Literacy] explode all trees  #25        #19 OR #20 OR #21 OR #22 OR #23 OR #24  #26        (House* OR housing OR residen* OR home* OR neighbo?rhood*):ti,ab  #27        MeSH descriptor: [Housing] explode all trees  #28        MeSH descriptor: [Residence Characteristics] explode all trees  #29        #26 OR #27 OR #28  #30        ((health* NEXT seek*) OR (seek* NEXT behav*) OR (health* NEAR/3 access*) OR (health* NEAR/3 afford*) OR (health* NEXT service*) OR (health* NEXT insur*) OR self?medicat* OR (self NEXT medicat*)):ti,ab  #31        MeSH descriptor: [Attitude to Health] explode all trees  #32        MeSH descriptor: [Health Services] explode all trees  #33        MeSH descriptor: [Insurance, Health] explode all trees  #34        MeSH descriptor: [Health Services Accessibility] explode all trees  #35        #30 OR #31 OR #32 OR #33 OR #34  #36        (ethnic* OR race* OR racis* OR (minorit* NEXT group*) OR racial* OR asian* OR black* OR white* OR latin* OR hispanic* OR (African NEXT American*) OR afro?caribbean* OR (Afro NEXT Caribbean*) OR caribbean* OR (American NEXT Indian) OR (Han NEXT Chinese) OR (Native NEXT Hawaiian*) OR caucasian* OR (Alaska* NEXT Native*) OR (Pacific NEXT Islander*) OR (mixed NEXT race*) OR (african NEXT ancestry) OR Roma OR Gypsy OR aborigin* OR indigen* OR (First NEXT Nation*) OR BME OR BAME OR (Middle NEXT Eastern) OR POC OR Jew* OR Islam* OR Muslim* OR Sikh* OR Hindu*):ti,ab  #37        MeSH descriptor: [Minority Health] explode all trees  #38        MeSH descriptor: [Minority Groups] explode all trees  #39        MeSH descriptor: [Racial Groups] explode all trees  #40        MeSH descriptor: [Black People] explode all trees  #41        MeSH descriptor: [Asian People] explode all trees  #42        MeSH descriptor: [North American People] explode all trees  #43        MeSH descriptor: [Asian American Native Hawaiian and Pacific Islander] explode all trees  #44        MeSH descriptor: [Indigenous Peoples] explode all trees  #45        #36 OR #37 OR #38 OR #39 OR #40 OR #41 OR #42 OR #43 OR #44  #46        #13 OR #18 OR #25 OR #29 OR #35 OR #45  #47        (One?Health OR (One NEXT Health) OR sanitation* OR hygien* OR eco health OR (One NEXT World) OR (One NEXT Medicine) OR stewards* OR governance OR ozone* OR animal-human OR human-animal OR human-environment OR environment-human OR environment-animal OR animal-environment OR (environmental NEXT health) OR ecosystem* OR soil OR wastewater OR groundwater OR manure OR (drinking NEXT water) OR biosolid*):ti,ab  #48        MeSH descriptor: [Environmental Health] explode all trees  #49        MeSH descriptor: [Ecosystem] explode all trees  #50        MeSH descriptor: [Soil] explode all trees  #51        MeSH descriptor: [Drinking Water] explode all trees  #52        #47 OR #48 OR #49 OR #50 OR #51  #53        (Agriculture* OR livestock OR farm* OR veterinar* OR bovine OR cattle or dairy OR beef OR feedlot OR cow* OR sheep OR lamb* OR goat* OR (dog OR dogs) OR canine* OR (cat OR cats) OR feline* OR (domestic NEXT animal*) OR (companion NEXT animal*) OR swine OR hog OR (pig OR pigs) OR porcine OR ovine OR horse* OR equine OR poultry OR turkey* OR broiler OR (hen OR hens) OR duck OR flock OR avian OR chicken* OR (animal NEXT welfare) OR (animal NEXT health) OR (animal NEXT production) OR fish OR aquaculture OR tilapia OR shrimp OR mussel* OR salmon OR albacore OR trout* OR seafood OR carp OR catfish OR shellfish OR mollusk* OR clams OR oyster* OR scallop* OR perch OR halibut OR cod OR (sea NEXT bass) OR tuna OR (farmed NEXT fish) OR mink OR rabbit* OR Lepus OR hare OR buffalo OR bison OR (bos NEXT (taurus OR indicus)) OR elk OR deer OR cervid* OR camel* OR wildlife OR zoo OR carcass* OR abattoir* OR slaughter*):ti,ab  #54        MeSH descriptor: [Agriculture] explode all trees  #55        MeSH descriptor: [Animal Diseases] explode all trees  #56        MeSH descriptor: [Cattle] explode all trees  #57        MeSH descriptor: [Cats] explode all trees  #58        MeSH descriptor: [Dogs] explode all trees  #59        MeSH descriptor: [Animals, Domestic] explode all trees  #60        MeSH descriptor: [Horses] explode all trees  #61        MeSH descriptor: [Poultry] explode all trees  #62        MeSH descriptor: [Animal Husbandry] explode all trees  #63        MeSH descriptor: [Fish Diseases] explode all trees  #64        #53 OR #54 OR #55 OR #56 OR #57 OR #58 OR #59 OR #60 OR #61 OR #62 OR #63  #65        #52 OR #64  #66        #6 AND #46  #67        #46 AND #65  #68        #6 AND #67  #69        #66 OR #68 with Cochrane Library publication date Between Jan 2010 and Mar 2023, in Cochrane Reviews (Word variations have been searched) | 85 |

Supplementary Table 3: Descriptive table of included articles

| **Title** | **First author** | **Regions** | **Reviewed Years** | **Research Question** | **One Health** | **# articles** |
| --- | --- | --- | --- | --- | --- | --- |
| **Investigating the impact of poverty on colonization and infection with drug-resistant organisms in humans: a systematic review (1)** | Vivian Alividza | AMR, EUR, AFR, EMR, SEAR, WPR | 1998-2015 | What is the relationship between poverty and AMR? | Human; Environment | 19 |
| **Risk factors for multidrug-resistant Gram-negative bacteria infection in intensive care units: A meta-analysis (2)** | Hui Ang | AMR, EUR, AFR, EMR, SEAR, WPR | 2007-2016 | What are the risk factors for MDR GNB in ICUs | Human | 18 |
| **Antibiotics Self Medication among Children: A Systematic Review (3)** | Fabrizio Bert | AMR, EUR, AFR, EMR, SEAR, WPR | up to 2022 | What factors influence self-medication with antibiotics of children | Human | 57 |
| **Travel-Related Antimicrobial Resistance: A Systematic Review (4)** | Hamid Bokhary | AMR, EUR, AFR, EMR, SEAR, WPR | up to 2019 | What is the impact of travel on AMR dissemination | Human | 238 |
| **A Systematic Review and Meta-analysis of Ventilator-associated Pneumonia in Adults in Asia: An Analysis of National Income Level on Incidence and Etiology (5)** | Ana Bonell | EMR, SEAR, WPR | 1990-2017 | Estimate incidence, prevalence, and etiology of Ventilator-Associated Pneumonia in Asia | Human | 88 |
| **Risk factors for carriage of antimicrobial-resistant bacteria in community dwelling children in the Asia-Pacific region: a systematic review and meta-analysis (6)** | Yi Qi Chan | EMR, SEAR, WPR |  | To evaluate the risk factors associated with carriage of AMR bacteria in children. | Human | 25 |
| **Antimicrobial use and resistance data in human and animal sectors in the Lao PDR: evidence to inform policy (7)** | Vilada Chansamouth | WPR | 1994-2020 | What is the evidence on antimicrobial use and resistance in human and animal sectors in Lao | Human; Animal; Environment | 80 |
| **Quantifying drivers of antibiotic resistance in humans: a systematic review  (8)** | Anuja Chatterjee | AMR, EUR, AFR, EMR, SEAR, WPR | 2005-2018 | What are the key drivers of antibiotic resistance in humans | Human; Animal; Environment | 565 |
| **Incidence and Outcomes Associated With Infections Caused by Vancomycin-Resistant Enterococci in the United States: Systematic Literature Review and Meta-Analysis. (9)** | Hsiu-Yin Chiang | AMR | 2000-2015 | Describe the incidence of VRE infections in the US and the clinical and economic outcomes of these infections | Human | 18 |
| **Global contributors to antibiotic resistance (10)** | Aastha Chokshi | AMR, EUR, AFR, EMR, SEAR, WPR | 1963-2017 | What are the various contributors to antibiotic resistance in each type of nation | Human; Animal | 37 |
| **Identifying key influences on antibiotic use in China: a systematic scoping review and narrative synthesis (11)** | Caroline Coope | WPR | 2003-2018 | What are key behaviours, cultural, economic, and social influences on antibiotic use in China | Human | 75 |
| **International travels and transmission of multidrug resistant Neisseria gonorrhoeae in Europe: A systematic review (12)** | Maria de las Mercedes Vicente de la Cruz | EUR | 2010-2021 | To assess the effect of international travel on the transmission of multidrug resistant N Gonorrhoea in Europe. | Human | 18 |
| **The general population's inappropriate behaviors and misunderstanding of antibiotic use in China: A systematic review and meta-analysis (13)** | Lixia Duan | WPR | 2006-2019 | What is the prevalence, and the reasons behind, the general populations irrational use of antibiotics. | Human | 78 |
| **Transmission routes of antibiotic resistant bacteria: a systematic review (14)** | Noortje G. Godijk | AMR, EUR, AFR, EMR, SEAR, WPR | Up to 2019 | What are the routes of acquisition of ARB in humans, animals, water, and the environment? | Human; Animal; Environment | 277 |
| **Antimicrobial Resistance Rates and Surveillance in Sub-Saharan Africa: Where Are We Now? (15)** | Samuel Kariuki | AFR | 2000-2022 | What are the AMR Rates for priority pathogens, and what is being implemented in national action plans to mitigate against AMR | Human; Environment | N/A |
| **Determinants of methicillin-resistant Staphylococcus aureus (MRSA) prevalence in the Asia-Pacific region: A systematic review and meta-analysis (16)** | Wey Wen Lim | SEAR, WPR | 2000-2016 | what is the MRSA prevalence in the Asia Pacific Region? | Human | 229 |
| **The Prevalence, Risk, and Management of Methicillin-Resistant Staphylococcus aureus Infection in Diverse Populations across Canada: A Systematic Review (17)** | Elena Mitevska | AMR | 1991-2017 | To describe the epidemiology of MRSA in Canada from 1991 to 2017, as well as associated risk factors and intervention strategies. | Human | 40 |
| **Antimicrobial resistance among migrants in Europe: a systematic review and meta-analysis (18)** | Laura B Nellums | EUR | 2000-2017 | To identify and synthesise data for AMR carriage or infections, in migrants to Europe, to examine differences in patterns of AMR across migrant groups and in different settings. | Human | 23 |
| **Exploring gender differences in knowledge and practices related to antibiotic use in Southeast Asia: A scoping review. (19)** | Phuc Pham-Duc | SEAR, WPR | 2011-2021 | What is known from the literature about gender differences in antibiotic use in Southeast Asia | Human; Animal; Environment | 13 |
| **Non-biomedical factors affecting antibiotic use in the community: a mixed-methods systematic review and meta-analysis. (20)** | Ruyu Sun | AMR, EUR, AFR, EMR, SEAR, WPR | 2000-2022 | What are the non-biomedical factors influencing healthcare consumers antibiotic use globally? | Human; Animal | 71 |
| **Prevalence of methicillin-resistant Staphylococcus aureus in healthy Chinese population: A system review and meta-analysis (21)** | Man Wu | WPR | 2001-2018 | To determine the prevalence of MRSA in healthy Chinese population, the influencing factors or MRSA colonization, and its antibiotic resistance. | Human; Animal | 37 |
| **Acquisition of extended-spectrum beta-lactamase-producing Enterobacteriaceae (ESBL-PE) carriage after exposure to systemic antimicrobials during travel: Systematic review and meta-analysis (22)** | Terence C. Wuerz | AMR, EUR, AFR, EMR, SEAR, WPR | 2000-2018 | to quantify the extent to which antimicrobial use during travel amplifies the risk of antimicrobial resistances | Human | 15 |
| **Risk factors for quinolone-resistant Escherichia coli infection: A systematic review and meta-analysis (23)** | Dong-Mei Zhu | AMR, EUR, AFR, EMR, SEAR, WPR | Up to 2019 | To evaluate the potential risk factors of QREC | Human | 27 |

Supplementary Table 4: Quality assessment of reviewed articles

| **Quality Indicator** | Yes(2) /Maybe(1) /No (0) |
| --- | --- |
| 1. Did the review address a clearly focused question? (Hint: population studied, intervention given, outcome considered) |  |
| 2. Did the authors look for the right type of papers? (hint: best studies would address the reviews question, have an appropriate study design) |  |
| 3. Do you think all the important, relevant studies were included? (hint: which bibliographic databases were used, follow up from reference lists, unpublished as well as published studies, non-english language studies) |  |
| 4. Did the review’s authors do enough to assess quality of the included studies? (hint: the authors need to consider the rigour of the studies they have identified) |  |
| 5. If the results of the review have been combined, was it reasonable to do so? (hint: consider whether results are similar from study to study, results are clearly displayed, reasons for variations in studies are discussed) |  |
| 6. What are the overall results of the review? (hint: clear about the reviews bottom line results? What are the results? how are the results expressed?) |  |
| 7. How precise are the results? (hint: look at confidence intervals if given) |  |
| 8. Can the results be applied to the local population? (hint: consider whether the patients covered by the review could be sufficiently different to your population to cause concern, your local setting is likely to differ much from that of the review) |  |
| 9. Were all important outcomes considered? (hint: consider whether there is other information you would like to have seen) |  |
| 10. Are the benefits worth the harms and costs? (hint: consider even if it is not addressed by the review, what do you think?) |  |
| **Total score** (<10=low; 10-15=moderate; >15=high) | /20 |

| **Author-Date** | **Reviewer 1** | **Reviewer 2** | **Average** |
| --- | --- | --- | --- |
| 1 Alividza | 14 | 13 | 13.5 |
| 2 Ang | 14 | 15 | 14.5 |
| 3 Bert | 13 | 12 | 12.5 |
| 4 Bokhary | 15 | 14 | 14.5 |
| 5 Bonell | 16 | 14 | 15 |
| 6 Chan | 16 | 15 | 15.5 |
| 7 Chansamouth | 8 | 10 | 9 |
| 8 Chatterjee | 17 | 16 | 16.5 |
| 9 Chiang | 16 | 16 | 16 |
| 10 Chokshi | 7 | 9 | 8 |
| 11 Coope | 13 | 13 | 13 |
| 12 de la Cruz | 13 | 14 | 13.5 |
| 13 Duan | 19 | 18 | 18.5 |
| 14 Godijk | 9 | 12 | 10.5 |
| 15 Kariuki | 8 | 9 | 8.5 |
| 16 Lim | 15 | 13 | 14 |
| 17 Mitevska | 10 | 12 | 11 |
| 18 Nellums | 18 | 17 | 17.5 |
| 19 Pham-Duc | 10 | 9 | 9.5 |
| 20 Sun | 15 | 15 | 15 |
| 21 Wu | 16 | 14 | 15 |
| 22 Wuerz | 18 | 17 | 17.5 |
| 23 Zhu | 17 | 15 | 16 |

Supplementary Table 5: List of excluded articles at full-text review

| **Title** | **Authors** | **Year** | **Reason for exclusion** |
| --- | --- | --- | --- |
| A systematic review of Antimicrobial Stewardship Program implementation in Middle Eastern countries | Ababneh M.A.; Nasser S.A.; Rababa'h A.M. | 2021 | Wrong intervention; |
| Nasal Staphylococcus aureus and S. pseudintermedius carriage in healthy dogs and cats: a systematic review of their antibiotic resistance, virulence and genetic lineages of zoonotic relevance. | Abdullahi, Idris Nasir; Zarazaga, Myriam; Campana-Burguet, Allelen; Eguizabal, Paula; Lozano, Carmen; Torres, Carmen | 2022 | Not human health; |
| Antimicrobial resistance in endodontic infections in Latin America (MICROBE-DENT): Systematic review | Abe F.C.; De Cassia Bergamaschi Motta C.; Filho S.B.; Kodaira K.; De Castro J.P.M.V.; Martins C.C.; Guimaraes C.C.; Lopes L.C. | 2021 | No Socioeconomic Analysis; |
| Prevalence and molecular characteristics of Staphylococcus aureus in raw milk and milk products in Ethiopia: a systematic review and meta-analysis. | Abiot Deddefo; Gezahegne Mamo; Samson Leta; Kebede Amenu | 2022 | Not human health; |
| The Impact of Antimicrobial Stewardship in Children in Low- and Middle-income Countries: A Systematic Review | Abo Y.-N.; Freyne B.; Kululanga D.; Bryant P.A. | 2022 | No drivers/impacts identified; |
| Antimicrobial prescription patterns in East Africa: a systematic review | Acam J.; Kuodi P.; Medhin G.; Makonnen E. | 2023 | No drivers/impacts identified; |
| Antimicrobial Resistance in Nepal. | Acharya, Krishna Prasad; Wilson, R Trevor | 2019 | Narrative Review; |
| Epidemiological Evidence and Health Risks Associated With Agricultural Reuse of Partially Treated and Untreated Wastewater: A Review. | Adegoke, Anthony A; Amoah, Isaac D; Stenstrom, Thor A; Verbyla, Matthew E; Mihelcic, James R | 2018 | Not human health; |
| Global analysis of strategies to tackle antimicrobial resistance. | Adeniji, Funke | 2018 | No drivers/impacts identified; |
| Current trends in the epidemiology and management of enteric fever in Africa: A literature review | Adesegun O.; Adeyemi O.; Ehioghae O.; Rabor D.; Binuyo T.; Alafin B.; Nnagha O.; Idowu A.; Osonuga A. | 2020 | Narrative Review; |
| Surface modifications for antimicrobial effects in the healthcare setting: a critical overview | Adlhart C.; Verran J.; Azevedo N.F.; Olmez H.; Keinanen-Toivola M.M.; Gouveia I.; Melo L.F.; Crijns F. | 2018 | No Socioeconomic Analysis; |
| Interventions to improve dispensing of antibiotics at the community level in low and middle income countries: a systematic review. | Afari-Asiedu, Samuel; Abdulai, Martha Ali; Tostmann, Alma; Boamah-Kaali, Ellen; Asante, Kwaku Poku; Wertheim, Heiman F L; Hulscher, Marlies | 2022 | No drivers/impacts identified; |
| Review of antimicrobial therapy of selected bacterial diseases in broiler chickens in Canada. | Agunos, Agnes; Leger, Dave; Carson, Carolee | 2012 | No Socioeconomic Analysis; |
| Global economic impact of antibiotic resistance: A review. | Ahmad, Mohammad; Khan, Asad U | 2019 | Narrative Review; |
| Strengthening strategic management approaches to address antimicrobial resistance in global human health: a scoping review. | Ahmad, R.; Zhu JiaYue [Zhu, J. Y. N. ]; Leather, A. J. M.; Holmes, A.; Ferlie, E. | 2019 | No quantification done; |
| Long-term antibiotics for prevention of recurrent urinary tract infection in older adults: systematic review and meta-analysis of randomised trials. | Ahmed, Haroon; Davies, Freya; Francis, Nick; Farewell, Daniel; Butler, Christoper; Paranjothy, Shantini | 2017 | No drivers/impacts identified; |
| Containment of antimicrobial resistance due to use of antimicrobial agents in animals intended for food: WHO perspective. | Aidara-Kane, A | 2012 | Narrative Review; |
| Combating Bovine Mastitis in the Dairy Sector in an Era of Antimicrobial Resistance: Ethno-veterinary Medicinal Option as a Viable Alternative Approach. | Ajose, Daniel Jesuwenu; Oluwarinde, Bukola Opeyemi; Abolarinwa, Tesleem Olatunde; Fri, Justine; Montso, Kotsoana Peter; Fayemi, Omolola Esther; Aremu, Adeyemi Oladapo; Ateba, Collins Njie | 2022 | Not human health; |
| A systematic review of biochar use in animal waste composting. | Akdeniz, Neslihan | 2019 | No Socioeconomic Analysis; |
| Antimicrobial stewardship in residential aged care facilities: A systematic review | Akhtar A.; Khan A.H.; Fatima S.; Hassali M.A. | 2020 | No Socioeconomic Analysis; |
| Antimicrobial resistance in Salmonella enterica serovar typhi and paratyphi in South Asia-current status, issues and prospects. | Akhtar, Saeed; Sarker, Mahfuzur R; Jabeen, Kausar; Sattar, Ahsan; Qamar, Aftab; Fasih, Naima | 2015 | Narrative Review; |
| A Review of Quality Measures for Assessing the Impact of Antimicrobial Stewardship Programs in Hospitals. | Akpan, Mary Richard; Ahmad, Raheelah; Shebl, Nada Atef; Ashiru-Oredope, Diane | 2016 | No Socioeconomic Analysis; |
| Implementation of antimicrobial stewardship programmes in African countries: a systematic literature review. | Akpan, Mary Richard; Isemin, Nsisong Udom; Udoh, Arit Esio; Ashiru-Oredope, Diane | 2020 | No Socioeconomic Analysis; |
| Knowledge and practice of antimicrobial usage and resistance among poultry farmers: A systematic review, meta-analysis, and meta-regression. | Al Sattar, Abdullah; Chisty, Nurun Nahar; Irin, Nusrat; Uddin, Md Helal; Hasib, F M Yasir; Hoque, Md Ahasanul | 2023 | Not human health; |
| Quality and utility of information captured by surveillance systems relevant to antimicrobial resistance (AMR): A systematic review | Al-Haboubi M.; Glover R.E.; Eastmure E.; Petticrew M.; Black N.; Mays N. | 2021 | No Socioeconomic Analysis; |
| Antibiotic prescription in Morocco, national data: Meta-analysis | Al-Selwi A.G.M.; Barkat A. | 2022 | No Socioeconomic Analysis; |
| Linking Animal Welfare and Antibiotic Use in Pig Farming-A Review. | Albernaz-Goncalves, Rita; Olmos Antillon, Gabriela; Hotzel, Maria Jose | 2022 | Narrative Review; |
| A systematic review and meta-analysis on antimicrobial resistance in marine bivalves. | Albini, Elisa; Orso, Massimiliano; Cozzolino, Francesco; Sacchini, Luca; Leoni, Francesca; Magistrali, Chiara Francesca | 2022 | Not human health; |
| Opportunities to Improve Awareness of Antimicrobial Resistance Through Social Marketing: A Systematic Review of Interventions Targeting Parents and Children | Alejandro A.L.; Leo W.W.C.; Bruce M. | 2022 | No drivers/impacts identified; |
| A vigilant observation to pregnancy associated listeriosis in Africa: systematic review and meta-analysis. | Alene Geteneh; Sirak Biset; Selamyhun Tadesse; Alemale Admas; Abdu Seid; Demeke Mesfin Belay | 2022 | No Socioeconomic Analysis; |
| Prevalence of beta -lactamases enzymes among Enterobacteriaceae in different Iraqi provinces: A review | Ali Al-Garawyi A.M. | 2019 | Full text not available; |
| Antimicrobial Stewardship Interventions to Optimize Treatment of Infections in Nursing Home Residents: A Systematic Review and Meta-Analysis | Aliyu S.; Travers J.L.; Heimlich S.L.; Ifill J.; Smaldone A. | 2022 | No drivers/impacts identified; |
| Bloodstream infection in long-term care residents: An integrative review of the literature | Aliyu S.E.; Larson E.L. | 2016 | Full text not available; |
| Prevalence of multidrug-resistant gram-negative bacteria among nursing home residents: A systematic review and meta-analysis. | Aliyu, Sainfer; Smaldone, Arlene; Larson, Elaine | 2017 | No quantification done; |
| The prevalence, possible causes and outcomes of self-medication with antibiotics in middle eastern countries | Aljamea Z.; Almahasnah R.; Alkhalifah K.; Basalelah L.; Alhomoud F. | 2017 | No quantification done ; |
| Burden of endemic health-care-associated infection in developing countries: Systematic review and meta-analysis | Allegranzi B.; Nejad S.B.; Combescure C.; Graafmans W.; Attar H.; Donaldson L.; Pittet D. | 2011 | No AMR Analysis; |
| Socioeconomic factors associated with antimicrobial resistance in Latin America: A systematic review and empirical analysis of 41 Chilean hospitals | Allel K.; Garcia P.; Labarca J.; Rendic M.; Munita J.; Undurraga E. | 2020 | Full text not available; |
| Trends in reported antibiotic use among children under 5 years of age with fever, diarrhoea, or cough with fast or difficult breathing across low-income and middle-income countries in 2005-17: a systematic analysis of 132 national surveys from 73 countrie | Allwell-Brown G.; Hussain-Alkhateeb L.; Kitutu F.E.; Stromdahl S.; Martensson A.; Johansson E.W. | 2020 | No drivers/impacts identified; |
| Misuse of antibiotic: A systemic review of Saudi published studies | Alnemri A.R.; Almaghrabi R.H.; Alonazi N.; Alfrayh A.R. | 2016 | No drivers/impacts identified; |
| Prevalence of Bacterial Coinfection and Patterns of Antibiotics Prescribing in Patients with COVID-19: A Systematic review and Meta-Analysis | Alshaikh F.S.; Sindi O.N.; Godman B.; Seaton R.A.; Kurdi A. | 2022 | No drivers/impacts identified; |
| Prevention strategies for methicillin-resistant Staphylococcus aureus (MRSA) in Latin America | Alvarez C.; Labarca J.; Salles M. | 2010 | Narrative Review; |
| Myths and Misconceptions around Antibiotic Resistance: Time to Get Rid of Them. | Am..bile-Cuevas, Carlos F | 2022 | Narrative Review; |
| Antibiotic usage and resistance in Mexico: an update after a decade of change. | Amabile-Cuevas, Carlos F | 2021 | Narrative Review; |
| Isolation, genotyping and antimicrobial resistance of Shiga toxin-producing Escherichia coli. | Amezquita-Lopez, Bianca A; Soto-Beltran, Marcela; Lee, Bertram G; Yambao, Jaszemyn C; Quinones, Beatriz | 2018 | Narrative Review; |
| Methodology for laboratory-based antimicrobial resistance surveillance in animals. | Amin, Md Al; Pasha, Monirul Haque; Hoque, M Nazmul; Siddiki, Amam Zonaed; Saha, Sukumar; Kamal, Md Mostofa | 2022 | No Socioeconomic Analysis; |
| The case for antifungal stewardship. | Ananda-Rajah, Michelle R; Slavin, Monica A; Thursky, Karin T | 2012 | Narrative Review; |
| The economic burden of gonorrhoea in England: Testing, treatment and sequelae | Anderson A.; Christensen H.; Turner K. | 2020 | Full text not available; |
| Interventions to influence consulting and antibiotic use for acute respiratory tract infections in children: a systematic review and meta-analysis. | Andrews, Talley; Thompson, Matthew; Buckley, David I; Heneghan, Carl; Deyo, Rick; Redmond, Niamh; Lucas, Patricia J; Blair, Peter S; Hay, Alastair D | 2012 | No drivers/impacts identified; |
| Fighting antibiotic resistance: A narrative review of public knowledge, attitudes, and perceptions of antibiotics use | Antwi A.N.; Stewart A. | 2019 | Narrative Review; |
| Mobile Tigecycline Resistance: An Emerging Health Catastrophe Requiring Urgent One Health Global Intervention | Anyanwu M.U.; Nwobi O.C.; Okpala C.O.R.; Ezeonu I.M. | 2022 | Narrative Review; |
| Prophylaxis and Treatment against Klebsiella pneumoniae: Current Insights on This Emerging Anti-Microbial Resistant Global Threat. | Arato, Vanessa; Raso, Maria Michelina; Gasperini, Gianmarco; Berlanda Scorza, Francesco; Micoli, Francesca | 2021 | Narrative Review; |
| Role of antimicrobial stewardship programmes in children: a systematic review. | Araujo da Silva, A R; Albernaz de Almeida Dias, D C; Marques, A F; Biscaia di Biase, C; Murni, I K; Dramowski, A; Sharland, M; Huebner, J; Zingg, W | 2018 | No Socioeconomic Analysis; |
| International cooperation to improve access to and sustain effectiveness of antimicrobials. | Ardal, Christine; Outterson, Kevin; Hoffman, Steven J; Ghafur, Abdul; Sharland, Mike; Ranganathan, Nisha; Smith, Richard; Zorzet, Anna; Cohn, Jennifer; Pittet, Didier; Daulaire, Nils; Morel, Chantal; Rizvi, Zain; Balasegaram, Manica; Dar, Osman A; Heymann, David L; Holmes, Alison H; Moore, Luke S P; Laxminarayan, Ramanan; Mendelson, Marc; Rottingen, John-Arne | 2016 | Narrative Review; |
| Azole resistance in Aspergillus: global status in Europe and Asia. | Arikan-Akdagli, Sevtap | 2012 | Narrative Review; |
| Travel and acquisition of multidrug-resistant Enterobacteriaceae. | Armand-Lefevre, L; Andremont, A; Ruppe, E | 2018 | Narrative Review; |
| Prevalence Of carbapenem resistance in Acinetobacter baumanii and Pseudomonas aeruginosa in sub-Saharan Africa: a systematic review and meta-analysis | Arowolo M.T.; Orababa O.Q.; Olaitan M.O.; Osibeluwo B.V.; Essiet U.U.; Batholomew O.H.; Ogunrinde O.G.; Lagoke O.A.; Soriwei J.D.; Ishola O.D.; Ezeani O.M.; Onishile A.O.; Olumodeji E. | 2022 | No drivers/impacts identified; |
| COVID-19 pandemic and antimicrobial resistance in developing countries. | Arshad, Abdul Rehman; Ijaz, Farhat; Siddiqui, Mishal Shan; Khalid, Saad; Fatima, Abeer; Aftab, Rana Khurram | 2021 | Narrative Review; |
| Foodborne Pathogens and Antimicrobial Resistance in Ethiopia: An Urgent Call for Action on "One Health". | Asfaw, Tsegahun; Genetu, Deribew; Shenkute, Demissew; Shenkutie, Tassew Tefera; Amare, Yosef Eshetie; Yitayew, Berhanu | 2022 | No quantification done; |
| Evidence of the Practice of Self-Medication with Antibiotics among the Lay Public in Low- and Middle-Income Countries: A Scoping Review. | Aslam, Adeel; Gajdacs, Mario; Zin, Che Suraya; Ab Rahman, Norny Syafinaz; Ahmed, Syed Imran; Zafar, Muhammad Zeeshan; Jamshed, Shazia | 2020 | No quantification done; |
| Campylobacter at the human-food interface: the african perspective | Asuming-Bediako N.; Kunadu A.P.-H.; Abraham S.; Habib I. | 2019 | Narrative Review; |
| Reducing catheter-associated urinary tract infections: a systematic review of barriers and facilitators and strategic behavioural analysis of interventions. | Atkins, L.; Sallis, A.; Chadborn, T.; Shaw, K.; Schneider, A.; Hopkins, S.; Bunten, A.; Michie, S.; Lorencatto, F. | 2020 | No quantification done; |
| Educational antimicrobial stewardship programs in medical schools: a scoping review. | Augie, Bashar M; Miot, Jacqui; van Zyl, Robyn L; McInerney, Patricia A | 2021 | No drivers/impacts identified; |
| Global access to antibiotics without prescription in community pharmacies: A systematic review and meta-analysis | Auta A.; Hadi M.A.; Oga E.; Adewuyi E.O.; Abdu-Aguye S.N.; Adeloye D.; Strickland-Hodge B.; Morgan D.J. | 2019 | No drivers/impacts identified; |
| Antibiotic resistance in hospital-acquired ESKAPE-E infections in low- and lower-middle-income countries: a systematic review and meta-analysis | Ayobami O.; Brinkwirth S.; Eckmanns T.; Markwart R. | 2022 | No drivers/impacts identified; |
| The incidence and prevalence of hospital-acquired (carbapenem-resistant) Acinetobacter baumannii in Europe, Eastern Mediterranean and Africa: a systematic review and meta-analysis | Ayobami O.; Willrich N.; Harder T.; Okeke I.N.; Eckmanns T.; Markwart R. | 2019 | No drivers/impacts identified; |
| Prevalence and Correlates of Self-Medication Practices for Prevention and Treatment of COVID-19: A Systematic Review. | Ayosanmi, Oluwasola Stephen; Alli, Babatunde Yusuf; Akingbule, Oluwatosin Adetolani; Alaga, Adeyemi Hakeem; Perepelkin, Jason; Marjorie, Delbaere; Sansgiry, Sujit S; Taylor, Jeffrey | 2022 | No AMR Analysis; |
| The threat of antimicrobial resistance in developing countries: causes and control strategies. | Ayukekbong, James A; Ntemgwa, Michel; Atabe, Andrew N | 2017 | Narrative Review; |
| Review of Antimicrobial Resistance in Wastewater in Japan: Current Challenges and Future Perspectives. | Baba, Hiroaki; Nishiyama, Masateru; Watanabe, Toru; Kanamori, Hajime | 2022 | Not human health; |
| Importance of antibiotic residues in animal food. | Bacanli, Merve; Basaran, Nursen | 2019 | No Socioeconomic Analysis; |
| An integrative review of infection prevention and control programs for multidrug-resistant organisms in acute care hospitals: a socio-ecological perspective. | Backman, Chantal; Taylor, Geoffrey; Sales, Anne; Marck, Patricia Beryl | 2011 | No drivers/impacts identified; |
| Prescribing for children - Taste and palatability affect adherence to antibiotics: A review | Baguley D.; Lim E.; Bevan A.; Pallet A.; Faust S.N. | 2012 | Narrative Review; |
| The increasing antimicrobial resistance of Shigella species among Iranian pediatrics: a systematic review and meta-analysis | Baharvand A.; Molaeipour L.; Alesaeidi S.; Shaddel R.; Mashatan N.; Amiriani T.; Kiaei Sudkolaei M.; Abbasian S.; Talib Al-Naqeeb B.Z.; Kouhsari E. | 2023 | No quantification done; |
| Determinants of dispensing antibiotics without prescription in Eritrea: A mixed-method qualitative study on pharmacy professionals' perspective | Bahta M.; Weldemariam D.G.; Tesfamariam S.; Tesfamariam E.H.; Russom M. | 2021 | Wrong study design; |
| Advances in pharmacovigilance initiatives surrounding antimicrobial resistance-Indian perspective | Bairy L.K.; Nayak V.; Avinash A.; Kunder S.K. | 2016 | Narrative Review; |
| Confronting the hidden public health threat: Emerging and re-emerging sexually transmitted infections | Balaji S. | 2021 | Narrative Review; |
| The global burden and epidemiology of invasive non-typhoidal Salmonella infections. | Balasubramanian, Ruchita; Im, Justin; Lee, Jung-Seok; Jeon, Hyon Jin; Mogeni, Ondari D; Kim, Jerome H; Rakotozandrindrainy, Raphael; Baker, Stephen; Marks, Florian | 2019 | Narrative Review; |
| Background changing patterns of neonatal fungal sepsis in a developing country. | Ballot, Daynia E; Bosman, Noma; Nana, Trusha; Ramdin, Tanisha; Cooper, Peter A | 2013 | Wrong study design; |
| Airborne bacteria from wastewater treatment and their antibiotic resistance: a meta-analysis. | Banchon, C. | 2021 | No quantification done; |
| Antimicrobial Resistance in Agri-Food Chain and Companion Animals as a Re-emerging Menace in Post-COVID Epoch: Low-and Middle-Income Countries Perspective and Mitigation Strategies. | Bandyopadhyay, Samiran; Samanta, Indranil | 2020 | Narrative Review; |
| One Health aspects & priority roadmap for fungal diseases : A mini-review. | Banerjee, Sayantan; Denning, David W; Chakrabarti, Arunaloke | 2021 | Narrative Review; |
| Antimicrobial Resistance Following Prolonged Use of Hand Hygiene Products: A Systematic Review. | Banik, Gouri Rani; Durayb, Bandar; King, Catherine; Rashid, Harunor | 2022 | No Socioeconomic Analysis; |
| An Overview of Antibiotics as Emerging Contaminants: Occurrence in Bivalves as Biomonitoring Organisms. | Baralla, Elena; Demontis, Maria P; Dessi, Filomena; Varoni, Maria V | 2021 | Not human health; |
| Gender differences in community-acquired pneumonia | Barbagelata E.; Cilloniz C.; Dominedo C.; Torres A.; Nicolini A.; Solidoro P. | 2020 | Full text not available; |
| Rethinking the benefits and costs of childhood vaccination: The example of the Haemophilus influenzae type b vaccine | Barnighausen T.; Bloom D.E.; Canning D.; Friedman A.; Levine O.S.; O'Brien J.; Privor-Dumm L.; Walker D. | 2011 | Narrative Review; |
| Consumer perceptions of antimicrobial use in animal husbandry: A scoping review. | Barrett, Jaime R; Innes, Gabriel K; Johnson, Kelly A; Lhermie, Guillaume; Ivanek, Renata; Greiner Safi, Amelia; Lansing, David | 2021 | Not human health; |
| Urbanization and Waterborne Pathogen Emergence in Low-Income Countries: Where and How to Conduct Surveys?. | Bastaraud, Alexandra; Cecchi, Philippe; Handschumacher, Pascal; Altmann, Mathias; Jambou, Ronan | 2020 | Narrative Review; |
| Antibiotic Dispensation without a Prescription Worldwide: A Systematic Review. | Batista, Ana Daniela; A Rodrigues, Daniela; Figueiras, Adolfo; Zapata-Cachafeiro, Maruxa; Roque, Fatima; Herdeiro, Maria Teresa | 2020 | No AMR Analysis; |
| Reducing Antimicrobial Use and Dependence in Livestock Production Systems: A Social and Economic Sciences Perspective on an Interdisciplinary Approach. | Baudoin, Fanny; Hogeveen, Henk; Wauters, Erwin | 2021 | Narrative Review; |
| The challenges of patient satisfaction: influencing factors and the patient-provider relationship in the United States | Baummer - Carr A.; Nicolau D.P. | 2017 | Narrative Review; |
| Salmonella in Swine: Prevalence, Multidrug Resistance, and Vaccination Strategies. | Bearson, Shawn M D | 2022 | No drivers/impacts identified; |
| Methicillin resistance in Staphylococcus isolates: the "mec alphabet" with specific consideration of mecC, a mec homolog associated with zoonotic S. aureus lineages. | Becker, Karsten; Ballhausen, Britta; Kock, Robin; Kriegeskorte, Andre | 2014 | Narrative Review; |
| Non-prescription dispensing of antibiotic agents among community drug retail outlets in Sub-Saharan African countries: a systematic review and meta-analysis | Belachew S.A.; Hall L.; Selvey L.A. | 2021 | No drivers/impacts identified; |
| No prescription? No problem: drivers of non-prescribed sale of antibiotics among community drug retail outlets in low and middle income countries: a systematic review of qualitative studies. | Belachew, S. A.; Hall, L.; Erku, D. A.; Selvey, L. A. | 2021 | No quantification done; |
| Antimicrobial stewardship programmes in nursing homes: A systematic review and inventory of tools | Belan M.; Thilly N.; Pulcini C. | 2020 | No drivers/impacts identified; |
| Escherichia coli from animal reservoirs as a potential source of human extraintestinal pathogenic E. coli. | Belanger, Louise; Garenaux, Amelie; Harel, Josee; Boulianne, Martine; Nadeau, Eric; Dozois, Charles M | 2011 | Narrative Review; |
| A systematic review on drug resistant urinary tract infection among pregnant women in developing countries in africa and asia; 2005-2016 | Belete M.A.; Saravanan M. | 2020 | No drivers/impacts identified; |
| Methicillin-Resistant Staphylococcus aureus (MRSA) and Other Methicillin-Resistant Staphylococci and Mammaliicoccus (MRNaS) Associated with Animals and Food Products in Arab Countries: A Review. | Belhout, Chahrazed; Elgroud, Rachid; Butaye, Patrick | 2022 | Narrative Review; |
| An analysis of the ideational, behavioral and structural determinants of antimicrobial resistance: A global review of the literature | Benie W.; Dosso A.; Dadie T.; Dali S.; Tibbels N.; Brou J.; Yao A.; McKay M.; Fordham C.; Nguessan N.; Koko R.; Kamara D.; Naugle D. | 2020 | Wrong study design; |
| Self-medication: A current challenge | Bennadi D. | 2014 | No quantification done; |
| Antimicrobial resistance in West Africa: a systematic review and meta-analysis. | Bernabe, Kerlly J; Langendorf, Celine; Ford, Nathan; Ronat, Jean-Baptiste; Murphy, Richard A | 2017 | No drivers/impacts identified; |
| Increasing Globalization and the Movement of Antimicrobial Resistance between Countries. | Berndtson, Allison E | 2020 | Narrative Review; |
| Prevalence and drug resistance patterns of Gram-negative enteric bacterial pathogens from diarrheic patients in Ethiopia: A systematic review and meta-analysis | Beyene A.M.; Gezachew M.; Mengesha D.; Yousef A.; Gelaw B. | 2022 | No drivers/impacts identified; |
| Bacterial zoonoses transmitted by household pets and as reservoirs of antimicrobial resistant bacteria. | Bhat, Aashaq Hussain | 2021 | Narrative Review; |
| 375 The association between antibiotics for acne and subsequent infection sequelae and antimicrobial resistance: A systematic review | Bhate K.; Lin L.; Barbieri J.; Mathur R.; Sinnott S.; Langan S. | 2020 | No Socioeconomic Analysis; |
| Global burden, distribution, and interventions for infectious diseases of poverty. | Bhutta, Z. A.; Sommerfeld, J.; Lassi, Z. S.; Salam, R. A.; Das, J. K. | 2014 | Narrative Review; |
| Measures to eradicate multidrug-resistant organism outbreaks: how much do they cost?. | Birgand, G; Moore, L S P; Bourigault, C; Vella, V; Lepelletier, D; Holmes, A H; Lucet, J-C | 2016 | No drivers/impacts identified; |
| Antibiotic geographies and access to medicines: Tracing the role of India's pharmaceutical industry in global trade | Bjerke L. | 2022 | No drivers/impacts identified; |
| Infectious disease threats in the twenty-first century: Strengthening the global response | Bloom D.E.; Cadarette D. | 2019 | Narrative Review; |
| Risk factors for antimicrobial use in food-producing animals: Disease prevention and socio-economic factors as the main drivers? | Bokma J.; Dewulf J.; Deprez P.; Pardon B. | 2018 | Not human health; |
| Antimicrobial resistance among Enterobacteriaceae in South America: history, current dissemination status and associated socioeconomic factors. | Bonelli, Raquel Regina; Moreira, Beatriz Meurer; Picao, Renata Cristina | 2014 | Narrative Review; |
| A systematic review to explore influences on parental attitudes towards antibiotic prescribing in children. | Bosley, Helen; Henshall, Catherine; Appleton, Jane V; Jackson, Debra | 2018 | No quantification done; |
| The impact of Antimicrobial Stewardship Programmes in paediatric emergency departments and primary care: a systematic review. | Brigadoi, Giulia; Rossin, Sara; Visentin, Davide; Barbieri, Elisa; Giaquinto, Carlo; Da Dalt, Liviana; Dona, Daniele | 2023 | No drivers/impacts identified; |
| Antimicrobial growth promoter use in livestock: a requirement to understand their modes of action to develop effective alternatives. | Brown, Kirsty; Uwiera, Richard R E; Kalmokoff, Martin L; Brooks, Steve P J; Inglis, G Douglas | 2017 | Narrative Review; |
| Drug-resistant enteric fever worldwide, 1990 to 2018: A systematic review and meta-analysis | Browne A.J.; Kashef Hamadani B.H.; Kumaran E.A.P.; Rao P.; Longbottom J.; Harriss E.; Moore C.E.; Dunachie S.; Basnyat B.; Baker S.; Lopez A.D.; Day N.P.J.; Hay S.I.; Dolecek C. | 2020 | No drivers/impacts identified; |
| Epidemiology of Helicobacter pylori infection among peoples of the Arctic | Bruce M.; Miernyk K.; Goodman K.; Koch A.; Hansen H.; Tsukanov V.; McMahon B. | 2018 | Full text not available; |
| Surveillance of infectious diseases in the Arctic. | Bruce, M; Zulz, T; Koch, A | 2016 | Narrative Review; |
| Identifying hotspots for antibiotic resistance emergence and selection, and elucidating pathways to human exposure: Application of a systems-thinking approach to aquaculture systems | Brunton L.A.; Desbois A.P.; Garza M.; Wieland B.; Mohan C.V.; Hasler B.; Tam C.C.; Le P.N.T.; Phuong N.T.; Van P.T.; Nguyen-Viet H.; Eltholth M.M.; Pham D.K.; Duc P.P.; Linh N.T.; Rich K.M.; Mateus A.L.P.; Hoque M.A.; Ahad A.; Khan M.N.A.; Adams A.; Guitian J. | 2019 | Wrong study design; |
| Consumption of penicillins in the community, European Union/European Economic Area, 1997-2017 | Bruyndonckx R.; Adriaenssens N.; Hens N.; Versporten A.; Monnet D.L.; Molenberghs G.; Goossens H.; Weist K.; Coenen S. | 2021 | Wrong study design; |
| Faecal carriage of antibiotic resistant Escherichia coli in asymptomatic children and associations with primary care antibiotic prescribing: A systematic review and meta-analysis | Bryce A.; Costelloe C.; Hawcroft C.; Wootton M.; Hay A.D. | 2016 | No drivers/impacts identified; |
| Global prevalence of antibiotic resistance in paediatric urinary tract infections caused by Escherichia coli and association with routine use of antibiotics in primary care: systematic review and meta-analysis. | Bryce, Ashley; Hay, Alastair D; Lane, Isabel F; Thornton, Hannah V; Wootton, Mandy; Costelloe, Ceire | 2016 | No Socioeconomic Analysis; |
| Identifying global research gaps to mitigate antimicrobial resistance: A scoping review | Bulteel A.J.; Larson E.L.; Getahun H. | 2020 | No drivers/impacts identified; |
| Communication interventions to promote the public's awareness of antibiotics: a systematic review. | Burstein, V. R.; Trajano, R. P.; Kravitz, R. L.; Bell, R. A.; Vora, D.; May, L. S. | 2019 | No drivers/impacts identified; |
| Infectious disease emergence and global change: Thinking systemically in a shrinking world | Butler C.D. | 2012 | No AMR Analysis; |
| The drivers of antibiotic use and misuse: the development and investigation of a theory driven community measure | Byrne M.K.; Miellet S.; McGlinn A.; Fish J.; Meedya S.; Reynolds N.; van Oijen A.M. | 2019 | Wrong study design; |
| PIN92 DO INTERNATIONAL MIGRANTS FACE HIGHER BACTERIAL DRUG RESISTANCE COMPARED TO NATIVES? A SYSTEMATIC REVIEW | Cabieses B.; Peters A.; Acuna M.P.; Uphoff N.; Astorga S.; Munita J.M. | 2019 | Full text not available; |
| Prevalence of healthcare-associated infections and antimicrobial use among adult inpatients in Singapore acute-care hospitals: Results from the first national point prevalence survey | Cai Y.; Venkatachalam I.; Tee N.W.; Yen Tan T.; Kurup A.; Wong S.Y.; Low C.Y.; Wang Y.; Lee W.; Liew Y.X.; Ang B.; Lye D.C.; Chow A.; Ling M.L.; Oh H.M.; Cuvin C.A.; Ooi S.T.; Pada S.K.; Lim C.H.; Tan J.W.C.; Chew K.L.; Nguyen V.H.; Fisher D.A.; Goossens H.; Kwa A.L.; Tambyah P.A.; Hsu L.Y.; Marimuthu K. | 2017 | Wrong study design; |
| Clinical and economic impact of bacterial resistance: an approach to infection control and antimicrobial stewardship solutions. | Calbo, Esther; Boix-Palop, Lucia; Garau, Javier | 2020 | Narrative Review; |
| Tackling AMR from a multidisciplinary perspective: a primer from education and psychology. | Calvo-Villamanan, Alicia; San Millan, Alvaro; Carrilero, Laura | 2023 | Narrative Review; |
| To Push or to Pull? in a Post-COVID World, Supporting and Incentivizing Antimicrobial Drug Development Must Become a Governmental Priority | Cama J.; Leszczynski R.; Tang P.K.; Khalid A.; Lok V.; Dowson C.G.; Ebata A. | 2021 | Narrative Review; |
| The problem of Helicobacter pylori resistance to antibiotics: a systematic review in Latin America. | Camargo, M Constanza; Garcia, Apolinaria; Riquelme, Arnoldo; Otero, William; Camargo, Claudia A; Hernandez-Garcia, Tomas; Candia, Roberto; Bruce, Michael G; Rabkin, Charles S | 2014 | No quantification done; |
| In critique of anthropocentrism: a more-than-human ethical framework for antimicrobial resistance. | Canada, Jose A; Sariola, Salla; Butcher, Andrea | 2022 | Narrative Review; |
| Parental knowledge of antibiotic use in children with respiratory infections: a systematic review. | Cantarero-Arevalo, Lourdes; Hallas, Mia Pavelics; Kaae, Susanne | 2017 | No quantification done; |
| Antibiotic-resistant bacteria: a challenge for the food industry. | Capita, Rosa; Alonso-Calleja, Carlos | 2013 | Narrative Review; |
| Fasciolosis in South America: Epidemiology and control challenges | Carmona C.; Tort J.F. | 2017 | Narrative Review; |
| The global need for effective antibiotics-moving towards concerted action. | Cars, Otto; Hedin, Anna; Heddini, Andreas | 2011 | Narrative Review; |
| Attributable deaths and disability-adjusted life-years caused by infections with antibiotic-resistant bacteria in the EU and the European Economic Area in 2015: a population-level modelling analysis | Cassini A.; Hogberg L.D.; Plachouras D.; Quattrocchi A.; Hoxha A.; Simonsen G.S.; Colomb-Cotinat M.; Kretzschmar M.E.; Devleesschauwer B.; Cecchini M.; Ouakrim D.A.; Oliveira T.C.; Struelens M.J.; Suetens C.; Monnet D.L.; Strauss R.; Mertens K.; Struyf T.; Catry B.; Latour K.; Ivanov I.N.; Dobreva E.G.; Tambic Andrasevic A.; Soprek S.; Budimir A.; Paphitou N.; Zemlickova H.; Schytte Olsen S.; Wolff Sonksen U.; Martin P.; Ivanova M.; Lyytikainen O.; Jalava J.; Coignard B.; Eckmanns T.; Abu Sin M.; Haller S.; Daikos G.L.; Gikas A.; Tsiodras S.; Kontopidou F.; Toth A.; Hajdu A.; Guolaugsson O.; Kristinsson K.G.; Murchan S.; Burns K.; Pezzotti P.; Gagliotti C.; Dumpis U.; Liuimiene A.; Perrin M.; Borg M.A.; de Greeff S.C.; Monen J.C.; Koek M.B.; Elstrom P.; Zabicka D.; Deptula A.; Hryniewicz W.; Canica M.; Nogueira P.J.; Fernandes P.A.; Manageiro V.; Popescu G.A.; Serban R.I.; Schreterova E.; Litvova S.; Stefkovicova M.; Kolman J.; Klavs I.; Korosec A.; Aracil B.; Asensio A.; Perez-Vazquez M.; Billstrom H.; Larsson S.; Reilly J.S.; Johnson A.; Hopkins S. | 2019 | Wrong study design; |
| Antibiotic susceptibility among non-clinical Escherichia coli as a marker of antibiotic pressure in Peru (2009-2019): one health approach. | Castillo, Angie K; Espinoza, Kathya; Chaves, Antony F; Guibert, Fernando; Ruiz, Joaquim; Pons, Maria J | 2022 | Not human health; |
| What we know about media communication on antibiotics and antimicrobial resistance: A systematic review of the scientific literature | Catalan-Matamoros D.; Pariente A.; Elias-Perez C. | 2019 | No quantification done; |
| HTA9 The Benefits That a Novel Antimicrobial Provides When Viewed From an Insurance Value Perspective | Chan M.S.; Holloway R.; King R.; Polya R.; Sloan R.; Kowalik J.C.; Ashfield T.; Moore L.S.; Porter T.; Pearson-Stuttard J. | 2022 | Full text not available; |
| Antimicrobial resistance in cities: an overlooked challenge that requires a multidisciplinary approach | Chandler, Clare I. R.; Nayiga, Susan | 2023 | Wrong study design; |
| Antimicrobial resistance and the post antibiotic era: better late than never effort. | Chandra, Prashant; Mk, Unnikrishnan; Ke, Vandana; Mukhopadhyay, Chiranjay; U, Dinesh Acharya; M, Surulivel Rajan; V, Rajesh | 2021 | Narrative Review; |
| Prevalence and pattern of antimicrobial use in Indian setting: An evidence from systematic review and meta-analysis | Chandrasekhar B.; Vidyasagar K.; Chhabra M.; Bansal D. | 2020 | Full text not available; |
| Opportunities for system level improvement in antibiotic use across the surgical pathway. | Charani, E; Ahmad, R; Tarrant, C; Birgand, G; Leather, A; Mendelson, M; Moonesinghe, S R; Sevdalis, N; Singh, S; Holmes, A | 2017 | Narrative Review; |
| The role of behavior change in antimicrobial stewardship. | Charani, Esmita; Castro-Sanchez, Enrique; Holmes, Alison | 2014 | Narrative Review; |
| Antibiotic Stewardship-Twenty Years in the Making. | Charani, Esmita; Holmes, Alison | 2019 | Narrative Review; |
| Navigating sociocultural disparities in relation to infection and antibiotic resistance-the need for an intersectional approach. | Charani, Esmita; Mendelson, Marc; Ashiru-Oredope, Diane; Hutchinson, Eleanor; Kaur, Manmeet; McKee, Martin; Mpundu, Mirfin; Price, James R; Shafiq, Nusrat; Holmes, Alison | 2021 | Narrative Review; |
| An analysis of existing national action plans for antimicrobial resistance-gaps and opportunities in strategies optimising antibiotic use in human populations. | Charani, Esmita; Mendelson, Marc; Pallett, Scott J C; Ahmad, Raheelah; Mpundu, Mirfin; Mbamalu, Oluchi; Bonaconsa, Candice; Nampoothiri, Vrinda; Singh, Sanjeev; Peiffer-Smadja, Nathan; Anton-Vazquez, Vanesa; Moore, Luke S P; Schouten, Jeroen; Kostyanev, Tomislav; Vlahovic-Palcevski, Vera; Kofteridis, Diamantis; Correa, Juliana Silva; Holmes, Alison H | 2023 | No quantification done; |
| Investigating the cultural and contextual determinants of antimicrobial stewardship programmes across low-, middle- and high-income countries-A qualitative study | Charani, Esmita; Smith, Ingrid; Skodvin, Brita; Perozziello, Anne; Lucet, Jean-Christophe; Lescure, Fran√ßois-Xavier; Birgand, Gabriel; Poda, Armel; Ahmad, Raheelah; Singh, Sanjeev; Holmes, Alison Helen | 2019 | No quantification done; |
| Influences on antibiotic prescribing by non-medical prescribers for respiratory tract infections: a systematic review using the theoretical domains framework | Chater A.; Family H.; Lim R.; Courtenay M. | 2020 | No quantification done; |
| Mapping cholera outbreaks and antibiotic resistant Vibrio cholerae in India: An assessment of existing data and a scoping review of the literature | Chatterjee P.; Kanungo S.; Bhattacharya S.K.; Dutta S. | 2020 | No drivers/impacts identified; |
| Improved understanding of factors driving methicillin-resistant Staphylococcus aureus epidemic waves | Chatterjee S.S.; Otto M. | 2013 | Narrative Review; |
| Quantifying drivers of antibiotic resistance in humans: a systematic review. | Chatterjee, A.; Modarai, M.; Naylor, N. R.; Boyd, S. E.; Atun, R.; Barlow, J.; Holmes, A. H.; Johnson, A.; Robotham, J. V. | 2018 | No quantification done; |
| Role of antimicrobial restrictions in bacterial resistance control: a systematic literature review | Chatzopoulou M.; Reynolds L. | 2020 | No drivers/impacts identified; |
| The knowledge, attitude and practice of health practitioners towards antibiotic prescribing and resistance in developing countries-A systematic review. | Chaw, P S; Hopner, J; Mikolajczyk, R | 2018 | No drivers/impacts identified; |
| Economic burden of antibiotic-resistant nosocomial gram-negative infections in Singapore hospitals | Chen G.; Lim S.; Ma Q.; Ghosh W. | 2016 | Full text not available; |
| Risk factors for antibiotic resistance development in healthcare settings in China: A systematic review | Chen Q.; Li D.; Beiersmann C.; Neuhann F.; Moazen B.; Lu G.; Muller O. | 2021 | No drivers/impacts identified; |
| Prevalence of subclinical mastitis among dairy cattle and associated risks factors in China during 2012-2021: A systematic review and meta-analysis | Chen X.; Chen Y.; Zhang W.; Chen S.; Wen X.; Ran X.; Wang H.; Zhao J.; Qi Y.; Xue N. | 2022 | Not human health; |
| New epidemiology of Staphylococcus aureus infection in Asia. | Chen, C-J; Huang, Y-C | 2014 | No drivers/impacts identified; |
| Antibiotics and Food Safety in Aquaculture. | Chen, Jiemin; Sun, Runxia; Pan, Changgui; Sun, Yue; Mai, Bixian; Li, Qing X | 2020 | Narrative Review; |
| Antibiotics versus placebo for acute bacterial conjunctivitis. | Chen, Yu-Yen; Liu, Su-Hsun; Nurmatov, Ulugbek; van Schayck, Onno Cp; Kuo, Irene C | 2023 | No AMR Analysis; |
| Selection and dissemination of antimicrobial resistance in Agri-food production. | Cheng, Guyue; Ning, Jianan; Ahmed, Saeed; Huang, Junhong; Ullah, Rizwan; An, Boyu; Hao, Haihong; Dai, Menghong; Huang, Lingli; Wang, Xu; Yuan, Zonghui | 2019 | Narrative Review; |
| Bacterial antibiotic resistance development and mutagenesis following exposure to subinhibitory concentrations of fluoroquinolones in vitro: a systematic review of the literature. | Ching, Carly; Orubu, Ebiowei S F; Sutradhar, Indorica; Wirtz, Veronika J; Boucher, Helen W; Zaman, Muhammad H | 2020 | No Socioeconomic Analysis; |
| The prevalence and antimicrobial resistance phenotypes of Salmonella, Escherichia coli, and Enterococcus sp. in surface water | Cho S.; Jackson C.R.; Frye J.G. | 2020 | Narrative Review; |
| Current state of antimicrobial stewardship and organ transplantation in Thailand. | Chotiprasitsakul, Darunee; Bruminhent, Jackrapong; Watcharananan, Siriorn P | 2022 | No drivers/impacts identified; |
| An analysis of national action plans on antimicrobial resistance in Southeast Asia using a governance framework approach. | Chua, Alvin Qijia; Verma, Monica; Hsu, Li Yang; Legido-Quigley, Helena | 2021 | No drivers/impacts identified; |
| Helicobacter pylori (H. Pylori) eradication rates and antibiotic resistance pattern in Nigeria, West Africa: A systematic review | Chukwudike E.S.; Asombang A.W.; Sawyer K.; Tazinkeng N.N.; Oyeleke G.K.; Egboh S.-M.C.; Nowbuth A.A.; Bitto T.T.; Moyo C.M.; Moss S. | 2021 | Full text not available; |
| Mobile apps for detecting falsified and substandard drugs: A systematic review | Ciapponi A.; Donato M.; Metin Gulmezoglu A.; Alconada T.; Bardach A. | 2021 | No drivers/impacts identified; |
| Mycoplasma genitalium incidence, persistence, concordance between partners and progression: Systematic review and meta-analysis | Cina M.; Baumann L.; Egli-Gany D.; Halbeisen F.S.; Ali H.; Scott P.; Low N. | 2019 | No AMR Analysis; |
| Emerging infectious diseases in southeast Asia: regional challenges to control. | Coker, Richard J; Hunter, Benjamin M; Rudge, James W; Liverani, Marco; Hanvoravongchai, Piya | 2011 | No quantification done; |
| Anthropological and socioeconomic factors contributing to global antimicrobial resistance: a univariate and multivariable analysis | Collignon, Peter; Beggs, John J.; Walsh, Timothy R.; Gandra, Sumanth; Laxminarayan, Ramanan | 2018 | Wrong study design; |
| Antibiotic Use and Bacterial Infection in COVID-19 Patients in the Second Phase of the SARS-CoV-2 Pandemic: A Scoping Review | Cong W.; Stuart B.; AIhusein N.; Liu B.; Tang Y.; Wang H.; Wang Y.; Manchundiya A.; Lambert H. | 2022 | No drivers/impacts identified; |
| Worldwide comparison of treatment guidelines for sore throat | Coutinho G.; Duerden M.; Sessa A.; Caretta-Barradas S.; Altiner A. | 2021 | No quantification done; |
| Antibiotic stewardship in low- and middle-income countries: the same but different?. | Cox, J A; Vlieghe, E; Mendelson, M; Wertheim, H; Ndegwa, L; Villegas, M V; Gould, I; Levy Hara, G | 2017 | Narrative Review; |
| Interventions to facilitate shared decision making to address antibiotic use for acute respiratory infections in primary care. | Coxeter, Peter; Del Mar, Chris B; McGregor, Leanne; Beller, Elaine M; Hoffmann, Tammy C | 2015 | No Socioeconomic Analysis; |
| Behavior-change interventions to improve antimicrobial stewardship in human health, animal health, and livestock agriculture: A systematic review | Craig J.; Sadoff R.; Bennett S.; Bahati F.; Beauvais W. | 2023 | No drivers/impacts identified; |
| Interventions to improve appropriate antibiotic prescribing in long-term care facilities: a systematic review | Crayton E.; Richardson M.; Fuller C.; Smith C.; Liu S.; Forbes G.; Anderson N.; Shallcross L.; Michie S.; Hayward A.; Lorencatto F. | 2020 | No drivers/impacts identified; |
| Approaches to promoting the appropriate use of antibiotics through hospital electronic prescribing systems: a scoping review | Cresswell K.; Mozaffar H.; Shah S.; Sheikh A. | 2017 | No drivers/impacts identified; |
| Systematic review of public-targeted communication interventions to improve antibiotic use. | Cross, Elizabeth Louise Anne; Tolfree, Robert; Kipping, Ruth | 2017 | No drivers/impacts identified; |
| Next-generation Sequencing for Surveillance of Antimicrobial Resistance and Pathogenicity in Municipal Wastewater Treatment Plants | Cuetero-Martinez Y.; de los Cobos-Vasconcelos D.; Aguirre-Garrido J.F.; Lopez-Vidal Y.; Noyola A. | 2023 | Full text not available; |
| Improving antibiotic use through behaviour change: a systematic review of interventions evaluated in low- and middle-income countries. | Cuevas, C.; Batura, N.; Wulandari, L. P. L.; Mishal Khan; Wiseman, V. | 2021 | No drivers/impacts identified; |
| Use of and microbial resistance to antibiotics in China: a path to reducing antimicrobial resistance. | Cui, Dan; Liu, Xinliang; Hawkey, Peter; Li, Hao; Wang, Quan; Mao, Zongfu; Sun, Jing | 2017 | Narrative Review; |
| The Pharmacoeconomic Aspects of Antibiotic Stewardship Programs | Cunha C.B. | 2018 | Narrative Review; |
| Antimicrobial Usage in Animal Production: A Review of the Literature with a Focus on Low- and Middle-Income Countries. | Cuong, Nguyen V; Padungtod, Pawin; Thwaites, Guy; Carrique-Mas, Juan J | 2018 | Not human health; |
| Understanding the patient experience of health care-associated infection: A qualitative systematic review | Currie K.; Melone L.; Stewart S.; King C.; Holopainen A.; Clark A.M.; Reilly J. | 2018 | No drivers/impacts identified; |
| The effectiveness of computerised decision support on antibiotic use in hospitals: A systematic review | Curtis C.E.; Al Bahar F.; Marriott J.F. | 2017 | No drivers/impacts identified; |
| Public knowledge, attitudes, and behaviors around antibiotic use in self-limiting infections: A scoping review | Cutajar E.; Currie K.; Flowers P.; Dickson A. | 2019 | Full text not available; |
| ECONOMIC EVALUATIONS OF POINT-OF-CARE INTERVENTIONS TO TACKLE INAPPROPRIATE PRESCRIBING AND ANTIMICROBIAL RESISTANCE IN HIGH- AND MIDDLE-INCOME COUNTRIES: A SYSTEMATIC REVIEW | D'Hulster E.; De Burghgraeve T.; Luyten J.; Verbakel J. | 2022 | Full text not available; |
| Cost-effectiveness of point-of-care interventions to tackle inappropriate prescribing of antibiotics in high- and middle-income countries: a systematic review | D'hulster E.; De Burghgraeve T.; Luyten J.; Verbakel J.Y. | 2023 | No drivers/impacts identified; |
| Antimicrobial Resistance: Implications and Costs. | Dadgostar, Porooshat | 2019 | No drivers/impacts identified; |
| Acinetobacter spp in a Third World Country with Socio-economic and Immigrants Challenges. | Dandachi, Iman; Azar, Eid; Hamouch, Ramzi; Maliha, Peter; Abdallah, Samah; Kanaan, Elie; Badawi, Rebecca; Khairallah, Tamara; Matar, Ghassan M; Daoud, Ziad | 2019 | Narrative Review; |
| Public Health Risks of Multiple-Drug-Resistant Enterococcus spp. in Southeast Asia. | Daniel, Diane Sunira; Lee, Sui Mae; Dykes, Gary A; Rahman, Sadequr | 2015 | Narrative Review; |
| Epidemiological Investigations of Infectious Diseases among Mobile Populations at the University Hospital Institute Mediterranean Infection in Marseille, France. | Dao, Thi Loi; Hoang, Van Thuan; Ly, Tran Duc Anh; Goumballa, Ndiaw; Gautret, Philippe | 2021 | Wrong study design; |
| Antibiotic prescribing practices by dentists: A review | Dar-Odeh N.S.; Abu-Hammad O.A.; Al-Omiri M.K.; Khraisat A.S.; Shehabi A.A. | 2010 | No Socioeconomic Analysis; |
| Exploring the evidence base for national and regional policy interventions to combat resistance. | Dar, Osman A; Hasan, Rumina; Schlundt, Jorgen; Harbarth, Stephan; Caleo, Grazia; Dar, Fazal K; Littmann, Jasper; Rweyemamu, Mark; Buckley, Emmeline J; Shahid, Mohammed; Kock, Richard; Li, Henry Lishi; Giha, Haydar; Khan, Mishal; So, Anthony D; Bindayna, Khalid M; Kessel, Anthony; Pedersen, Hanne Bak; Permanand, Govin; Zumla, Alimuddin; Rottingen, John-Arne; Heymann, David L | 2016 | No AMR Analysis; |
| The crisis of carbapenemase-mediated carbapenem resistance across the human-animal-environmental interface in India | Das S. | 2023 | Narrative Review; |
| Artificial Intelligence and Antibiotic Discovery. | David, Liliana; Brata, Anca Monica; Mogosan, Cristina; Pop, Cristina; Czako, Zoltan; Muresan, Lucian; Ismaiel, Abdulrahman; Dumitrascu, Dinu Iuliu; Leucuta, Daniel Corneliu; Stanculete, Mihaela Fadygas; Iaru, Irina; Popa, Stefan Lucian | 2021 | No drivers/impacts identified; |
| Raw diets for dogs and cats: a review, with particular reference to microbiological hazards. | Davies, R H; Lawes, J R; Wales, A D | 2019 | Narrative Review; |
| Prevalence of metallo-beta-lactamases as a correlate of multidrug resistance among clinical Pseudomonas aeruginosa isolates in Nepal | Dawadi P.; Khadka C.; Shyaula M.; Syangtan G.; Joshi T.P.; Pepper S.H.; Kanel S.R.; Pokhrel L.R. | 2022 | No drivers/impacts identified; |
| Antimicrobial resistance in the globalized food chain: a One Health perspective applied to the poultry industry. | de Mesquita Souza Saraiva, Mauro; Lim, Kelvin; do Monte, Daniel Farias Marinho; Givisiez, Patricia Emilia Naves; Alves, Lucas Bocchini Rodrigues; de Freitas Neto, Oliveiro Caetano; Kariuki, Samuel; Junior, Angelo Berchieri; de Oliveira, Celso Jose Bruno; Gebreyes, Wondwossen Abebe | 2022 | Narrative Review; |
| Epidemiology of otitis media in children from developing countries: A systematic review | DeAntonio R.; Yarzabal J.-P.; Cruz J.P.; Schmidt J.E.; Kleijnen J. | 2016 | No drivers/impacts identified; |
| A systematic literature review of the economic implications of acute bacterial skin and skin structure infections (ABSSSIs) | Degener F.; Ivanescu C.; Casamayor M.; Postma M. | 2015 | Full text not available; |
| An update on new antibiotic prophylaxis and treatment for urinary tract infections in children. | Delbet, Jean Daniel; Lorrot, Mathie; Ulinski, Tim | 2017 | Narrative Review; |
| How are large-scale One Health initiatives targeting infectious diseases and antimicrobial resistance evaluated? A scoping review | Delesalle L.; Sadoine M.L.; Mediouni S.; Denis-Robichaud J.; Zinszer K.; Zarowsky C.; Aenishaenslin C.; Carabin H. | 2022 | No quantification done; |
| European campaigns for a prudent use of antibiotics-a literature review | Denkel L.A.; Zweigner J.; Gensichen J.; Gastmeier P. | 2016 | Full text not available; |
| Antibiotic resistance (ABR) and community pharmacist: A review | Denny D.C.; Karan S. | 2021 | Narrative Review; |
| Microbial Resistance Movements: An Overview of Global Public Health Threats Posed by Antimicrobial Resistance, and How Best to Counter. | Dhingra, Sameer; Rahman, Nor Azlina A; Peile, Ed; Rahman, Motiur; Sartelli, Massimo; Hassali, Mohamed Azmi; Islam, Tariqul; Islam, Salequl; Haque, Mainul | 2020 | Narrative Review; |
| Prevention Strategies to Combat Antimicrobial Resistance in Children in Resource-Limited Settings | Diaz A.; Antonara S.; Barton T. | 2018 | Narrative Review; |
| Economic Evaluation of FebriDx: A Novel Rapid, Point-of-Care Test for Differentiation of Viral versus Bacterial Acute Respiratory Infection in the United States | Dick K.; Schneider J. | 2021 | No Socioeconomic Analysis; |
| Measuring the impact of antimicrobial stewardship programs. | Dik, Jan-Willem H; Hendrix, Ron; Poelman, Randy; Niesters, Hubert G; Postma, Maarten J; Sinha, Bhanu; Friedrich, Alexander W | 2016 | Narrative Review; |
| Systematic review and meta-analyses of incidence for group B streptococcus disease in infants and antimicrobial resistance, China | Ding Y.; Wang Y.; Hsia Y.; Russell N.; Heath P.T. | 2020 | No Socioeconomic Analysis; |
| Psychological and cultural factors influencing antibiotic prescription. | Dionisio, Francisco; Baquero, Fernando; Fuertes, Marina | 2023 | Narrative Review; |
| A review of antibiotic prophylaxis for traveler's diarrhea: Past to present | Diptyanusa A.; Ngamprasertchai T.; Piyaphanee W. | 2018 | No drivers/impacts identified; |
| Importance of antimicrobial stewardship to the English National Health Service. | Dixon, Jill; Duncan, Christopher Ja | 2014 | Narrative Review; |
| Implementation and impact of pediatric antimicrobial stewardship programs: a systematic scoping review. | Dona, D; Barbieri, E; Daverio, M; Lundin, R; Giaquinto, C; Zaoutis, T; Sharland, M | 2020 | No drivers/impacts identified; |
| Methicillin-resistant staphylococci: implications for our food supply?. | Doyle, M Ellin; Hartmann, Faye A; Lee Wong, Amy C | 2012 | Narrative Review; |
| Healthcare-Associated Infection Prevention Interventions for Neonates in Resource-Limited Settings. | Dramowski, Angela; Aucamp, Marina; Beales, Emily; Bekker, Adrie; Cotton, Mark Frederic; Fitzgerald, Felicity C; Labi, Appiah-Korang; Russell, Neal; Strysko, Jonathan; Whitelaw, Andrew; Coffin, Susan | 2022 | Narrative Review; |
| Bacterial pathogens and resistance causing community acquired paediatric bloodstream infections in low- And middle-income countries: A systematic review and meta-analysis | Droz N.; Hsia Y.; Ellis S.; Dramowski A.; Sharland M.; Basmaci R. | 2019 | No drivers/impacts identified; |
| Mycoplasma bovis Infections-Occurrence, Diagnosis and Control. | Dudek, Katarzyna; Nicholas, Robin A J; Szacawa, Ewelina; Bednarek, Dariusz | 2020 | Narrative Review; |
| Global advocacy needed for sepsis in children. | Dugani, Sagar; Kissoon, Niranjan | 2017 | Narrative Review; |
| Challenges and Strategies for Prevention of Multidrug-Resistant Organism Transmission in Nursing Homes | Dumyati G.; Stone N.D.; Nace D.A.; Crnich C.J.; Jump R.L.P. | 2017 | Narrative Review; |
| Encouraging the Development of New Antibiotics: Are Financial Incentives the Right Way Forward? A Systematic Review and Case Study. | Dutescu, Ilinca A; Hillier, Sean A | 2021 | No quantification done; |
| Strategies and challenges of antimicrobial stewardship in long-term care facilities. | Dyar, O J; Pagani, L; Pulcini, C | 2015 | Narrative Review; |
| How can we improve antibiotic prescribing in primary care?. | Dyar, Oliver J; Beovic, Bojana; Vlahovic-Palcevski, Vera; Verheij, Theo; Pulcini, Celine | 2016 | No quantification done; |
| Antimicrobial stewardship programs: Effects on clinical and economic outcomes and future directions | Eckart J.; Hogan M.; Mao Y.; Toscani M.; Brunetti L. | 2017 | Full text not available; |
| Non-prescribed antibiotic use for children at community levels in low- and middle-income countries: a systematic review and meta-analysis | Edessa D.; Assefa N.; Dessie Y.; Asefa F.; Dinsa G.; Oljira L. | 2022 | No AMR Analysis; |
| 2015 Evidence analysis library systematic review on advanced technology in food production. | Edge, M. S.; Kunkel, M. E.; Schmidt, J.; Papoutsakis, C. | 2018 | No AMR Analysis; |
| Impact of the Hajj on pneumococcal carriage and the effect of various pneumococcal vaccines. | Edouard, S.; Al-Tawfiq, J. A.; Memish, Z. A.; Yezli, S.; Gautret, P. | 2018 | No drivers/impacts identified; |
| Learning from our mistakes: using key opportunities to remove the perverse incentives that help drive antibiotic resistance. | Edwards, S E; Morel, C M | 2019 | Full text not available; |
| Knowledge, Attitudes and Perceptions of Medical Students on Antimicrobial Stewardship. | Efthymiou, Panagiotis; Gkentzi, Despoina; Dimitriou, Gabriel | 2020 | No drivers/impacts identified; |
| The antimicrobial susceptibility, resistance mechanisms and phylogenetic structure of S. Typhi isolated in 2005-2018 in the Russian Federation | Egorova S.A.; Kuleshov K.V.; Kaftyreva L.A.; Matveeva Z.N. | 2020 | Wrong study design; |
| A Systematic Review of Pneumococcal Carriage, Disease, Antimicrobial Resistance, and Vaccination in Egyptian Children Aged 18 Years and Younger. | El-Beleidy, Ahmed; El-Saied, Moustafa; Fasseeh, Nader; El Saie, Rehab Z; Haridy, Hammam | 2021 | No drivers/impacts identified; |
| The control of poultry salmonellosis using organic agents: an updated overview. | El-Saadony, Mohamed T; Salem, Heba M; El-Tahan, Amira M; Abd El-Mageed, Taia A; Soliman, Soliman M; Khafaga, Asmaa F; Swelum, Ayman A; Ahmed, Ahmed E; Alshammari, Fahdah A; Abd El-Hack, Mohamed E | 2022 | Narrative Review; |
| Antimicrobial stewardship in Tanzania: A consideration of strengths, weaknesses, opportunities and challenges for maintenance and further development of efforts | Eliakimu E. | 2016 | Narrative Review; |
| Antimicrobial stewardship in obstetric inpatients: A systematic review and narrative synthesis | Elkassem W.; Thomas B.; Abdulrouf P.; Alsoub H.; Alhail M.; Stewart D. | 2019 | Full text not available; |
| Multidrug-Resistant Acinetobacter baumannii Infections in the United Kingdom versus Egypt: Trends and Potential Natural Products Solutions. | Elwakil, Wafaa H; Rizk, Soha S; El-Halawany, Ali M; Rateb, Mostafa E; Attia, Ahmed S | 2023 | No drivers/impacts identified; |
| The Current State of Antimicrobial Stewardship: Challenges, Successes, and Future Directions | Emberger J.; Tassone D.; Stevens M.P.; Markley J.D. | 2018 | Narrative Review; |
| Antibiotics and toothache: A social media review | Emmott R.; Barber S.K.; Thompson W. | 2021 | No quantification done; |
| Vaccination externalities: The concept and application in pharmacoeconomic studies | Endarti D.; Riewpaiboon A. | 2016 | No drivers/impacts identified; |
| New Biomarkers and Diagnostic Tools for the Management of Fever in Low- and Middle-Income Countries: An Overview of the Challenges. | Escadafal, Camille; Nsanzabana, Christian; Archer, Julie; Chihota, Violet; Rodriguez, William; Dittrich, Sabine | 2017 | Narrative Review; |
| The epidemiology of carbapenemases in Latin America and the Caribbean. | Escandon-Vargas, Kevin; Reyes, Sergio; Gutierrez, Sergio; Villegas, Maria Virginia | 2017 | Narrative Review; |
| Methicillin-resistant staphylococcus aureus skin and soft tissue infections impacting patient care | Estes K. | 2011 | Narrative Review; |
| Extended-spectrum beta-lactamases-producing gram-negative bacteria in companion animals: action is clearly warranted!. | Ewers, Christa; Grobbel, Mirjam; Bethe, Astrid; Wieler, Lothar H; Guenther, Sebastian | 2011 | Full text not available; |
| Antibiotics and bacterial resistance in the 21st century. | Fair, Richard J; Tor, Yitzhak | 2014 | Narrative Review; |
| Antimicrobial use at the end of life: a scoping review | Fairweather J.; Cooper L.; Sneddon J.; Seaton R.A. | 2020 | No quantification done; |
| Trends in the Prevalence of Amphotericin B-Resistance (AmBR) among Clinical Isolates of Aspergillus Species. | Fakhim, Hamed; Badali, Hamid; Dannaoui, Eric; Nasirian, Maryam; Jahangiri, Fateme; Raei, Maedeh; Vaseghi, Narges; Ahmadikia, Kazem; Vaezi, Afsane | 2022 | No drivers/impacts identified; |
| Improving the Quality of Hospital Antibiotic Use: Impact on Multidrug-Resistant Bacterial Infections in Children. | Fanelli, Umberto; Chine, Vincenzo; Pappalardo, Marco; Gismondi, Pierpacifico; Esposito, Susanna | 2020 | No quantification done; |
| The one health landscape in Sub-Saharan African countries | Fasina F.O.; Fasanmi O.G.; Makonnen Y.J.; Bebay C.; Bett B.; Roesel K. | 2021 | Wrong study design; |
| Tackling the emerging threat of antifungal resistance to human health | Fisher M.C.; Alastruey-Izquierdo A.; Berman J.; Bicanic T.; Bignell E.M.; Bowyer P.; Bromley M.; Bruggemann R.; Garber G.; Cornely O.A.; Gurr S.J.; Harrison T.S.; Kuijper E.; Rhodes J.; Sheppard D.C.; Warris A.; White P.L.; Xu J.; Zwaan B.; Verweij P.E. | 2022 | Narrative Review; |
| Antibiotic prescribing in long-term care facilities: a meta-synthesis of qualitative research. | Fleming, Aoife; Bradley, Colin; Cullinan, Shane; Byrne, Stephen | 2015 | No quantification done; |
| A scoping review on the influential cognitive constructs informing public AMR behavior compliance and the attribution of personal responsibility. | Fletcher-Miles, Hayley; Gammon, John | 2020 | No quantification done; |
| A scoping review to assess the impact of public education campaigns to affect behavior change pertaining to antimicrobial resistance. | Fletcher-Miles, Hayley; Gammon, John; Williams, Sharon; Hunt, Julian | 2020 | No quantification done; |
| Understanding the contribution of environmental factors in the spread of antimicrobial resistance. | Fletcher, Stephanie | 2015 | Narrative Review; |
| Inappropriate Management of Asymptomatic Patients with Positive Urine Cultures: A Systematic Review and Meta-analysis | Flokas M.E.; Andreatos N.; Alevizakos M.; Kalbasi A.; Onur P.; Mylonakis E. | 2017 | No Socioeconomic Analysis; |
| Influence of Socioeconomic and Environmental Determinants of Health on Human Infection and Colonization with Antibiotic-Resistant and Antibiotic-Associated Pathogens: A Scoping Review. | Forrester, Joseph D; Cao, Siqi; Schaps, Diego; Liou, Raymond; Patil, Advait; Stave, Christopher; Sokolow, Susanne H; Leo, Giulio De | 2022 | Full text not available; |
| Antibiotic resistance in the food chain: A developing country-perspective | Founou L.L.; Founou R.C.; Essack S.Y. | 2016 | Narrative Review; |
| Antimicrobial resistance in the farm-to-plate continuum: more than a food safety issue. | Founou, Luria L; Founou, Raspail C; Essack, Sabiha Y | 2021 | Narrative Review; |
| Clinical and economic impact of antibiotic resistance in developing countries: A systematic review and meta-analysis. | Founou, Raspail Carrel; Founou, Luria Leslie; Essack, Sabiha Yusuf | 2017 | No drivers/impacts identified; |
| Microplastics in the human digestive environment: A focus on the potential and challenges facing in vitro gut model development | Fournier E.; Etienne-Mesmin L.; Grootaert C.; Jelsbak L.; Syberg K.; Blanquet-Diot S.; Mercier-Bonin M. | 2021 | Narrative Review; |
| The Contribution of Wastewater to the Transmission of Antimicrobial Resistance in the Environment: Implications of Mass Gathering Settings. | Fouz, Nour; Pangesti, Krisna N A; Yasir, Muhammad; Al-Malki, Abdulrahman L; Azhar, Esam I; Hill-Cawthorne, Grant A; Abd El Ghany, Moataz | 2020 | No drivers/impacts identified; |
| Strategies Used for Implementing and Promoting Adherence to Antibiotic Guidelines in Low- and Lower-Middle-Income Countries: A Systematic Review. | Foxlee, Nicola D; Townell, Nicola; Heney, Claire; McIver, Lachlan; Lau, Colleen L | 2021 | No drivers/impacts identified; |
| Antibiotic Resistance in Pacific Island Countries and Territories: A Systematic Scoping Review. | Foxlee, Nicola D; Townell, Nicola; McIver, Lachlan; Lau, Colleen L | 2019 | No drivers/impacts identified; |
| The negative impact of antibiotic resistance. | Friedman, N D; Temkin, E; Carmeli, Y | 2016 | Narrative Review; |
| Antimicrobial Stewardship: The Need to Cover All Bases. | Friedman, N Deborah | 2013 | Narrative Review; |
| Review of Escherichia coli O157:H7 Prevalence, Pathogenicity, Heavy Metal and Antimicrobial Resistance, African Perspective. | Gambushe, Sydney M; Zishiri, Oliver T; El Zowalaty, Mohamed E | 2022 | Narrative Review; |
| Economic burden of antibiotic resistance: how much do we really know?. | Gandra, S; Barter, D M; Laxminarayan, R | 2014 | Narrative Review; |
| Multidrug-resistant salmonella: A raising calamity | Gangathraprabhu B.; Geethanjali S.; Baskaran N.; Murugan M. | 2019 | Narrative Review; |
| A one health perspective on dairy production and dairy food safety | Garcia S.N.; Osburn B.I.; Cullor J.S. | 2019 | Narrative Review; |
| Livestock keepers' reasons for doing and not doing things which governments, vets and scientists would like them to do. | Garforth, C | 2015 | Narrative Review; |
| Healthcare-associated versus community-acquired infections: a new challenge for science and society. | Gastmeier, Petra | 2010 | Narrative Review; |
| Is sequential therapy for eradication of helicobacter pylori equally effective all over the world? A meta-analytic approach | Gatta L.; Vaira D.; Scarpignato C. | 2014 | Wrong study design; |
| Sexual Dimorphism and Gender in Infectious Diseases | Gay L.; Melenotte C.; Lakbar I.; Mezouar S.; Devaux C.; Raoult D.; Bendiane M.-K.; Leone M.; Mege J.-L. | 2021 | No AMR Analysis; |
| Public Health Interventions Delivered by Pharmacy Professionals in Low- and Middle-Income Countries in Africa: A Systematic Scoping Review. | Gebresillassie, Begashaw Melaku; Howells, Kelly; Ashiru-Oredope, Diane | 2023 | No drivers/impacts identified; |
| Molecular Epidemiology of Infectious Zoonotic and Livestock Diseases. | Gebreyes, Wondwossen A; Jackwood, Daral; de Oliveira, Celso Jose Bruno; Lee, Chang-Won; Hoet, Armando E; Thakur, Siddhartha | 2020 | Narrative Review; |
| Extended-Spectrum Beta-Lactamases Producing Enterobacteriaceae in the USA Dairy Cattle Farms and Implications for Public Health | Gelalcha B.D.; Kerro Dego O. | 2022 | Narrative Review; |
| Systematic review and survey of neisseria gonorrhoeae antimicrobial resistance data in the Asia Pacific, 2011 to 2016 | George C.R.R.; Enriquez R.P.; Gatus B.J.; Whiley D.M.; Lo Y.-R.; Ishikawa N.; Wi T.; Lahra M.M. | 2019 | No Socioeconomic Analysis; |
| Uropathogenic Escherichia coli in India-an Overview on Recent Research Advancements and Trends. | Ghosh, Arunita; Bandyopadhyay, Debojyoty; Koley, Snehashis; Mukherjee, Mandira | 2021 | No Socioeconomic Analysis; |
| Prevalence of antibiotic resistance in Helicobacter pylori: A recent literature review. | Ghotaslou, Reza; Leylabadlo, Hamed Ebrahimzadeh; Asl, Yalda Mohammadzadeh | 2015 | No drivers/impacts identified; |
| Review: Mitigating the risks posed by intensification in livestock production: the examples of antimicrobial resistance and zoonoses. | Gilbert, W; Thomas, L F; Coyne, L; Rushton, J | 2021 | Narrative Review; |
| Prevalence of methicillin-resistant Staphylococcus aureus carriage among healthcare workers in South Asia in non-outbreak settings: A systematic review and meta-analysis | Giri S.; Ghimire A.; Mishra A.; Acharya K.; Kuikel S.; Tiwari A.; Mishra S.K. | 2023 | No drivers/impacts identified; |
| Streptococcus agalactiae maternal colonization, antibiotic resistance and serotype profiles in Africa: A meta-analysis | Gizachew M.; Tiruneh M.; Moges F.; Tessema B. | 2019 | No drivers/impacts identified; |
| Antibiotic stewardship: A review of successful, evidence-based primary care strategies. | Glasziou, Paul; Dartnell, Jonathan; Biezen, Ruby; Morgan, Mark; Manski-Nankervis, Jo-Anne | 2022 | Narrative Review; |
| Rise in the prevalence of resistance to extended-spectrum cephalosporins in the USA, nursing homes and antibiotic prescribing in outpatient and inpatient settings | Goldstein E. | 2021 | Narrative Review; |
| Antibiotic-Resistant Acinetobacter baumannii Increasing Success Remains a Challenge as a Nosocomial Pathogen. | Gonzalez-Villoria, Ana Maria; Valverde-Garduno, Veronica | 2016 | Narrative Review; |
| Methicillin-resistant Staphylococcus aureus (MRSA): Colonisation and pre-operative screening | Goyal N.; Miller A.; Tripathi M.; Parvizi J. | 2013 | Narrative Review; |
| Scoping review of approaches for improving antimicrobial stewardship in livestock farmers and veterinarians. | Gozdzielewska, L; King, C; Flowers, P; Mellor, D; Dunlop, P; Price, L | 2020 | Not human health; |
| Complexities in understanding antimicrobial resistance across domesticated animal, human, and environmental systems. | Graham, David W; Bergeron, Gilles; Bourassa, Megan W; Dickson, James; Gomes, Filomena; Howe, Adina; Kahn, Laura H; Morley, Paul S; Scott, H Morgan; Simjee, Shabbir; Singer, Randall S; Smith, Tara C; Storrs, Carina; Wittum, Thomas E | 2019 | Narrative Review; |
| To succeed, one health must win animal agriculture's stronger collaboration | Gray G.C.; Mazet J.A.K. | 2020 | Narrative Review; |
| A scoping review of the role of wildlife in the transmission of bacterial pathogens and antimicrobial resistance to the food Chain. | Greig, J; Rajic, A; Young, I; Mascarenhas, M; Waddell, L; LeJeune, J | 2015 | Not human health; |
| Gut Microbiota Dysbiosis in Postweaning Piglets: Understanding the Keys to Health | Gresse R.; Chaucheyras-Durand F.; Fleury M.A.; Van de Wiele T.; Forano E.; Blanquet-Diot S. | 2017 | Narrative Review; |
| The impact of anti-infective drug shortages on hospitals in the United States: Trends and causes | Griffith M.M.; Gross A.E.; Sutton S.H.; Bolon M.K.; Esterly J.S.; Patel J.A.; Postelnick M.J.; Zembower T.R.; Scheetz M.H. | 2012 | Narrative Review; |
| Use of Antibiotics Without a Prescription in the U.S. Population: A Scoping Review. | Grigoryan, Larissa; Germanos, George; Zoorob, Roger; Juneja, Shivanki; Raphael, Jean L; Paasche-Orlow, Michael K; Trautner, Barbara W | 2019 | No quantification done; |
| Carbapenem resistant enterobacteriaceae in Africa | Gulumbe B.H.; Ajibola O. | 2020 | Narrative Review; |
| Epidemiology of multidrug-resistant organisms in Africa | Gulumbe B.H.; Faggo A.A. | 2019 | Narrative Review; |
| Prevalence and risk factors for antibiotic utilization in Chinese children | Guo S.; Sun Q.; Zhao X.; Shen L.; Zhen X. | 2021 | No quantification done; |
| The burden of methicillin resistant staphylococcus aureus in surgical site infections: A review | Gupta B.B.; Soman K.C.; Bhoir L.; Gadahire M.; Patel B.; Ahdal J. | 2021 | No quantification done; |
| Cropping Up Crisis at the Nexus Between COVID-19 and Antimicrobial Resistance (AMR) in Africa: A Scoping Review and Synthesis of Early Evidence. | Gutema, Girma; Homa, Gadissa | 2022 | No AMR Analysis; |
| Organic contaminants in African aquatic systems: Current knowledge, health risks, and future research directions | Gwenzi W.; Chaukura N. | 2018 | Not human health; |
| Insects, Rodents, and Pets as Reservoirs, Vectors, and Sentinels of Antimicrobial Resistance. | Gwenzi, Willis; Chaukura, Nhamo; Muisa-Zikali, Norah; Teta, Charles; Musvuugwa, Tendai; Rzymski, Piotr; Abia, Akebe Luther King | 2021 | Narrative Review; |
| Understanding the virulence of Streptococcus suis: A veterinary, medical, and economic challenge. | Haas, B; Grenier, D | 2018 | Narrative Review; |
| Prevalence of blaNDM-producing Acinetobacter baumannii strains isolated from clinical samples around the world; a systematic review | Hajikhani B.; Sameni F.; Ghazanfari K.; Abdolali B.; Yazdanparast A.; Asarehzadegan Dezfuli A.; Nasiri M.J.; Goudarzi M.; Dadashi M. | 2023 | Full text not available; |
| Antimicrobial use, prescribing, and resistance in selected ten selected developing countries: A brief overview | Haque M. | 2017 | Narrative Review; |
| Prospects and challenges of precision medicine in lower-and middle-income countries: A brief overview | Haque M.; Islam T.; Sartelli M.; Abdullah A.; Dhingra S. | 2020 | No drivers/impacts identified; |
| PIN17 A Systematic Literature Review of the Disease and Economic Burden of Multidrug Resistance Gram Negative Infections (MRGN) in JAPAN | Hara T.; Ikeoka H.; Tawseef A.; Mitsuo R. | 2020 | Full text not available; |
| Association between health inequalities and antibiotic use in high-income countries: A scoping review | Harvey Eleanor J.; Finistrella Viviana C.E.; Diane A.-O. | 2022 | No quantification done; |
| Mapping hospital antimicrobial stewardship programmes in the Gulf Cooperation Council states against international standards: a systematic review | Hashad N.; Perumal D.; Stewart D.; Tonna A.P. | 2020 | No quantification done; |
| Innovative drugs, chemicals, and enzymes within the animal production chain. | Hassan, Yousef I; Lahaye, Ludovic; Gong, Max M; Peng, Jian; Gong, Joshua; Liu, Song; Gay, Cyril G; Yang, Chengbo | 2018 | Narrative Review; |
| Antimicrobial Stewardship in General Practice: A Scoping Review of the Component Parts. | Hawes, Lesley; Buising, Kirsty; Mazza, Danielle | 2020 | No quantification done; |
| Comparing public attitudes, knowledge, beliefs and behaviours towards antibiotics and antimicrobial resistance in Australia, United Kingdom, and Sweden (2010-2021): A systematic review, meta-analysis, and comparative policy analysis | Hawkins O.; Scott A.M.; Montgomery A.; Nicholas B.; Mullan J.; van Oijen A.; Degeling C. | 2022 | No AMR Analysis; |
| Global Distribution of Fluoroquinolone and Colistin Resistance and Associated Resistance Markers in Escherichia coli of Swine Origin - A Systematic Review and Meta-Analysis. | Hayer, Shivdeep Singh; Casanova-Higes, Alejandro; Paladino, Eliana; Elnekave, Ehud; Nault, Andre; Johnson, Timothy; Bender, Jeff; Perez, Andres; Alvarez, Julio | 2022 | Not human health; |
| Antibiotics and antibiotic resistant bacteria/genes in urban wastewater: A comparison of their fate in conventional treatment systems and constructed wetlands. | Hazra, Moushumi; Joshi, Himanshu; Williams, John B; Watts, Joy E M | 2022 | Narrative Review; |
| Funding healthcare-associated infection research: a systematic analysis of UK research investments, 1997-2010. | Head, M. G.; Fitchett, J. R.; Holmes, A. H.; Atun, R. | 2014 | No drivers/impacts identified; |
| Economic burden of antimicrobial resistance: An analysis of additional costs associated with resistant infections | Health Information and Quality Authority | 2021 | Narrative Review; |
| A Review of Antimicrobial Resistance in Poultry Farming within Low-Resource Settings. | Hedman, Hayden D; Vasco, Karla A; Zhang, Lixin | 2020 | Narrative Review; |
| A review of bacterial zoonoses and antimicrobial resistant (AMR) on grouper fish (epinepholus sp.) | Helmi A.M.; Mukti A.T.; Soegianto A.; Mahardika K.; Mastuti I.; Effendi M.H.; Plumeriastuti H. | 2020 | Narrative Review; |
| A Scoping Review of the Use of Social and Behavioral Change in Acute Care Antimicrobial Stewardship Initiatives | Hemenway A.N.; DuBois D.L. | 2022 | No drivers/impacts identified; |
| Unpacking factors influencing antimicrobial use in global aquaculture and their implication for management: a review from a systems perspective. | Henriksson, Patrik J G; Rico, Andreu; Troell, Max; Klinger, Dane H; Buschmann, Alejandro H; Saksida, Sonja; Chadag, Mohan V; Zhang, Wenbo | 2018 | Narrative Review; |
| Challenges and opportunities for antimicrobial stewardship in resource-rich and resource-limited countries. | Hijazi, Karolin; Joshi, Chaitanya; Gould, Ian M | 2019 | Narrative Review; |
| Modelling the Future Clinical and Economic Burden of Antimicrobial Resistance: The Feasibility and Value of Models to Inform Policy. | Hillock, Nadine T; Merlin, Tracy L; Turnidge, John; Karnon, Jonathan | 2022 | Narrative Review; |
| Systematic Surveillance and Meta-Analysis of Antimicrobial Resistance and Food Sources from China and the USA | Himanshu; R. Prudencio C.; da Costa A.C.; Leal E.; Chang C.-M.; Pandey R.P. | 2022 | No drivers/impacts identified; |
| Impact of multidrug-resistant Pseudomonas aeruginosa infection on patient outcomes. | Hirsch, Elizabeth B; Tam, Vincent H | 2010 | Narrative Review; |
| Antibiotic resistance in travellers' diarrhoeal disease, an external perspective. | Hitch, Geeta; Fleming, Naomi | 2018 | No drivers/impacts identified; |
| Epidemiology and antimicrobial resistance of Campylobacter spp. in animals in Sub-Saharan Africa: A systematic review. | Hlashwayo, Delfina Fernandes; Sigauque, Betuel; Bila, Custodio Gabriel | 2020 | Not human health; |
| Helicobacter pylori antibiotic resistance in the united states over the last 10 years: A systematic review and meta-analysis | Ho J.; Elfanagely Y.; Moss S. | 2021 | Full text not available; |
| Infectious Diseases and Mass Gatherings. | Hoang, Van-Thuan; Gautret, Philippe | 2018 | Narrative Review; |
| Emerging Antimicrobial Drug Resistance in Africa and Latin America: Search for Reasons. | Hoellein, Ludwig; Kaale, Eliangiringa; Mwalwisi, Yonah Hebron; Schulze, Marco H; Vetye-Maler, Carina; Holzgrabe, Ulrike | 2022 | Narrative Review; |
| The limited state of training on the social dimensions of antimicrobial resistance. | Hofstraat, Karlijn; Spaan, Vera F; de Vries, Daniel H | 2021 | No drivers/impacts identified; |
| Combating inappropriate use of medicines. | Holloway, Kathleen Anne | 2011 | Narrative Review; |
| Antimicrobial stewardship in inpatient settings in the Asia pacific region: A systematic review and meta-analysis | Honda H.; Ohmagari N.; Tokuda Y.; Mattar C.; Warren D.K. | 2017 | No drivers/impacts identified; |
| Tackling antimicrobial resistance in Bangladesh: A scoping review of policy and practice in human, animal and environment sectors. | Hoque, Roksana; Ahmed, Syed Masud; Naher, Nahitun; Islam, Mohammad Aminul; Rousham, Emily K; Islam, Bushra Zarin; Hassan, Shaikh | 2020 | No quantification done; |
| Antimicrobial uses for livestock production in developing countries | Hosain M.Z.; Lutful Kabir S.M.; Kamal M.M. | 2021 | Narrative Review; |
| First global antimicrobial stewardship survey: Interim analysis of non-UK European data | Howard P.; Pulcini C.; Nathwani D. | 2012 | Wrong study design; |
| Carbapenem-Resistant Acinetobacter baumannii and Enterobacteriaceae in South and Southeast Asia. | Hsu, Li-Yang; Apisarnthanarak, Anucha; Khan, Erum; Suwantarat, Nuntra; Ghafur, Abdul; Tambyah, Paul Anantharajah | 2017 | Narrative Review; |
| Consensus review of the epidemiology and appropriate antimicrobial therapy of complicated urinary tract infections in Asia-Pacific region. | Hsueh, Po-Ren; Hoban, Daryl J; Carmeli, Yehuda; Chen, Shey-Ying; Desikan, Sunita; Alejandria, Marissa; Ko, Wen-Chien; Binh, Tran Quang | 2011 | Narrative Review; |
| Interventions to reduce childhood antibiotic prescribing for upper respiratory infections: systematic review and meta-analysis. | Hu, Yanhong; Walley, John; Chou, Roger; Tucker, Joseph D; Harwell, Joseph I; Wu, Xinyin; Yin, Jia; Zou, Guanyang; Wei, Xiaolin | 2016 | No drivers/impacts identified; |
| Alarming regional differences in prevalence and antimicrobial susceptibility of group B streptococci in pregnant women: A systematic review and meta-analysis. | Huang, Jingya; Li, Shunming; Li, Ling; Wang, Xiaolin; Yao, Zhenjiang; Ye, Xiaohua | 2016 | No drivers/impacts identified; |
| Antibiotic use for respiratory tract infections among older adults living in long-term care facilities: a systematic review and meta-analysis. | Huang, Y; Wei, W I; Correia, D F; Ma, B H M; Tang, A; Yeoh, E K; Wong, S Y S; Ip, M; Kwok, K O | 2023 | No drivers/impacts identified; |
| The economic impact of antimicrobial stewardship programmes in hospitals: a systematic literature review | Huebner C.; Huebner N.-O.; Flessa S. | 2019 | No drivers/impacts identified; |
| Antibiotic prescribing in hospitals: a social and behavioural scientific approach. | Hulscher, Marlies E J L; Grol, Richard P T M; van der Meer, Jos W M | 2010 | Narrative Review; |
| A Systematic Review of Antimicrobial Stewardship Interventions to Improve Management of Bacteriuria in Hospitalized Adults | Humphrey M.; MacDonald G.; Neville H.; Helwig M.; Ramsey T.; MacKinnon H.; Sketris I.; Johnston L.; Black E.K. | 2022 | No drivers/impacts identified; |
| Prevalence of Shigella species and its drug resistance pattern in Ethiopia: A systematic review and meta-analysis | Hussen S.; Mulatu G.; Yohannes Kassa Z. | 2019 | No drivers/impacts identified; |
| Burden of bacterial resistance among neonatal infections in low income countries: how convincing is the epidemiological evidence?. | Huynh, Bich-Tram; Padget, Michael; Garin, Benoit; Herindrainy, Perlinot; Kermorvant-Duchemin, Elsa; Watier, Laurence; Guillemot, Didier; Delarocque-Astagneau, Elisabeth | 2015 | No drivers/impacts identified; |
| The emergence and evolution of antimicrobial resistance: Impact on a global scale. | Hwang, Andrew Y; Gums, John G | 2016 | Narrative Review; |
| Economic Evaluations on Antimicrobial Stewardship Programme: A Systematic Review. | Ibrahim, Nor Haizan; Maruan, Khalidah; Mohd Khairy, Hasryn Azzuar; Hong, Yet Hoi; Dali, Ahmad Fauzi; Neoh, Chin Fen | 2017 | No drivers/impacts identified; |
| A bottom-up view of antimicrobial resistance transmission in developing countries. | Ikhimiukor, Odion O; Odih, Erkison Ewomazino; Donado-Godoy, Pilar; Okeke, Iruka N | 2022 | Narrative Review; |
| Social-economic factors and irrational antibiotic use as reasons for antibiotic resistance of bacteria causing common childhood infections in primary healthcare. | Ilic, Katarina; Jakovljevic, Emil; Skodric-Trifunovic, Vesna | 2012 | No quantification done; |
| Risk factor, diagnosis, and current treatment of H. pylori Infection in Indonesia: A Literature Review. | Iman, Rizani Putri; Junita, Tiroy; Rachman, Rinaldo Indra; Syam, Ari Fahrial | 2021 | Narrative Review; |
| External societal costs of antimicrobial resistance in humans attributable to antimicrobial use in livestock | Innes G.K.; Randad P.R.; Korinek A.; Davis M.F.; Price L.B.; So A.D.; Heaney C.D. | 2019 | Narrative Review; |
| Antibiotics threats on vegetables and the perils of low income nations practices | Inyinbor A.A.; Tsopmo A.; Udenigwe C.C. | 2021 | Not human health; |
| Resistance mechanisms in Campylobacter jejuni. | Iovine, Nicole M | 2013 | Narrative Review; |
| Global health systems' data science approach for precision diagnosis of sepsis in early life. | Iregbu, Kenneth; Dramowski, Angela; Milton, Rebecca; Nsutebu, Emmanuel; Howie, Stephen R C; Chakraborty, Mallinath; Lavoie, Pascal M; Costelloe, Ceire E; Ghazal, Peter | 2022 | No drivers/impacts identified; |
| A systematic review of healthcare-associated infections in Africa: An antimicrobial resistance perspective. | Irek, Emmanuel O; Amupitan, Adewale A; Obadare, Temitope O; Aboderin, Aaron O | 2018 | No drivers/impacts identified; |
| Antimicrobial Resistance and Its Drivers-A Review. | Irfan, Mohammad; Almotiri, Alhomidi; AlZeyadi, Zeyad Abdullah | 2022 | Narrative Review; |
| Measuring antibiotic prescribing in hospitalised children in resource-poor countries: A systematic review | Irwin A.; Sharland M. | 2013 | No drivers/impacts identified; |
| Drivers of Antibiotic Resistance Transmissionin Low- and Middle-Income Countriesfrom a "One Health" Perspective-A Review. | Iskandar, Katia; Molinier, Laurent; Hallit, Souheil; Sartelli, Massimo; Catena, Fausto; Coccolini, Federico; Hardcastle, Timothy Craig; Roques, Christine; Salameh, Pascale | 2020 | No quantification done; |
| Surveillance of antimicrobial resistance in low- and middle-income countries: a scattered picture. | Iskandar, Katia; Molinier, Laurent; Hallit, Souheil; Sartelli, Massimo; Hardcastle, Timothy Craig; Haque, Mainul; Lugova, Halyna; Dhingra, Sameer; Sharma, Paras; Islam, Salequl; Mohammed, Irfan; Naina Mohamed, Isa; Hanna, Pierre Abi; Hajj, Said El; Jamaluddin, Nurul Adilla Hayat; Salameh, Pascale; Roques, Christine | 2021 | Narrative Review; |
| Highlighting the gaps in quantifying the economic burden of surgical site infections associated with antimicrobial-resistant bacteria. | Iskandar, Katia; Sartelli, Massimo; Tabbal, Marwan; Ansaloni, Luca; Baiocchi, Gian Luca; Catena, Fausto; Coccolini, Federico; Haque, Mainul; Labricciosa, Francesco Maria; Moghabghab, Ayad; Pagani, Leonardo; Hanna, Pierre Abi; Roques, Christine; Salameh, Pascale; Molinier, Laurent | 2019 | No quantification done; |
| Clinical and economic burden of pneumonia among adults in Latin America. | Isturiz, Raul E; Luna, Carlos M; Ramirez, Julio | 2010 | No drivers/impacts identified; |
| Macrolide Resistance in Bordetella pertussis: Current Situation and Future Challenges | Ivaska L.; Barkoff A.-M.; Mertsola J.; He Q. | 2022 | No drivers/impacts identified; |
| Assessing the impact of law enforcement to reduce over-the-counter (OTC) sales of antibiotics in low- and middle-income countries; a systematic literature review | Jacobs T.G.; Robertson J.; van den Ham H.A.; Iwamoto K.; Bak Pedersen H.; Mantel-Teeuwisse A.K. | 2019 | No Socioeconomic Analysis; |
| Diagnostic Bacteriology in District Hospitals in Sub-Saharan Africa: At the Forefront of the Containment of Antimicrobial Resistance. | Jacobs, Jan; Hardy, Liselotte; Semret, Makeda; Lunguya, Octavie; Phe, Thong; Affolabi, Dissou; Yansouni, Cedric; Vandenberg, Olivier | 2019 | Narrative Review; |
| Status of benzimidazole resistance in intestinal nematode populations of livestock in Brazil: A systematic review | Jaeger L.H.; Carvalho-Costa F.A. | 2017 | No Socioeconomic Analysis; |
| A systematic review of prescription pattern monitoring studies and their effectiveness in promoting rational use of medicines. | Jain, Shipra; Upadhyaya, Prerna; Goyal, Jaswant; Kumar, Abhijit; Jain, Pushpawati; Seth, Vikas; Moghe, Vijay V | 2015 | Narrative Review; |
| Easy Access to Antibiotics; Spread of Antimicrobial Resistance and Implementation of One Health Approach in India. | Jani, Kunal; Srivastava, Vibhaw; Sharma, Preeti; Vir, Aruna; Sharma, Avinash | 2021 | No quantification done; |
| Global Threat of Carbapenem-Resistant Gram-Negative Bacteria. | Jean, Shio-Shin; Harnod, Dorji; Hsueh, Po-Ren | 2022 | Narrative Review; |
| High burden of antimicrobial resistance in Asia. | Jean, Shio-Shin; Hsueh, Po-Ren | 2011 | Narrative Review; |
| Vulnerability and One Health assessment approaches for infectious threats from a social science perspective: a systematic scoping review. | Jeleff, Maren; Lehner, Lisa; Giles-Vernick, Tamara; Duckers, Michel L A; Napier, A David; Jirovsky-Platter, Elena; Kutalek, Ruth | 2022 | No AMR Analysis; |
| Clinical prediction tools for identifying antimicrobial-resistant organism (ARO) carriage on hospital admissions: a systematic review. | Jeon, D; Chavda, S; Rennert-May, E; Leal, J | 2023 | No drivers/impacts identified; |
| Etiology of travellers' diarrhea. | Jiang, Z D; DuPont, H L | 2017 | No AMR Analysis; |
| Self-Medication with Antibiotics during COVID-19 in the Eastern Mediterranean Region Countries: A Review. | Jirjees, Feras; Ahmed, Munazza; Sayyar, Somayeh; Amini, Monireh; Al-Obaidi, Hala; Aldeyab, Mamoon A | 2022 | No quantification done; |
| Quantifying the economic cost of antibiotic resistance and the impact of related interventions: Rapid methodological review, conceptual framework and recommendations for future studies | Jit M.; Ng D.H.L.; Luangasanatip N.; Sandmann F.; Atkins K.E.; Robotham J.V.; Pouwels K.B. | 2020 | No drivers/impacts identified; |
| Substandard drugs: a potential crisis for public health. | Johnston, Atholl; Holt, David W | 2014 | No quantification done; |
| Fecal carriage of antimicrobial-resistant enterobacteriaceae in healthy Korean adults | Joo E.-J.; Kim S.J.; Baek M.; Choi Y.; Seo J.; Yeom J.-S.; Ko K.S. | 2018 | Wrong study design; |
| A scoping review of maternal antibiotic prophylaxis in low- and middle-income countries: Comparison to WHO recommendations for prevention and treatment of maternal peripartum infection. | Jury, Imogen; Thompson, Kelly; Hirst, Jane E | 2021 | No drivers/impacts identified; |
| Assessing economic outcomes of pharmacist-led antibiotic stewardship programs: A systematic review | Kahaleh A.; Chan M.; Dzelil S. | 2017 | Full text not available; |
| A review on Sero diversity and antimicrobial resistance patterns of Shigella species in Africa, Asia and South America, 2001-2014. | Kahsay, Atsebaha Gebrekidan; Muthupandian, Saravanan | 2016 | No drivers/impacts identified; |
| Impact of antimicrobial stewardship in critical care: A systematic review | Kaki R.; Elligsen M.; Walker S.; Simor A.; Palmay L.; Daneman N. | 2011 | No drivers/impacts identified; |
| Antimicrobial Stewardship Programs in Resource Constrained Environments: Understanding and Addressing the Need of the Systems. | Kakkar, Ashish Kumar; Shafiq, Nusrat; Singh, Gurpreet; Ray, Pallab; Gautam, Vikas; Agarwal, Ritesh; Muralidharan, Jayashree; Arora, Pankaj | 2020 | Narrative Review; |
| Scoping Review of National Antimicrobial Stewardship Activities in Eight African Countries and Adaptable Recommendations. | Kamere, Nduta; Garwe, Sandra Tafadzwa; Akinwotu, Oluwatosin Olugbenga; Tuck, Chloe; Krockow, Eva M; Yadav, Sara; Olawale, Agbaje Ganiyu; Diyaolu, Ayobami Hassan; Munkombwe, Derick; Muringu, Eric; Muro, Eva Prosper; Kaminyoghe, Felix; Ayotunde, Hameedat Taiye; Omoniyei, Love; Lawal, Mashood Oluku; Barlatt, Shuwary Hughric Adekule; Makole, Tumaini J; Nambatya, Winnie; Esseku, Yvonne; Rutter, Victoria; Ashiru-Oredope, Diane | 2022 | No drivers/impacts identified; |
| Genesis of antibiotic resistance VIII: "Auto Medication", an universal practice that contravenes hardy-weinberg-castle principle (HWCP), escalates as a serious risk factor in mitigating AR pandemic | Kannan S.; Carrizales D.; Carrizales T.; Alvarez A.; Jouregui S. | 2016 | Full text not available; |
| Extended-spectrum beta-lactamase-producing strains among diarrhoeagenic Escherichia coli-prospective traveller study with literature review. | Kantele, Anu; Laaveri, Tinja | 2022 | Wrong study design; |
| Antimicrobial overuse and misuse in the community in Greece and link to antimicrobial resistance using methicillin-resistant S. aureus as an example. | Karakonstantis, Stamatis; Kalemaki, Dimitra | 2019 | No quantification done; |
| Systematic review and meta-analysis of clinical and economic outcomes from the implementation of hospital-based antimicrobial stewardship programs | Karanika S.; Paudel S.; Grigoras C.; Kalbasi A.; Mylonakis E. | 2016 | No drivers/impacts identified; |
| Prevalence of Primary Multidrug-resistant Helicobacter pylori in Children: A Systematic Review and Meta-analysis. | Karbalaei, Mohsen; Keikha, Masoud; Talebi Bezmin Abadi, Amin | 2022 | No drivers/impacts identified; |
| Meta-Analysis of Biofilm Formation, Antibiotic Resistance Pattern, and Biofilm-Related Genes in Pseudomonas aeruginosa Isolated from Clinical Samples | Karballaei Mirzahosseini H.; Hadadi-Fishani M.; Morshedi K.; Khaledi A. | 2020 | No Socioeconomic Analysis; |
| Antimicrobial resistance and management of invasive Salmonella disease | Kariuki S.; Gordon M.A.; Feasey N.; Parry C.M. | 2015 | Narrative Review; |
| Epidemiology, Nasopharyngeal Carriage, Serotype Prevalence, and Antibiotic Resistance of Streptococcus pneumoniae in Indonesia | Kartasasmita C.B.; Rezeki Hadinegoro S.; Kurniati N.; Triasih R.; Halim C.; Gamil A. | 2020 | No drivers/impacts identified; |
| Genetic diversity and risk factors for the transmission of antimicrobial resistance across human, animals and environmental compartments in East Africa: a review. | Katale, Bugwesa Z; Misinzo, Gerald; Mshana, Stephen E; Chiyangi, Harriet; Campino, Susana; Clark, Taane G; Good, Liam; Rweyemamu, Mark M; Matee, Mecky I | 2020 | No quantification done; |
| Human Streptococcus suis Infections in Thailand: Epidemiology, Clinical Features, Genotypes, and Susceptibility | Kerdsin A. | 2022 | No AMR Analysis; |
| Fluoroquinolones-resistant Shigella species in Iranian children: a meta-analysis | Khademi F.; Sahebkar A. | 2019 | No drivers/impacts identified; |
| Group B streptococcus drug resistance in pregnant women in Iran: a meta-analysis | Khademi F.; Sahebkar A. | 2020 | No drivers/impacts identified; |
| Turning the implausible to the plausible: Towards a better control of over the counter dispensing of antibiotics in Egypt | Khalil R.B. | 2012 | Full text not available; |
| Irrational use of antibiotics in children | Khalil S.; Toor K.M.; Khan H.S. | 2015 | Full text not available; |
| A systematic review of human pathogens carried by the housefly (Musca domestica L.). | Khamesipour, Faham; Lankarani, Kamran Bagheri; Honarvar, Behnam; Kwenti, Tebit Emmanuel | 2018 | Not human health; |
| Nosocomial infections and their control strategies | Khan H.A.; Ahmad A.; Mehboob R. | 2015 | Narrative Review; |
| Nosocomial infections: Epidemiology, prevention, control and surveillance | Khan H.A.; Baig F.K.; Mehboob R. | 2017 | Narrative Review; |
| Overview of antibiotic resistance | Khan R.; Kumar A. | 2017 | Full text not available; |
| ANTIMICROBIAL CONSUMPTION IN HOSPITALISED COVID-19 PATIENTS: A SYSTEMATIC REVIEW AND META-ANALYSIS | Khan S.; Hasan S.S.; Bond S.E.; Conway B.R.; Aldeyab M.A. | 2022 | No drivers/impacts identified; |
| Use of Phages to Treat Antimicrobial-Resistant Salmonella Infections in Poultry. | Khan, Md Abu Sayem; Rahman, Sabita Rezwana | 2022 | Narrative Review; |
| The growing incidence and severity of Clostridium difficile infection in inpatient and outpatient settings. | Khanna, Sahil; Pardi, Darrell S | 2010 | Narrative Review; |
| Nurses' and consumers' knowledge and understanding of antimicrobial stewardship and infection prevention and control in paediatrics | Kilpatrick M.; Bouchoucha S.; Hutchinson A. | 2019 | No quantification done; |
| Paediatric nurses', children's and parents' adherence to infection prevention and control and knowledge of antimicrobial stewardship: A systematic review. | Kilpatrick, Mataya; Hutchinson, Ana; Manias, Elizabeth; Bouchoucha, Stephane L | 2021 | No quantification done; |
| Global Trends in the Proportion of Macrolide-Resistant Mycoplasma pneumoniae Infections: A Systematic Review and Meta-analysis | Kim K.; Jung S.; Kim M.; Park S.; Yang H.-J.; Lee E. | 2022 | No drivers/impacts identified; |
| Advances in optimizing the prescription of antibiotics in outpatient settings. | King, Laura M; Fleming-Dutra, Katherine E; Hicks, Lauri A | 2018 | Narrative Review; |
| A process for developing a sustainable and scalable approach to community engagement: community dialogue approach for addressing the drivers of antibiotic resistance in Bangladesh. | King, R.; Hicks, J.; Rassi, C.; Shafique, M.; Deepa Barua; Prashanta Bhowmik; Das, M.; Elsey, H.; Questa, K.; Fariza Fieroze; Hamade, P.; Sameena Huque; Newell, J.; Rumana Huque | 2020 | No AMR Analysis; |
| Setting the standard: multidisciplinary hallmarks for structural, equitable and tracked antibiotic policy. | Kirchhelle, Claas; Atkinson, Paul; Broom, Alex; Chuengsatiansup, Komatra; Ferreira, Jorge Pinto; Fortane, Nicolas; Frost, Isabel; Gradmann, Christoph; Hinchliffe, Stephen; Hoffman, Steven J; Lezaun, Javier; Nayiga, Susan; Outterson, Kevin; Podolsky, Scott H; Raymond, Stephanie; Roberts, Adam P; Singer, Andrew C; So, Anthony D; Sringernyuang, Luechai; Tayler, Elizabeth; Rogers Van Katwyk, Susan; Chandler, Clare I R | 2020 | Narrative Review; |
| Staphylococcus aureus infections: transmission within households and the community. | Knox, Justin; Uhlemann, Anne-Catrin; Lowy, Franklin D | 2015 | Narrative Review; |
| The socioeconomic burden of antibiotic resistance in conflict-affected settings and refugee hosting countries: a systematic scoping review. | Kobeissi, E.; Menassa, M.; Moussally, K.; Repetto, E.; Soboh, I.; Hajjar, M.; Saleh, S.; Abu-Sittah, G. | 2021 | No drivers/impacts identified; |
| Prevalence of Primary Antimicrobial Resistance of H. pylori in Turkey: A Systematic Review | Kocazeybek B.; Tokman H.B. | 2016 | No drivers/impacts identified; |
| Redefining national security using the case of antimicrobial resistance: a literature review | Koenig E. | 2019 | Full text not available; |
| Invasive Pneumococcal Disease burden and PCV coverage in children under five in Southeast Asia: implications for India. | Kolhapure, Shafi; Yewale, Vijay; Agrawal, Ashish; Krishnappa, Pradyumna; Soumahoro, Lamine | 2021 | No drivers/impacts identified; |
| Environmental impacts of mass drug administration programs: exposures, risks, and mitigation of antimicrobial resistance. | Konopka, Joanna K; Chatterjee, Pranab; LaMontagne, Connor; Brown, Joe | 2022 | No drivers/impacts identified; |
| Mycoplasma genitalium and Trichomonas vaginalis: addressing disparities and promoting public health control of two emerging sexually transmitted infections. | Korich, Faye; Reddy, Neha G; Trent, Maria | 2020 | Narrative Review; |
| Surveys of knowledge and awareness of antibiotic use and antimicrobial resistance in general population: A systematic review | Kosiyaporn H.; Chanvatik S.; Issaramalai T.; Kaewkhankhaeng W.; Kulthanmanusorn A.; Saengruang N.; Witthayapipopsakul W.; Viriyathorn S.; Kirivan S.; Kunpeuk W.; Suphanchaimat R.; Lekagul A.; Tangcharoensathien V. | 2020 | No Socioeconomic Analysis; |
| Pharmaceutical effluent: a critical link in the interconnected ecosystem promoting antimicrobial resistance. | Kotwani, Anita; Joshi, Jyoti; Kaloni, Deeksha | 2021 | Narrative Review; |
| Technologies for detecting falsified and substandard drugs in low and middle-income countries | Kovacs S.; Hawes S.E.; Maley S.N.; Mosites E.; Wong L.; Stergachis A. | 2014 | No Socioeconomic Analysis; |
| Infections in homeless | Krcmery V. | 2012 | Wrong study design; |
| Balancing the risks to individual and society: a systematic review and synthesis of qualitative research on antibiotic prescribing behaviour in hospitals | Krockow E.M.; Colman A.M.; Chattoe-Brown E.; Jenkins D.R.; Perera N.; Mehtar S.; Tarrant C. | 2019 | No quantification done; |
| Prosociality in the social dilemma of antibiotic prescribing. | Krockow, Eva M; Tarrant, Carolyn; Colman, Andrew M | 2022 | No quantification done; |
| Mastitis treatment-Reduction in antibiotic usage in dairy cows. | Kromker, V; Leimbach, S | 2017 | Narrative Review; |
| Antibiotics versus no treatment for asymptomatic bacteriuria in residents of aged care facilities: a systematic review and meta-analysis | Krzyzaniak N.; Forbes C.; Clark J.; Scott A.M.; Del Mar C.; Bakhit M. | 2022 | No drivers/impacts identified; |
| Treatment of enteric fever (typhoid and paratyphoid fever) with cephalosporins | Kuehn R.; Stoesser N.; Eyre D.; Darton T.C.; Basnyat B.; Parry C.M. | 2022 | No AMR Analysis; |
| Environmental hotspots for antibiotic resistance genes. | Kunhikannan, Shalini; Thomas, Colleen J; Franks, Ashley E; Mahadevaiah, Sumana; Kumar, Sumana; Petrovski, Steve | 2021 | Narrative Review; |
| Measures Against Antimicrobial Resistance in Children in Japan: Current Status and Future Prospects | Kusama Y.; Ishiwada N. | 2022 | Full text not available; |
| Point prevalence survey of antimicrobial use and resistance from hospitals in sub-Saharan Africa | Kuti O.O.; Alori S. | 2019 | Full text not available; |
| Health Professions Digital Education on Antibiotic Management: Systematic Review and Meta-Analysis by the Digital Health Education Collaboration | Kyaw B.M.; Tudor Car L.; van Galen L.S.; van Agtmael M.A.; Costelloe C.E.; Ajuebor O.; Campbell J.; Car J. | 2019 | No drivers/impacts identified; |
| Carbapenem resistance in Pseudomonas aeruginosa and Acinetobacter baumannii in the nosocomial setting in Latin America. | Labarca, Jaime A; Salles, Mauro Jose Costa; Seas, Carlos; Guzman-Blanco, Manuel | 2016 | No drivers/impacts identified; |
| Mycobacterium avium complex pulmonary disease: new epidemiology and management concepts. | Lande, Leah; George, Jason; Plush, Theodore | 2018 | No AMR Analysis; |
| A review of antibiotic use in food animals: Perspective, policy, and potential | Landers T.F.; Cohen B.; Wittum T.E.; Larson E.L. | 2012 | No quantification done; |
| Human Health and Ocean Pollution. | Landrigan, Philip J; Stegeman, John J; Fleming, Lora E; Allemand, Denis; Anderson, Donald M; Backer, Lorraine C; Brucker-Davis, Francoise; Chevalier, Nicolas; Corra, Lilian; Czerucka, Dorota; Bottein, Marie-Yasmine Dechraoui; Demeneix, Barbara; Depledge, Michael; Deheyn, Dimitri D; Dorman, Charles J; Fenichel, Patrick; Fisher, Samantha; Gaill, Francoise; Galgani, Francois; Gaze, William H; Giuliano, Laura; Grandjean, Philippe; Hahn, Mark E; Hamdoun, Amro; Hess, Philipp; Judson, Bret; Laborde, Amalia; McGlade, Jacqueline; Mu, Jenna; Mustapha, Adetoun; Neira, Maria; Noble, Rachel T; Pedrotti, Maria Luiza; Reddy, Christopher; Rocklov, Joacim; Scharler, Ursula M; Shanmugam, Hariharan; Taghian, Gabriella; van de Water, Jeroen A J M; Vezzulli, Luigi; Weihe, Pal; Zeka, Ariana; Raps, Herve; Rampal, Patrick | 2020 | No Socioeconomic Analysis; |
| Does locally relevant, real-time infection epidemiological data improve clinician management and antimicrobial prescribing in primary care? A systematic review | Lane I.; Bryce A.; Ingle S.M.; Hay A.D. | 2018 | No drivers/impacts identified; |
| The second-hand effects of antibiotics: Communicating the public health risks of drug resistance | Langford B.J.; Daneman N.; Leung V.; Wu J.H.C.; Brown K.; Schwartz K.L.; Garber G. | 2019 | Narrative Review; |
| Mathematical modeling of the transmission and control of foodborne pathogens and antimicrobial resistance at preharvest. | Lanzas, Cristina; Lu, Zhao; Grohn, Yrjo T | 2011 | Narrative Review; |
| Access to effective antimicrobials: a worldwide challenge. | Laxminarayan, Ramanan; Matsoso, Precious; Pant, Suraj; Brower, Charles; Rottingen, John-Arne; Klugman, Keith; Davies, Sally | 2016 | Narrative Review; |
| Improving antibiotic prescribing for children in the resource-poor setting. | Le Doare, Kirsty; Barker, Charlotte I S; Irwin, Adam; Sharland, Mike | 2015 | Narrative Review; |
| Systematic Review of Antibiotic Resistance Rates Among Gram-Negative Bacteria in Children With Sepsis in Resource-Limited Countries. | Le Doare, Kirsty; Bielicki, Julia; Heath, Paul T; Sharland, Mike | 2015 | No Socioeconomic Analysis; |
| How externalities impact an evaluation of strategies to prevent antimicrobial resistance in health care organizations. | Leal, Jenine R; Conly, John; Henderson, Elizabeth Ann; Manns, Braden J | 2017 | No quantification done; |
| Emergence of drug resistant bacteria at the Hajj: A systematic review. | Leangapichart, Thongpan; Rolain, Jean-Marc; Memish, Ziad A; Al-Tawfiq, Jaffar A; Gautret, Philippe | 2017 | No quantification done; |
| Treatment of Infections in Young Infants in Low- and Middle-Income Countries: A Systematic Review and Meta-analysis of Frontline Health Worker Diagnosis and Antibiotic Access | Lee A.C.C.; Chandran A.; Herbert H.K.; Kozuki N.; Markell P.; Shah R.; Campbell H.; Rudan I.; Baqui A.H. | 2014 | No drivers/impacts identified; |
| Educational effectiveness, target, and content for prudent antibiotic use. | Lee, Chang-Ro; Lee, Jung Hun; Kang, Lin-Woo; Jeong, Byeong Chul; Lee, Sang Hee | 2015 | No quantification done; |
| Something borrowed, something new: A governance and social construction framework to investigate power relations and responses of diverse stakeholders to policies addressing antimicrobial resistance | Legido-Quigley H.; Khan M.S.; Durrance-Bagale A.; Hanefeld J. | 2019 | Narrative Review; |
| Antibiotic stewardship and horizontal infection control are more effective than screening, isolation and eradication. | Lemmen, S W; Lewalter, K | 2018 | Narrative Review; |
| Antimicrobial stewardship in hospitals: Does it work and can we do it?. | Levy Hara, Gabriel | 2014 | Narrative Review; |
| Addressing Antimicrobial Resistance: An Overview of Priority Actions to Prevent Suboptimal Antimicrobial Use in Food-Animal Production | Lhermie G.; Grohn Y.T.; Raboisson D. | 2017 | Narrative Review; |
| Identification and antimicrobial resistance of pathogens in neonatal septicemia in China-A meta-analysis | Li J.-Y.; Chen S.-Q.; Yan Y.-Y.; Hu Y.-Y.; Wei J.; Wu Q.-P.; Lin Z.-L.; Lin J. | 2018 | No drivers/impacts identified; |
| Bacterial resistance to antibacterial agents: Mechanisms, control strategies, and implications for global health | Li T.; Wang Z.; Guo J.; de la Fuente-Nunez C.; Wang J.; Han B.; Tao H.; Liu J.; Wang X. | 2022 | Narrative Review; |
| A critical review of antibiotic resistance in probiotic bacteria. | Li, Ting; Teng, Da; Mao, Ruoyu; Hao, Ya; Wang, Xiumin; Wang, Jianhua | 2020 | Narrative Review; |
| The Effect of Infection Control Interventions in Day-Care Facilities and Schools | Lidal, Ingeborg Beate; Austvoll-Dahlgren, Astrid; Berg, Rigmor C.; Mathisen, Mariann; Vist, Gunn E. | 2014 | No drivers/impacts identified; |
| Impact of national interventions to promote responsible antibiotic use: A systematic review | Lim J.M.; Singh S.R.; Duong M.C.; Legido-Quigley H.; Hsu L.Y.; Tam C.C. | 2020 | No drivers/impacts identified; |
| Surveillance strategies using routine microbiology for antimicrobial resistance in low- and middle-income countries. | Lim, Cherry; Ashley, Elizabeth A; Hamers, Raph L; Turner, Paul; Kesteman, Thomas; Akech, Samuel; Corso, Alejandra; Mayxay, Mayfong; Okeke, Iruka N; Limmathurotsakul, Direk; van Doorn, H Rogier | 2021 | No drivers/impacts identified; |
| Reducing inappropriate antibiotic prescribing in the residential care setting: current perspectives. | Lim, Ching Jou; Kong, David C M; Stuart, Rhonda L | 2014 | Narrative Review; |
| Antibiotic use in residential aged care facilities. | Lim, Ching Jou; Stuart, Rhonda L; Kong, David C M | 2015 | Narrative Review; |
| Manure as a potential hotspot for antibiotic resistance dissemination by horizontal gene transfer events | Lima T.; Domingues S.; Silva G.J.D. | 2020 | Narrative Review; |
| Optimizing antibiotic use in Indonesia: a systematic review and synthesis of current evidence to inform opportunities for intervention | Limato R.; Lazarus G.; Dernison P.; Mudia M.; Alamanda M.; Nelwan E.J.; Sinto R.; Karuniawati A.; van Doorn H.R.; Hamers R.L. | 2022 | No drivers/impacts identified; |
| Factors influencing inappropriate use of antibiotics in outpatient and community settings in China: a mixed-methods systematic review. | Lin LeeSa; Sun RuYu; Yao TingTing; Zhou XuDong; Harbarth, S. | 2020 | No quantification done; |
| Carriage Duration and Household Transmission of Enterobacterales Producing Extended-Spectrum Beta-Lactamase in the Community: A Systematic Review and Meta-Analysis | Ling W.; Peri A.M.; Furuya-Kanamori L.; Harris P.N.A.; Paterson D.L. | 2022 | Full text not available; |
| Healthcare-associated infections control and antimicrobial resistance restraint in china: A literature review | Liu X.L.; Yin X.; Sun J.; Mao Z.F.; Cui D.; Wang Q.; Liang X.H.; Li H. | 2016 | Full text not available; |
| Antimicrobial resistance in the Pacific Island countries and territories. | Loftus, Michael; Stewardson, Andrew; Naidu, Ravi; Coghlan, Ben; Jenney, Adam; Kepas, Jonila; Lavu, Evelyn; Munamua, Alex; Peel, Trisha; Sahai, Vinita; Tekoaua, Rosemary; Tudravu, Litia; Zinihite, Julie; Cheng, Allen; Rafai, Eric; Peleg, Anton | 2020 | Narrative Review; |
| Addressing antimicrobial resistance by improving access and quality of care-A review of the literature from East Africa. | Loosli, Kathrin; Davis, Alicia; Muwonge, Adrian; Lembo, Tiziana | 2021 | No quantification done; |
| Misprescription of antibiotics in primary care: A critical systematic review of its determinants | Lopez-Vazquez P.; Vazquez-Lago J.M.; Figueiras A. | 2012 | No quantification done; |
| A Systematic Review of Antimicrobial Stewardship Interventions in the Emergency Department | Losier M.; Ramsey T.D.; Wilby K.J.; Black E.K. | 2017 | No drivers/impacts identified; |
| Sequential vs. prolonged 14-day triple therapy for Helicobacter pylori eradication: The meta-analysis may be influenced by 'geographical weighting' | Losurdo G.; Leandro G.; Principi M.; Giorgio F.; Montenegro L.; Sorrentino C.; Ierardi E.; Di Leo A. | 2015 | No drivers/impacts identified; |
| A systematic review of 40 years of distribution and antimicrobial resistance of Vibrio cholerae in Iran with future perspective | Louhrasby V.; Ghayaz F.; Mirhosseini S.A.; Golmohammadi R. | 2020 | Full text not available; |
| Antimicrobial resistance in Chilean marine-farmed salmon: Improving food safety through One Health. | Lozano-Munoz, Ivonne; Wacyk, Jurij; Kretschmer, Cristina; Vasquez-Martinez, Yesseny; Martin, Marcelo Cortez-San | 2021 | Narrative Review; |
| A decade of antimicrobial resistance research in social science fields: a scientometric review. | Lu, Jiahui; Sheldenkar, Anita; Lwin, May Oo | 2020 | No drivers/impacts identified; |
| Mycoplasma bovis: Mechanisms of Resistance and Trends in Antimicrobial Susceptibility. | Lysnyansky, Inna; Ayling, Roger D | 2016 | Narrative Review; |
| Drivers of Irrational Use of Antibiotics in Europe. | Machowska, Anna; Stalsby Lundborg, Cecilia | 2018 | Narrative Review; |
| Evaluation of the health and healthcare system burden due to antimicrobial-resistant Escherichia coli infections in humans: a systematic review and meta-analysis | MacKinnon M.C.; Sargeant J.M.; Pearl D.L.; Reid-Smith R.J.; Carson C.A.; Parmley E.J.; McEwen S.A. | 2020 | No drivers/impacts identified; |
| Telestewardship programs support clinical care and improve fiscal outcomes across the continuum through partnership between hospitals and health systems: A systematic review | Mailig M.; Cookson N.A.; Schulz L.T. | 2022 | No drivers/impacts identified; |
| Antimicrobial Stewardship: Fighting Antimicrobial Resistance and Protecting Global Public Health. | Majumder, Md Anwarul Azim; Rahman, Sayeeda; Cohall, Damian; Bharatha, Ambadasu; Singh, Keerti; Haque, Mainul; Gittens-St Hilaire, Marquita | 2020 | No drivers/impacts identified; |
| Production systems and important antimicrobial resistant-pathogenic bacteria in poultry: a review. | Mak, Philip H W; Rehman, Muhammad Attiq; Kiarie, Elijah G; Topp, Edward; Diarra, Moussa S | 2022 | Narrative Review; |
| Phages for Africa: The Potential Benefit and Challenges of Phage Therapy for the Livestock Sector in Sub-Saharan Africa. | Makumi, Angela; Mhone, Amos Lucky; Odaba, Josiah; Guantai, Linda; Svitek, Nicholas | 2021 | Narrative Review; |
| Setting up laboratory-based antimicrobial resistance surveillance in low- and middle-income countries: lessons learned from Georgia. | Malania, Lile; Wagenaar, Inge; Karatuna, Onur; Tambic Andrasevic, Arjana; Tsereteli, David; Baidauri, Marine; Imnadze, Paata; Nahrgang, Saskia; Ruesen, Carolien | 2021 | No drivers/impacts identified; |
| Monitoring and managing antibiotic resistance in refugee children. | Maltezou, Helena C; Elhadad, Dana; Glikman, Daniel | 2017 | No quantification done; |
| Antimicrobial resistance and the current refugee crisis. | Maltezou, Helena C; Theodoridou, Maria; Daikos, George L | 2017 | Narrative Review; |
| Penicillin-Resistant trend of Streptococcus pneumoniae in Asia: A systematic review | Mamishi S.; Moradkhani S.; Mahmoudi S.; Hosseinpour - Sadeghi R.; Pourakbari B. | 2014 | No drivers/impacts identified; |
| Framework for establishing regulatory guidelines to control antibiotic resistance in treated effluents | Manaia C.M. | 2023 | Narrative Review; |
| Extended-spectrum beta-lactamase (ESBL)-producing Enterobacteriaceae and urinary tract infections in pregnant/post-partum women: a systematic review and meta-analysis | Mansouri F.; Sheybani H.; Javedani Masrour M.; Afsharian M. | 2019 | No drivers/impacts identified; |
| One Health surveillance approaches for melioidosis and glanders: The Malaysian perspective | Mariappan V.; Vellasamy K.M.; Anpalagar R.R.; Lim Y.-M.; Zainal Abidin N.; Subramaniam S.; Nathan S. | 2022 | Narrative Review; |
| OneHealth Approaches Contribute Towards Antimicrobial Resistance: Malaysian Perspective. | Mariappan, Vanitha; Vellasamy, Kumutha Malar; Mohamad, Nor Alia; Subramaniam, Sreeramanan; Vadivelu, Jamuna | 2021 | Narrative Review; |
| Antimicrobial resistance | Marston H.D.; Dixon D.M.; Knisely J.M.; Palmore T.N.; Fauci A.S. | 2016 | No quantification done; |
| The antibiotic resistance crisis, with a focus on the United States. | Martens, Evan; Demain, Arnold L | 2017 | Narrative Review; |
| Educational Activities for Students and Citizens Supporting the One-Health Approach on Antimicrobial Resistance. | Marvasi, Massimiliano; Casillas, Lilliam; Vassallo, Alberto; Purchase, Diane | 2021 | Narrative Review; |
| A systematic review of inpatient antimicrobial stewardship programmes involving clinical pharmacists in small-to-medium-sized hospitals | Mas-Morey P.; Valle M. | 2018 | No drivers/impacts identified; |
| Communication strategies for improving public awareness on appropriate antibiotic use: Bridging a vital gap for action on antibiotic resistance. | Mathew, Philip; Sivaraman, Satya; Chandy, Sujith | 2019 | Narrative Review; |
| System Mapping of Antimicrobial Resistance to Combat a Rising Global Health Crisis. | Matthiessen, Lea Ellen; Hald, Tine; Vigre, Hakan | 2022 | No quantification done; |
| Antibiotics for persistent cough or wheeze following acute bronchiolitis in children | McCallum G.B.; Plumb E.J.; Morris P.S.; Chang A.B. | 2017 | No AMR Analysis; |
| Knowledge Gaps in the Understanding of Antimicrobial Resistance in Canada. | McCubbin, Kayley D; Anholt, R Michele; de Jong, Ellen; Ida, Jennifer A; Nobrega, Diego B; Kastelic, John P; Conly, John M; Gotte, Matthias; McAllister, Tim A; Orsel, Karin; Lewis, Ian; Jackson, Leland; Plastow, Graham; Wieden, Hans-Joachim; McCoy, Kathy; Leslie, Myles; Robinson, Joan L; Hardcastle, Lorian; Hollis, Aidan; Ashbolt, Nicholas J; Checkley, Sylvia; Tyrrell, Gregory J; Buret, Andre G; Rennert-May, Elissa; Goddard, Ellen; Otto, Simon J G; Barkema, Herman W | 2021 | Narrative Review; |
| A systematic review of the public's knowledge and beliefs about antibiotic resistance. | McCullough, A R; Parekh, S; Rathbone, J; Del Mar, C B; Hoffmann, T C | 2016 | No drivers/impacts identified; |
| Systematic evaluation of interventions to reduce overprescribing of antibiotics for acute respiratory tract infections | McDonagh M.S.; Peterson K.; Winthrop K.; Cantor A.; Holzhammer B.; Buckley D. | 2016 | Full text not available; |
| Measures used to assess the burden of ESBL-producing Escherichia coli infections in humans: a scoping review. | McDonald, Kathryn L; Garland, Sarah; Carson, Carolee A; Gibbens, Kimberly; Parmley, E Jane; Finley, Rita; MacKinnon, Melissa C | 2021 | No drivers/impacts identified; |
| Antimicrobial Resistance: a One Health Perspective. | McEwen, Scott A; Collignon, Peter J | 2018 | Narrative Review; |
| A systematic literature review and meta-analysis of factors associated with methicillin-resistant Staphylococcus aureus colonization at time of hospital or intensive care unit admission. | McKinnell, James A; Miller, Loren G; Eells, Samantha J; Cui, Eric; Huang, Susan S | 2013 | No Socioeconomic Analysis; |
| Is there a role for using mobile device applications to support antimicrobial stewardship? Preliminary findings from a survey of general practitioners in the United Kingdom | McLeod M.; Gharbi M.; Charani E.; Castro-Sanchez E.; Moore L.S.P.; Gilchrist M.; Holmes A. | 2014 | Wrong study design; |
| A systematic review of substandard, falsified, unlicensed and unregistered medicine sampling studies: a focus on context, prevalence, and quality. | McManus, D.; Naughton, B. D. | 2020 | No AMR Analysis; |
| What are the 'active ingredients' of interventions targeting the public's engagement with antimicrobial resistance and how might they work?. | McParland, Joanna L; Williams, Lynn; Gozdzielewska, Lucyna; Young, Mairi; Smith, Fraser; MacDonald, Jennifer; Langdridge, Darren; Davis, Mark; Price, Lesley; Flowers, Paul | 2018 | No drivers/impacts identified; |
| Antimicrobial Resistance in Rural Settings in Latin America: A Scoping Review with a One Health Lens. | Medina-Pizzali, Maria Luisa; Hartinger, Stella M; Salmon-Mulanovich, Gabriela; Larson, Anika; Riveros, Maribel; Mausezahl, Daniel | 2021 | No quantification done; |
| Surveillance systems to monitor antimicrobial resistance in Neisseria gonorrhoeae: a global, systematic review, 1 January 2012 to 27 September 2020. | Medland, N. A.; Zhang Ye; Praveena Gunaratnam; Lewis, D. A.; Donovan, B.; Whiley, D. M.; Guy, R. J.; Kaldor, J. M. | 2022 | No drivers/impacts identified; |
| State of Knowledge on the Acquisition, Diversity, Interspecies Attribution and Spread of Antimicrobial Resistance between Humans, Animals and the Environment: A Systematic Review | Meier H.; Spinner K.; Crump L.; Kuenzli E.; Schuepbach G.; Zinsstag J. | 2023 | No quantification done; |
| A Review of Evidence-Based Care of Symptomatic Trichomoniasis and Asymptomatic Trichomonas vaginalis Infections. | Meites, Elissa; Gaydos, Charlotte A; Hobbs, Marcia M; Kissinger, Patricia; Nyirjesy, Paul; Schwebke, Jane R; Secor, W Evan; Sobel, Jack D; Workowski, Kimberly A | 2015 | No drivers/impacts identified; |
| Human and avian extraintestinal pathogenic Escherichia coli: infections, zoonotic risks, and antibiotic resistance trends. | Mellata, Melha | 2013 | Narrative Review; |
| Serotype distribution and antimicrobial resistance patterns of invasive pneumococcal disease isolates from children in Mainland China - a systematic review. | Men WeiDong; Dong QiaoLi; Shi Wei; Yao KaiHu | 2020 | No drivers/impacts identified; |
| Maximising access to achieve appropriate human antimicrobial use in low-income and middle-income countries. | Mendelson, Marc; Rottingen, John-Arne; Gopinathan, Unni; Hamer, Davidson H; Wertheim, Heiman; Basnyat, Buddha; Butler, Christopher; Tomson, Goran; Balasegaram, Manica | 2016 | Narrative Review; |
| Surgical antibiotic prophylaxis in an era of antibiotic resistance: common resistant bacteria and wider considerations for practice | Menz B.D.; Charani E.; Gordon D.L.; Leather A.J.M.; Ramani Moonesinghe S.; Phillips C.J. | 2021 | Narrative Review; |
| Towards the just and sustainable use of antibiotics. | Merrett, Gemma L Buckland; Bloom, Gerald; Wilkinson, Annie; MacGregor, Hayley | 2016 | No quantification done; |
| Risk factors for carriage of antibiotic-resistant bacteria in healthy children in the community: A systematic review | Messina N.L.; Williamson D.A.; Robins-Browne R.; Bryant P.A.; Curtis N. | 2020 | Full text not available; |
| Lactococcus garvieae: an emerging bacterial pathogen of fish. | Meyburgh, C M; Bragg, R R; Boucher, C E | 2017 | Narrative Review; |
| The antimicrobial resistance crisis: causes, consequences, and management. | Michael, Carolyn Anne; Dominey-Howes, Dale; Labbate, Maurizio | 2014 | Narrative Review; |
| Prevention and control of nosocomial infections and resistance to antibiotics in Europe - Primum non-nocere: elements of successful prevention and control of healthcare-associated infections. | Mielke, Martin | 2010 | Narrative Review; |
| Aetiology and resistance in bacteraemias among adult and paediatric haematology and cancer patients | Mikulska M.; Viscoli C.; Orasch C.; Livermore D.M.; Averbuch D.; Cordonnier C.; Akova M. | 2014 | No drivers/impacts identified; |
| Impact of European pet antibiotic use on enterococci and staphylococci antimicrobial resistance and human health | Miranda C.; Silva V.; Igrejas G.; Poeta P. | 2021 | Full text not available; |
| Multi-Drug Resistant Coliform: Water Sanitary Standards and Health Hazards. | Mishra, Meerambika; Arukha, Ananta P; Patel, Amiya K; Behera, Niranjan; Mohanta, Tapan K; Yadav, Dhananjay | 2018 | Narrative Review; |
| Recent epidemiology of sexually transmissible enteric infections in men who have sex with men | Mitchell H.; Hughes G. | 2018 | No AMR Analysis; |
| Investigating forthcoming strategies to tackle deadly superbugs: current status and future vision. | Mitra, Saikat; Sultana, Sifat Ara; Prova, Shajuthi Rahman; Uddin, Tanvir Mahtab; Islam, Fahadul; Das, Rajib; Nainu, Firzan; Sartini, Sartini; Chidambaram, Kumarappan; Alhumaydhi, Fahad A; Emran, Talha Bin; Simal-Gandara, Jesus | 2022 | Narrative Review; |
| The growing challenges of antibacterial drug resistance in Ethiopia. | Moges, Feleke; Endris, Mengistu; Mulu, Andargachew; Tessema, Belay; Belyhun, Yeshambel; Shiferaw, Yitayal; Huruy, Kahsay; Unakal, Chandrashekhar; Kassu, Afework | 2014 | No drivers/impacts identified; |
| Epidemiology of common resistant bacterial pathogens in the countries of the Arab League | Moghnieh R.A.; Kanafani Z.A.; Tabaja H.Z.; Sharara S.L.; Awad L.S.; Kanj S.S. | 2018 | Narrative Review; |
| Prevalence of multi-drug resistant bacteria associated with foods and drinks in Nigeria (2015-2020): a systematic review. | Mola, I.; Onibokun, A.; Oranusi, S. | 2021 | Not human health; |
| Antimicrobial resistance of Staphylococcus aureus isolated from bovine mastitis: Systematic review and meta-analysis | Molineri A.I.; Camussone C.; Zbrun M.V.; Suarez Archilla G.; Cristiani M.; Neder V.; Calvinho L.; Signorini M. | 2021 | Not human health; |
| Is this pill an antibiotic or a painkiller? Improving the identification of oral antibiotics for better use | Monnier, Annelie A.; Do, Nga T. T.; Asante, Kwaku Poku; Afari-Asiedu, Samuel; Khan, Wasif Ali; Munguambe, Kh√°tia; Sevene, Esperanca; Tran, Toan K.; Nguyen, Chuc T. K.; Punpuing, Sureeporn; G√≥mez-Oliv√©, F. Xavier; Doorn, H. Rogier van; Caillet, C√©line; Newton, Paul N.; Ariana, Proochista; Wertheim, Heiman F. L.; Wertheim, Heiman FL; Sharmeen, Taniya; Boene, Helena; Matin, Mohammed Abdul; Phuong, Tuyet A.; Sunpuwan, Malee; Jarruruengpaisan, Wipaporn; Guill√©n, Georgina Pujol-Busquets; Ngobeni, Sizzy; Wafawanaka, Floidy | 2023 | Wrong study design; |
| Common infections in nursing homes: A review of current issues and challenges | Montoya A.; Mody L. | 2011 | Narrative Review; |
| Mechanisms of Antimicrobial Resistance (AMR) and Alternative Approaches to Overcome AMR. | Moo, Chew-Li; Yang, Shun-Kai; Yusoff, Khatijah; Ajat, Mokrish; Thomas, Warren; Abushelaibi, Aisha; Lim, Swee-Hua-Erin; Lai, Kok-Song | 2020 | Full text not available; |
| Impact of Antibiotics as Waste, Physical, Chemical, and Enzymatical Degradation: Use of Laccases. | Mora-Gamboa, Maria P C; Rincon-Gamboa, Sandra M; Ardila-Leal, Leidy D; Poutou-Pinales, Raul A; Pedroza-Rodriguez, Aura M; Quevedo-Hidalgo, Balkys E | 2022 | Narrative Review; |
| Review: Sustainable livestock systems: anticipating demand-side challenges. | Moran, D; Blair, K J | 2021 | Narrative Review; |
| European perspectives on efforts to reduce antimicrobial usage in food animal production | More S.J. | 2020 | Narrative Review; |
| Industry incentives and antibiotic resistance: an introduction to the antibiotic susceptibility bonus. | Morel, Chantal M; Lindahl, Olof; Harbarth, Stephan; de Kraker, Marlieke E A; Edwards, Suzanne; Hollis, Aidan | 2020 | No AMR Analysis; |
| Antimicrobial Resistance in Water in Latin America and the Caribbean: Available Research and Gaps | Moreno-Switt A.I.; Rivera D.; Caipo M.L.; Nowell D.C.; Adell A.D. | 2020 | No quantification done; |
| Non-prescription antimicrobial use worldwide: a systematic review. | Morgan, Daniel J; Okeke, Iruka N; Laxminarayan, Ramanan; Perencevich, Eli N; Weisenberg, Scott | 2011 | No quantification done; |
| Systematic review of patient-oriented interventions to reduce unnecessary use of antibiotics for upper respiratory tract infections | Mortazhejri S.; Hong P.J.; Yu A.M.; Hong B.Y.; Stacey D.; Bhatia R.S.; Grimshaw J.M. | 2020 | No quantification done; |
| Prevention of antimicrobial resistance in sub-Saharan Africa: What has worked? What still needs to be done?. | Moyo, Perseverance; Moyo, Enos; Mangoya, Derek; Mhango, Malizgani; Mashe, Tapfumanei; Imran, Mohd; Dzinamarira, Tafadzwa | 2023 | Narrative Review; |
| Antifungal stewardship in daily practice and health economic implications. | Munoz, Patricia; Valerio, Maricela; Vena, Antonio; Bouza, Emilio | 2015 | Narrative Review; |
| Factors potentially linked with the occurrence of antimicrobial resistance in selected bacteria from cattle, chickens and pigs: A scoping review of publications for use in modelling of antimicrobial resistance (IAM.AMR Project) | Murphy C.P.; Carson C.; Smith B.A.; Chapman B.; Marrotte J.; McCann M.; Primeau C.; Sharma P.; Parmley E.J. | 2018 | Not human health; |
| Advances in antibiotic drug discovery: Reducing the barriers for antibiotic development | Murphy K.E.; Sloan G.F.; Lawhern G.V.; Volk G.E.; Shumate J.T.; Wolfe A.L. | 2020 | Narrative Review; |
| The burden of extended-spectrum beta-lactamase-producing Enterobacteriaceae in Nigeria: a systematic review and meta-analysis | Musa B.M.; Imam H.; Lendel A.; Abdulkadir I.; Gumi H.S.; Aliyu M.H.; Habib A.G. | 2020 | No drivers/impacts identified; |
| Colonised and isolated: a qualitative metasynthesis of patients' experiences of being infected with multiple drug resistant organisms and subsequent isolation. | Mutsonziwa, G. A.; Green, J. | 2011 | No quantification done; |
| A review of animal health and drug use practices in India, and their possible link to antimicrobial resistance | Mutua F.; Sharma G.; Grace D.; Bandyopadhyay S.; Shome B.; Lindahl J. | 2020 | No quantification done; |
| Instruments used to measure knowledge and attitudes of healthcare professionals towards antibiotic use for the treatment of urinary tract infections: a systematic review. | Mwape, A. K.; Schmidtke, K. A.; Brown, C. | 2022 | No drivers/impacts identified; |
| Nursing home-associated bloodstream infection: A scoping review. | Mylotte, Joseph M | 2023 | No drivers/impacts identified; |
| Urban informal settlements as hotspots of antimicrobial resistance and the need to curb environmental transmission. | Nadimpalli, Maya L; Marks, Sara J; Montealegre, Maria Camila; Gilman, Robert H; Pajuelo, Monica J; Saito, Mayuko; Tsukayama, Pablo; Njenga, Sammy M; Kiiru, John; Swarthout, Jenna; Islam, Mohammad Aminul; Julian, Timothy R; Pickering, Amy J | 2020 | Narrative Review; |
| Trends in antimicrobial resistance in Malaysia | Naeemmudeen N.M.; Ghazali N.A.N.M.; Bahari H.; Ibrahim R.; Ahmad Dzulfikar Samsudin M.; Jasni A.S. | 2021 | Narrative Review; |
| Interventions for primary vesicoureteric reflux: An updated systematic review of randomised controlled trials | Nagler E.; Williams G.; Hodson E.; Craig J. | 2010 | No drivers/impacts identified; |
| Antibiotic resistance in the patient with cancer: Escalating challenges and paths forward. | Nanayakkara, Amila K; Boucher, Helen W; Fowler, Vance G Jr; Jezek, Amanda; Outterson, Kevin; Greenberg, David E | 2021 | Narrative Review; |
| Epidemiology of Invasive Early-Onset and Late-Onset Group B Streptococcal Disease in the United States, 2006 to 2015: Multistate Laboratory and Population-Based Surveillance | Nanduri S.A.; Petit S.; Smelser C.; Apostol M.; Alden N.B.; Harrison L.H.; Lynfield R.; Vagnone P.S.; Burzlaff K.; Spina N.L.; Dufort E.M.; Schaffner W.; Thomas A.R.; Farley M.M.; Jain J.H.; Pondo T.; McGee L.; Beall B.W.; Schrag S.J. | 2019 | Wrong study design; |
| The impact of antimicrobial stewardship strategies on antibiotic appropriateness and prescribing behaviours in selected countries in the Middle East: A systematic review | Nasr Z.; Paravattil B.; Wilby K.J. | 2017 | No drivers/impacts identified; |
| Prevalence of beta-lactamase and antibiotic-resistant Pseudomonas aeruginosa in the Arab region. | Nasser, Mahfouz; Gayen, Samarpita; Kharat, Arun S | 2020 | Wrong study design; |
| Clinical and economic consequences of hospital-acquired resistant and multidrug-resistant Pseudomonas aeruginosa infections: A systematic review and meta-analysis | Nathwani D.; Raman G.; Sulham K.; Gavaghan M.; Menon V. | 2014 | No drivers/impacts identified; |
| Evidence syntheses on the economic value of hospital antimicrobial stewardship programs [ASPS]-a call to action | Nathwani D.; Varghese D.; Lesher B.; Stephens J.M.; Ansari W.; Charbonneau C. | 2017 | Full text not available; |
| Value of hospital antimicrobial stewardship programs [ASPs]: A systematic review | Nathwani D.; Varghese D.; Stephens J.; Ansari W.; Martin S.; Charbonneau C. | 2019 | No drivers/impacts identified; |
| Scottish Antimicrobial Prescribing Group (SAPG): development and impact of the Scottish National Antimicrobial Stewardship Programme. | Nathwani, Dilip; Sneddon, Jacqueline; Malcolm, William; Wiuff, Camilla; Patton, Andrea; Hurding, Simon; Eastaway, Anne; Seaton, R Andrew; Watson, Emma; Gillies, Elizabeth; Davey, Peter; Bennie, Marion | 2011 | Narrative Review; |
| Estimating the burden of antimicrobial resistance: a systematic literature review | Naylor N.R.; Atun R.; Zhu N.; Kulasabanathan K.; Silva S.; Chatterjee A.; Knight G.M.; Robotham J.V. | 2018 | No drivers/impacts identified; |
| Is antimicrobial stewardship cost-effective? A narrative review of the evidence. | Naylor, N R; Zhu, N; Hulscher, M; Holmes, A; Ahmad, R; Robotham, J V | 2017 | Narrative Review; |
| Falsified and Substandard Drugs: Stopping the Pandemic. | Nayyar, Gaurvika M L; Breman, Joel G; Mackey, Tim K; Clark, John P; Hajjou, Mustapha; Littrell, Megan; Herrington, James E | 2019 | Narrative Review; |
| Self-medication with Antibiotics in WHO Southeast Asian Region: A Systematic Review. | Nepal, Gaurav; Bhatta, Shekhar | 2018 | No Socioeconomic Analysis; |
| Influences on independent nurse prescribers' antimicrobial prescribing behaviour: a systematic review. | Ness, Valerie; Price, Lesley; Currie, Kay; Reilly, Jacqueline | 2016 | No quantification done; |
| Microbiological food safety in Malaysia from the academician's perspective | New C.Y.; Ubong A.; Premarathne J.M.K.J.K.; Thung T.Y.; Lee E.; Chang W.S.; Loo Y.Y.; Kwan S.Y.; Tan C.W.; Kuan C.H.; Son R. | 2017 | Narrative Review; |
| An epidemiological meta-analysis on the worldwide prevalence, resistance and outcomes of spontaneous bacterial peritonitis in cirrhosis | Ng C.H.; Tay P.W.L.; Xiao J.; Tan D.J.H.; Lye Y.N.; Lim W.H.; Teo V.X.Y.; Heng R.R.Y.; Yeow M.W.X.; Lum L.H.W.; Xuan Tan E.X.; Kew G.S.; Lee G.H.; Muthiah M.D. | 2021 | No drivers/impacts identified; |
| Antimicrobial Usage and Antimicrobial Resistance in Animal Production in Southeast Asia: A Review. | Nhung, Nguyen T; Cuong, Nguyen V; Thwaites, Guy; Carrique-Mas, Juan | 2016 | Not human health; |
| Antimicrobial Resistance in Bacterial Poultry Pathogens: A Review. | Nhung, Nguyen Thi; Chansiripornchai, Niwat; Carrique-Mas, Juan J | 2017 | Not human health; |
| Combating the rise of antibiotic resistance in children. | Nicolini, G; Sperotto, F; Esposito, S | 2014 | Narrative Review; |
| Infection prevention issues in long-term care. | Nicolle, Lindsay E | 2014 | Narrative Review; |
| Emerging and Neglected Infectious Diseases: Insights, Advances, and Challenges | Nii-Trebi N.I. | 2017 | Narrative Review; |
| A Middle East systematic review and meta-analysis of prevalence and antibiotic susceptibility pattern in MRSA Staphylococcus aureus isolated from patients with cystic fibrosis. | Nikmanesh, Yousef; Foolady Azarnaminy, Afsaneh; Avishan, Pourya; Taheri, Mohammadreza; Sabeghi, Paniz; Najibzadeh, Ehsan; Khaledi, Azad | 2022 | No drivers/impacts identified; |
| Systematic review of antimicrobial resistance of clinical acinetobacter baumannii isolates in iran: An update | Nikoo H.R.; Ardebili A.; Mardaneh J. | 2017 | No Socioeconomic Analysis; |
| Shigella flexneri: an emerging pathogen. | Nisa, Iqbal; Qasim, Muhammad; Yasin, Nusrat; Ullah, Rafi; Ali, Anwar | 2020 | Narrative Review; |
| Prevalence of multidrug-, extensive drug-, and pandrug-resistant commensal Escherichia coli isolated from healthy humans in community settings in low- and middle-income countries: a systematic review and meta-analysis | Nkansa-Gyamfi N.A.; Kazibwe J.; Traore D.A.K.; Nji E. | 2019 | No drivers/impacts identified; |
| Freshwater environments as reservoirs of antibiotic resistant bacteria and their role in the dissemination of antibiotic resistance genes. | Nnadozie, Chika F; Odume, Oghenekaro Nelson | 2019 | Narrative Review; |
| Anthropogenic perturbations in marine microbial communities | Nogales B.; Lanfranconi M.P.; Pina-Villalonga J.M.; Bosch R. | 2011 | Narrative Review; |
| Metagenomics and Other Omics Approaches to Bacterial Communities and Antimicrobial Resistance Assessment in Aquacultures. | Nogueira, Teresa; Botelho, Ana | 2021 | Narrative Review; |
| Antimicrobial Resistance in Zambia: A Systematic Review | Nowbuth A.; Asombang A.; Tazikeng N.; Makinde O.; Sheets L. | 2022 | Full text not available; |
| Beyond Antimicrobial Use: A Framework for Prioritizing Antimicrobial Resistance Interventions | Noyes N.R.; Slizovskiy I.B.; Singer R.S. | 2021 | Narrative Review; |
| Antimicrobial resistance in sexually transmitted Shigella in men who have sex with men: A systematic review | O'Flanagan H.; Siddiq M.; Llewellyn C.; Richardson D. | 2023 | No quantification done; |
| Written information for patients (or parents of child patients) to reduce the use of antibiotics for acute upper respiratory tract infections in primary care | O'Sullivan J.W.; Harvey R.T.; Glasziou P.P.; Mccullough A. | 2016 | No Socioeconomic Analysis; |
| An overview of conventional and alternative strategies for developing new antibacterial agents. | Oancea, Simona | 2010 | Narrative Review; |
| Electronic information systems for One Health surveillance of antimicrobial resistance: A systematic scoping review | Oberin M.; Badger S.; Faverjon C.; Cameron A.; Bannister-Tyrrell M. | 2022 | No quantification done; |
| Review of the speculative role of co-infections in Streptococcus suis-associated diseases in pigs. | Obradovic, Milan R; Segura, Mariela; Segales, Joaquim; Gottschalk, Marcelo | 2021 | Narrative Review; |
| Epidemiology and antimicrobial resistance of staphylococci other than Staphylococcus aureus from domestic animals and livestock in Africa: a systematic review | Ocloo R.; Nyasinga J.; Munshi Z.; Hamdy A.; Marciniak T.; Soundararajan M.; Newton-Foot M.; Ziebuhr W.; Shittu A.; Revathi G.; Abouelfetouh A.; Whitelaw A. | 2022 | Not human health; |
| Social aspects of antibiotic use in the south and east asian students and general population | Oh J.M.; Ming L.C.; Bakrin F.S.; Goh B.H.; Lee L.H.; Khan T.M. | 2018 | No quantification done; |
| Antimicrobial stewardship programs in community hospitals: The evidence base and case studies | Ohl C.A.; Dodds Ashley E.S. | 2011 | Narrative Review; |
| Antimicrobial stewardship for inpatient facilities. | Ohl, Christopher A; Luther, Vera P | 2011 | Narrative Review; |
| Health care provider education as a tool to enhance antibiotic stewardship practices. | Ohl, Christopher A; Luther, Vera P | 2014 | Narrative Review; |
| Targeted literature review of the burden of extraintestinal pathogenic Escherichia Coli among elderly patients in Asia Pacific regions. | Ohmagari, Norio; Choi, Won Suk; Tang, Hung-Jen; Atanasov, Petar; Jiang, Xiaobin; Hernandez Pastor, Luis; Nakayama, Yoshikazu; Chiang, Jason; Lim, Kyunghwa; Nievera, Maria Carmen | 2023 | No drivers/impacts identified; |
| Recent Trends in the Epidemiology, Diagnosis, and Treatment of Macrolide-Resistant Mycoplasma pneumoniae | Oishi T.; Ouchi K. | 2022 | Narrative Review; |
| Antimicrobial stewardship and infection prevention interventions targeting healthcare-associated Clostridioides difficile and carbapenem-resistant Klebsiella pneumoniae infections: a scoping review. | Okeah, Bernard Ojiambo; Morrison, Valerie; Huws, Jaci C | 2021 | No drivers/impacts identified; |
| Laboratory systems as an antibacterial resistance containment tool in Africa | Okeke I.N. | 2016 | Narrative Review; |
| Leapfrogging laboratories: the promise and pitfalls of high-tech solutions for antimicrobial resistance surveillance in low-income settings. | Okeke, Iruka N; Feasey, Nicholas; Parkhill, Julian; Turner, Paul; Limmathurotsakul, Direk; Georgiou, Pantelis; Holmes, Alison; Peacock, Sharon J | 2020 | Narrative Review; |
| Food safety impacts of antimicrobial use and their residues in aquaculture. | Okocha, Reuben Chukwuka; Olatoye, Isaac Olufemi; Adedeji, Olufemi Bolarinwa | 2018 | Narrative Review; |
| Nurses and Antimicrobial Stewardship: Past, Present, and Future. | Olans, Rita Drummond; Hausman, Nicholas Bowditch; Olans, Richard Neal | 2020 | Narrative Review; |
| The landscape of antimicrobial resistance in the neonatal and multi-host pathogen group B Streptococcus: review from a One Health perspective | Oliveira L.M.A.; Simoes L.C.; Costa N.S.; Zadoks R.N.; Pinto T.C.A. | 2022 | Narrative Review; |
| Antimicrobial Resistance in Food Animals and the Environment in Nigeria: A Review. | Oloso, Nurudeen Olalekan; Fagbo, Shamsudeen; Garbati, Musa; Olonitola, Steve O; Awosanya, Emmanuel Jolaoluwa; Aworh, Mabel Kamweli; Adamu, Helen; Odetokun, Ismail Ayoade; Fasina, Folorunso Oludayo | 2018 | Not human health; |
| Occurrence of antibiotics in wastewater from hospital and convectional wastewater treatment plants and their impact on the effluent receiving rivers: current knowledge between 2010 and 2019. | Omuferen, Loveth Oke; Maseko, B; Olowoyo, J O | 2022 | Not human health; |
| A review of 40 years of enteric antimicrobial resistance research in Eastern Africa: What can be done better? | Omulo S.; Thumbi S.M.; Njenga M.K.; Call D.R. | 2015 | No quantification done; |
| Emergence of Community-Acquired, Multidrug-Resistant Invasive Nontyphoidal Salmonella Disease in Rural Western Kenya, 2009-2013. | Oneko, Martina; Kariuki, Simon; Muturi-Kioi, Vincent; Otieno, Kephas; Otieno, Vincent O; Williamson, John M; Folster, Jason; Parsons, Michele B; Slutsker, Laurence; Mahon, Barbara E; Hamel, Mary J | 2015 | Wrong study design; |
| An overview on bacterial resistance to antibiotics, essential drivers to resistance and their extensive threats | Onyancha W.; Sharma G.; Moin S.; Ali M.I. | 2020 | Full text not available; |
| Syphilis as Re-Emerging Disease, Antibiotic Resistance, and Vulnerable Population: Global Systematic Review and Meta-Analysis | Orbe-Orihuela Y.C.; Sanchez-Aleman M.A.; Hernandez-Pliego A.; Medina-Garcia C.V.; Vergara-Ortega D.N. | 2022 | No drivers/impacts identified; |
| Vancomycin resistant enterococci healthcare associated infections. | Orsi, G B; Ciorba, V | 2013 | No Socioeconomic Analysis; |
| Veterinary Anti-Microbial Resistance Containment in Bangladesh: evaluating the National Action Plan and scoping the evidence on implementation | Orubu E.S.F.; Zaman M.H.; Rahman M.T.; Wirtz V.J. | 2019 | Wrong study design; |
| Mapping the antimicrobial supply chain in Bangladesh: a scoping-review-based ecological assessment approach. | Orubu, E. S. F.; Samad, M. A.; Rahman, M. T.; Zaman, M. H.; Wirtz, V. J. | 2021 | No quantification done; |
| Risk factors for, and molecular epidemiology and clinical outcomes of, carbapenem- and polymyxin-resistant Gram-negative bacterial infections in pregnant women, infants, and toddlers: a systematic review and meta-analyses | Osei Sekyere J.; Reta M.A.; Bernard Fourie P. | 2021 | No drivers/impacts identified; |
| Epidemiology of antimicrobial resistance in Lebanese extra-hospital settings: An overview. | Osman, Marwan; Al Mir, Hiba; Rafei, Rayane; Dabboussi, Fouad; Madec, Jean-Yves; Haenni, Marisa; Hamze, Monzer | 2019 | Narrative Review; |
| Vibrio Pathogens: A Public Health Concern in Rural Water Resources in Sub-Saharan Africa. | Osunla, Charles A; Okoh, Anthony I | 2017 | Narrative Review; |
| A Systematic Review of Pharmacist-Led Antimicrobial Stewardship Programs in Sub-Saharan Africa. | Otieno, Phanice Ajore; Campbell, Sue; Maley, Sonny; Obinju Arunga, Tom; Otieno Okumu, Mitchel | 2022 | No quantification done; |
| Chapter 2: The Health and Economic Burden of Antimicrobial Resistance | Ouakrim, Driss Ait; Cassini, Alessandro; Cecchini, Michele; Plachouras, Diamantis | 2019 | Narrative Review; |
| Emergence and spread of antibiotic resistance in West Africa : contributing factors and threat assessment. | Ouedraogo, A S; Jean Pierre, H; Banuls, A L; Ouedraogo, R; Godreuil, S | 2017 | Not in English (in French); |
| Modeling the Health and Economic Impact of Substandard and Falsified Medicines: A Review of Existing Models and Approaches | Ozawa S.; Higgins C.R.; Nwokike J.I.; Phanouvong S. | 2022 | No AMR Analysis; |
| Measuring antibiotic consumption in low-income countries: a systematic review and integrative approach. | Padget, Michael; Guillemot, Didier; Delarocque-Astagneau, Elisabeth | 2016 | No drivers/impacts identified; |
| Knowledge, perceptions, and experiences of nurses in antimicrobial optimization or stewardship in the intensive care unit: a systematic review | Padigos J.; Reid S.; Kirby E.; Broom J. | 2020 | No quantification done; |
| Social and professional influences on antimicrobial prescribing for doctors-in-training: a realist review. | Papoutsi, Chrysanthi; Mattick, Karen; Pearson, Mark; Brennan, Nicola; Briscoe, Simon; Wong, Geoff | 2017 | No quantification done; |
| Socio-economic, industrial and cultural parameters of pig-borne infections. | Pappas, G | 2013 | Narrative Review; |
| Is AMR in Dairy Products a Threat to Human Health? An Updated Review on the Origin, Prevention, Treatment, and Economic Impacts of Subclinical Mastitis. | Paramasivam, Ragul; Gopal, Dhinakar Raj; Dhandapani, Ranjithkumar; Subbarayalu, Ramalakshmi; Elangovan, Mahesh Prabu; Prabhu, Bhavadharani; Veerappan, Veeramani; Nandheeswaran, Arunkumar; Paramasivam, Siddarth; Muthupandian, Saravanan | 2023 | Narrative Review; |
| Health Outcomes from Multidrug-Resistant Salmonella Infections in High-Income Countries: A Systematic Review and Meta-Analysis | Parisi A.; Crump J.A.; Glass K.; Howden B.P.; Furuya-Kanamori L.; Vilkins S.; Gray D.J.; Kirk M.D. | 2018 | No drivers/impacts identified; |
| Recent Insights into Aeromonas salmonicida and Its Bacteriophages in Aquaculture: A Comprehensive Review. | Park, Seon Young; Han, Jee Eun; Kwon, Hyemin; Park, Se Chang; Kim, Ji Hyung | 2020 | Narrative Review; |
| Staphylococcus aureus in Agriculture: Lessons in Evolution from a Multispecies Pathogen. | Park, Soyoun; Ronholm, Jennifer | 2021 | Narrative Review; |
| Tipping the balance: A systematic review and meta-ethnography to unfold the complexity of surgical antimicrobial prescribing behavior in hospital settings | Parker H.; Frost J.; Day J.; Bethune R.; Kajamaa A.; Hand K.; Robinson S.; Mattick K. | 2022 | No quantification done; |
| Control of Antimicrobial Resistance Requires an Ethical Approach. | Parsonage, Ben; Hagglund, Philip K; Keogh, Lloyd; Wheelhouse, Nick; Brown, Richard E; Dancer, Stephanie J | 2017 | Narrative Review; |
| Public Health Interventions to Improve Antimicrobial Resistance Awareness and Behavioural Change Associated with Antimicrobial Use: A Systematic Review Exploring the Use of Social Media. | Parveen, Sana; Garzon-Orjuela, Nathaly; Amin, Doaa; McHugh, Patricia; Vellinga, Akke | 2022 | No Socioeconomic Analysis; |
| Vertical transfer of antibiotics and antibiotic resistant strains across the mother/baby axis. | Patangia, Dhrati V; Ryan, C Anthony; Dempsey, Eugene; Stanton, Catherine; Ross, R Paul | 2022 | Narrative Review; |
| Variation in approaches to antimicrobial use surveillance in high-income secondary care settings: a systematic review. | Patel, Selina; Jhass, Arnoupe; Slee, Ann; Hopkins, Susan; Shallcross, Laura | 2021 | No Socioeconomic Analysis; |
| Prevalence and multidrug resistance in Salmonella enterica Typhimurium: an overview in South East Asia. | Patra, Saumya Darshana; Mohakud, Nirmal Kumar; Panda, Rakesh Kumar; Sahu, Bikash Ranjan; Suar, Mrutyunjay | 2021 | No drivers/impacts identified; |
| Vaccines for a sustainable planet. | Pecetta, Simone; Nandi, Arindam; Weller, Charlie; Harris, Vanessa; Fletcher, Helen; Berlanda Scorza, Francesco; Pizza, Mariagrazia; Salisbury, David; Moxon, Richard; Black, Steve; Bloom, David E; Rappuoli, Rino | 2023 | Narrative Review; |
| A european-wide study on the role of streptococcus pneumoniae in community-acquired pneumonia among adults: A meta-analysis | Pechlivanoglou P.; Rozenbaum M.; Van Der Werf T.; Lo-Ten-Foe J.; Postma M.; Hak E. | 2012 | No AMR Analysis; |
| Hospital-acquired infections due to gram-negative bacteria | Peleg A.Y.; Hooper D.C. | 2010 | Narrative Review; |
| Antibiotic-Resistant Bacteria in Aquaculture and Climate Change: A Challenge for Health in the Mediterranean Area. | Pepi, Milva; Focardi, Silvano | 2021 | Narrative Review; |
| The broader impacts of otitis media and sequelae for informing economic evaluations of pneumococcal conjugate vaccines. | Perdrizet, Johnna; Farkouh, Raymond A; Horn, Emily K; Hayford, Kyla; Sings, Heather L; Wasserman, Matt D | 2022 | Narrative Review; |
| How can Multi-Professional Education Support Better Stewardship?. | Pereira, Nuno Rocha; Castro-Sanchez, Enrique; Nathwani, Dilip | 2017 | Narrative Review; |
| Emerging infections and future threats | Petrosillo N. | 2019 | Narrative Review; |
| The burden of community-acquired bacterial pneumonia in the era of antibiotic resistance. | Peyrani, Paula; Mandell, Lionel; Torres, Antoni; Tillotson, Glenn S | 2019 | Narrative Review; |
| Methodological quality of studies evaluating the burden of drug-resistant infections in humans due to the WHO Global Antimicrobial Resistance Surveillance System target bacteria. | Pezzani, Maria Diletta; Tornimbene, Barbara; Pessoa-Silva, Carmem; de Kraker, Marlieke; Rizzardo, Sebastiano; Salerno, Nicola Duccio; Harbarth, Stephan; Tacconelli, Evelina | 2021 | No drivers/impacts identified; |
| A scoping review of factors potentially linked with antimicrobial-resistant bacteria from turkeys (iAM.AMR Project). | Phillips, C.; Chapman, B.; Agunos, A.; Carson, C. A.; Parmley, E. J.; Reid-Smith, R. J.; Smith, B. A.; Murphy, C. P. | 2022 | No Socioeconomic Analysis; |
| A systematic review and meta-analysis of integrated studies on antimicrobial resistance in Vietnam, with a focus on Enterobacteriaceae, from a One Health perspective. | Phu, Doan Hoang; Wongtawan, Tuempong; Truong, Dinh Bao; Van Cuong, Nguyen; Carrique-Mas, Juan; Thomrongsuwannakij, Thotsapol | 2022 | No drivers/impacts identified; |
| Encouraging rational antibiotic use in childhood pneumonia: a focus on Vietnam and the Western Pacific Region. | Phuong, Nguyen T K; Hoang, Tran T; Van, Pham H; Tu, Lolyta; Graham, Stephen M; Marais, Ben J | 2017 | Narrative Review; |
| Invasive community-associated MRSA infections: epidemiology and antimicrobial management. | Pichereau, Solen; Rose, Warren E | 2010 | Narrative Review; |
| Global Antimicrobial Stewardship with a Focus on Low- and Middle-Income Countries. | Pierce, Jacob; Apisarnthanarak, Anucha; Schellack, Natalie; Cornistein, Wanda; Maani, Amal Al; Adnan, Syamhanin; Stevens, Michael P | 2020 | Narrative Review; |
| Country Income Is Only One of the Tiles: The Global Journey of Antimicrobial Resistance among Humans, Animals, and Environment. | Pieri, Angela; Aschbacher, Richard; Fasani, Giada; Mariella, Jole; Brusetti, Lorenzo; Pagani, Elisabetta; Sartelli, Massimo; Pagani, Leonardo | 2020 | Narrative Review; |
| Achieving Antimicrobial Stewardship on the Global Scale: Challenges and Opportunities. | Pinto Ferreira, Jorge; Battaglia, Daniela; Dorado Garcia, Alejandro; Tempelman, KimAnh; Bullon, Carmen; Motriuc, Nelea; Caudell, Mark; Cahill, Sarah; Song, Junxia; LeJeune, Jeffrey | 2022 | Narrative Review; |
| Update on the burden of Campylobacter in developing countries | Platts-Mills J.A.; Kosek M. | 2014 | Narrative Review; |
| Antimicrobial resistance and over the counter use of drugs in Nepal. | Pokharel, Sunil; Adhikari, Bipin | 2020 | Narrative Review; |
| Systematic review of time lag between antibiotic use and rise of resistant pathogens among hospitalized adults in Europe. | Poku, Edith; Cooper, Katy; Cantrell, Anna; Harnan, Sue; Sin, Muna Abu; Zanuzdana, Arina; Hoffmann, Alexandra | 2023 | No Socioeconomic Analysis; |
| Last decade mini-review of the scientific progresses in the monitoring of the occurrence and antimicrobial susceptibility profile of poultry origin Campylobacter spp within the European Union countries. | Popa, S. A.; Morar, A.; Ban-Cucerzan, A.; Imre, K. | 2022 | Narrative Review; |
| Engaging patients in antimicrobial resistance and stewardship | Popescu I.; Neudorf K.; Kossey S.N. | 2016 | Wrong study design; |
| A review of the effects and production of spore-forming probiotics for poultry | Popov I.V.; Algburi A.; Prazdnova E.V.; Mazanko M.S.; Elisashvili V.; Bren A.B.; Chistyakov V.A.; Tkacheva E.V.; Trukhachev V.I.; Donnik I.M.; Ivanov Y.A.; Rudoy D.; Ermakov A.M.; Weeks R.M.; Chikindas M.L. | 2021 | Narrative Review; |
| The economic burden of antibiotic resistance: A systematic review and meta-analysis | Poudel, Ak Narayan; Zhu, Shihua; Cooper, Nicola; Little, Paul; Tarrant, Carolyn; Hickman, Matthew; Yao, Guiqing | 2023 | No drivers/impacts identified; |
| Impact of human Campylobacter infections in Southeast Asia: The contribution of the poultry sector. | Premarathne, Jayasekara Mudiyanselage Krishanthi Jayarukshi Kumari; Satharasinghe, Dilan Amila; Huat, John Tang Yew; Basri, Dayang Fredalina; Rukayadi, Yaya; Nakaguchi, Yoshitsugu; Nishibuchi, Mitsuaki; Radu, Son | 2017 | Narrative Review; |
| Antimicrobial resistance: a global multifaceted phenomenon. | Prestinaci, Francesca; Pezzotti, Patrizio; Pantosti, Annalisa | 2015 | Narrative Review; |
| Effectiveness of interventions to improve the public's antimicrobial resistance awareness and behaviours associated with prudent use of antimicrobials: A systematic review | Price L.; Gozdzielewska L.; Young M.; Smith F.; MacDonald J.; McParland J.; Williams L.; Langdridge D.; Davis M.; Flowers P. | 2018 | No Socioeconomic Analysis; |
| Antimicrobial stewardship in paediatrics. | Principi, Nicola; Esposito, Susanna | 2016 | Narrative Review; |
| Antimicrobial resistance: Prevalence, economic burden, mechanisms of resistance and strategies to overcome. | Pulingam, Thiruchelvi; Parumasivam, Thaigarajan; Gazzali, Amirah Mohd; Sulaiman, Azlinah Mohd; Chee, Jiun Yee; Lakshmanan, Manoj; Chin, Chai Fung; Sudesh, Kumar | 2022 | Narrative Review; |
| Community-acquired infant sepsis in hunan of China: Efficacy of who's currently recommended antibiotics-systematic review | Qiu Y.; Zhenghui Xiao X.Z.H.; Zhu Z.Y.M. | 2014 | Full text not available; |
| Anti-Biotics And Anti-Viral Drugs In Covid Management: Systemic Review Based On Current Evidence | Raghavendra Rao N.G.; Sheela M.A.; Sharma K.S.; Pathak A.; Tiwari A.; Kushwaha A.; Srivastava G. | 2022 | Full text not available; |
| Strategies to combat antimicrobial resistance in Indian scenario | Rahal A.; Kumar A. | 2021 | Narrative Review; |
| Determinants of worldwide antibiotic resistance dynamics across drug-bacterium pairs: a multivariable spatial-temporal analysis using ATLAS | Rahbe, Eve; Watier, Laurence; Guillemot, Didier; Glaser, Philippe; Opatowski, Lulla | 2023 | Wrong study design; |
| Initial inappropriate antibiotic therapy in hospitalized patients with gram-negative infections: Systematic review and meta-analysis | Raman G.; Avendano E.; Berger S.; Menon V.; Bartlett J. | 2014 | Full text not available; |
| Impact of mass and systematic antibiotic administration on antibiotic resistance in low- and middle-income countries? A systematic review | Rambliere L.; Guillemot D.; Delarocque-Astagneau E.; Huynh B.-T. | 2021 | No drivers/impacts identified; |
| What is the impact of mass or systematic antibiotic administration on antibiotic resistance in low middle-income countries? A systematic review | Rambliere L.; Guillemot D.; Delarocque-Astagneau E.; Huynh B.-T. | 2020 | No Socioeconomic Analysis; |
| A Public Health Insight into Salmonella in Poultry in Africa: A Review of the Past Decade: 2010-2020. | Ramtahal, Melissa A; Amoako, Daniel G; Akebe, Abia L K; Somboro, Anou M; Bester, Linda A; Essack, Sabiha Y | 2022 | Not Human Health |
| Antimicrobial resistance in Madagascar: A review of the current situation and challenges | Rasamiravaka T. | 2020 | No quantification done; |
| Self-medication and antibiotic resistance: Crisis, current challenges, and prevention. | Rather, Irfan A; Kim, Byung-Chun; Bajpai, Vivek K; Park, Yong-Ha | 2017 | Narrative Review; |
| Systematic review of electronic surveillance of infectious diseases with emphasis on antimicrobial resistance surveillance in resource-limited settings. | Rattanaumpawan, P.; Boonyasiri, A.; Vong, S.; Thamlikitkul, V. | 2018 | No drivers/impacts identified; |
| A systematic review and critical assessment of incentive strategies for discovery and development of novel antibiotics | Renwick M.J.; Brogan D.M.; Mossialos E. | 2016 | No quantification done; |
| Targeting innovation in antibiotic drug discovery and development: The need for a One Health - One Europe - One World Framework | Renwick, Matthew J; Simpkin, Victoria; Mossialos, Elias | 2016 | No quantification done; |
| Aquaculture at the crossroads of global warming and antimicrobial resistance | Reverter M.; Sarter S.; Caruso D.; Avarre J.-C.; Combe M.; Pepey E.; Pouyaud L.; Vega-Heredia S.; de Verdal H.; Gozlan R.E. | 2020 | No drivers/impacts identified; |
| Antibiotic reimbursement in a model delinked from sales: a benchmark-based worldwide approach. | Rex, John H; Outterson, Kevin | 2016 | Narrative Review; |
| The impact of the introduction of ten- or thirteen-valent pneumococcal conjugate vaccines on antimicrobial-resistant pneumococcal disease and carriage: A systematic literature review | Reyburn R.; Maher J.; von Mollendorf C.; Gwee A.; Mulholland K.; Russell F. | 2023 | No Socioeconomic Analysis; |
| Prevalence of cotrimoxazole resistance uropathogenic bacteria in Iran: A systematic review and meta-analysis | Rezaee M.; Ghafourian S.; Sayehmiri F.; Pakzad R.; Safiri S.; Pakzad I. | 2018 | No drivers/impacts identified; |
| Antimicrobial resistance associated with the use of antimicrobial processing aids during poultry processing operations: cause for concern?. | Rhouma, Mohamed; Romero-Barrios, Pablo; Gaucher, Marie-Lou; Bhachoo, Sujinder | 2021 | Not human health; |
| Surveillance and Control of Antibiotic Resistance in the Mediterranean Region. | Ricciardi, Walter; Giubbini, Gabriele; Laurenti, Patrizia | 2016 | No drivers/impacts identified; |
| Intensified food production and correlated risks to human health in the Greater Mekong Subregion: A systematic review | Richter C.H.; Custer B.; Steele J.A.; Wilcox B.A.; Xu J. | 2015 | No drivers/impacts identified; |
| Surgical Infections in Low- And Middle-Income Countries: A Global Assessment of the Burden and Management Needs | Rickard J.; Beilman G.; Forrester J.; Sawyer R.; Stephen A.; Weiser T.G.; Valenzuela J. | 2020 | Narrative Review; |
| PIN43 THE ECONOMIC BURDEN OF ANTIMICROBIAL RESISTANCE: RESULTS FROM A TARGETED LITERATURE REVIEW | Ringwala S.; Smallwood C.; Gala S.; Osman N. | 2019 | Full text not available; |
| Antibiotic-Resistant Acinetobacter baumannii in Low-Income Countries (2000-2020): Twenty-One Years and Still below the Radar, Is It Not There or Can They Not Afford to Look for It?. | Rizk, Soha S; Elwakil, Wafaa H; Attia, Ahmed S | 2021 | No drivers/impacts identified; |
| Educational Interventions to Reduce Prescription and Dispensing of Antibiotics in Primary Care: A Systematic Review of Economic Impact. | Rocha, Vania; Estrela, Marta; Neto, Vanessa; Roque, Fatima; Figueiras, Adolfo; Herdeiro, Maria Teresa | 2022 | No drivers/impacts identified; |
| Challenges of Empirical Antibiotic Therapy for Community-Acquired Pneumonia in Children. | Rodrigues, Charlene M C | 2017 | No drivers/impacts identified; |
| Antimicrobial peptides used as growth promoters in livestock production. | Rodrigues, Gisele; Maximiano, Mariana Rocha; Franco, Octavio Luiz | 2021 | Narrative Review; |
| Carbapenemases in Acinetobacter baumannii. Review of their dissemination in Latin America. | Rodriguez, Carlos Hernan; Nastro, Marcela; Famiglietti, Angela | 2018 | Narrative Review; |
| Country-to-country transfer of patients and the risk of multi-resistant bacterial infection | Rogers B.A.; Aminzadeh Z.; Hayashi Y.; Paterson D.L. | 2011 | Narrative Review; |
| Study reporting quality among interventions to reduce antibiotic use is a barrier to evidence-informed policymaking on antimicrobial resistance: systematic review | Rogers Van Katwyk S.; Grimshaw J.M.; Nkangu M.; Mendelson M.; Taljaard M.; Hoffman S.J. | 2020 | No drivers/impacts identified; |
| Strengthening the science of addressing antimicrobial resistance: a framework for planning, conducting and disseminating antimicrobial resistance intervention research. | Rogers Van Katwyk, S; Hoffman, S J; Mendelson, M; Taljaard, M; Grimshaw, J M | 2020 | No drivers/impacts identified; |
| Diagnostic testing for sepsis: A systematic review of economic evaluations | Rojas-Garcia P.; van der Pol S.; van Asselt A.D.I.; Postma M.J.; Rodriguez-Ibeas R.; Juarez-Castello C.A.; Gonzalez M.; Antonanzas F. | 2022 | No drivers/impacts identified; |
| AMR in low-resource settings: Medecins Sans Frontieres bridges surveillance gaps by developing a turnkey solution, the Mini-Lab. | Ronat, Jean-Baptiste; Natale, Alessandra; Kesteman, Thomas; Andremont, Antoine; Elamin, Wael; Hardy, Liselotte; Kanapathipillai, Rupa; Michel, Justine; Langendorf, Celine; Vandenberg, Olivier; Naas, Thierry; Kouassi, Felix | 2021 | No drivers/impacts identified; |
| A qualitative literature review exploring the drivers influencing antibiotic over-prescribing by GPs in primary care and recommendations to reduce unnecessary prescribing | Rose J.; Crosbie M.; Stewart A. | 2019 | No quantification done; |
| Vaccines Against Antimicrobial Resistance. | Rosini, Roberto; Nicchi, Sonia; Pizza, Mariagrazia; Rappuoli, Rino | 2020 | Narrative Review; |
| Can we 'WaSH' infectious diseases out of slums?. | Ross, Allen G; Rahman, Mahbubur; Alam, Munirul; Zaman, K; Qadri, Firdausi | 2020 | Narrative Review; |
| Globally Vibrio cholera antibiotics resistance to RNA and DNA effective antibiotics: A systematic review and meta-analysis. | Rostami, Amirabbas; Zadeh, Firoozeh Abolhasani; Ebrahimzadeh, Farnoosh; Jafari-Sales, Abolfazl; Gholami, Shakiba | 2022 | No drivers/impacts identified; |
| Healthcare-associated infections in sub-Saharan Africa. | Rothe, C; Schlaich, C; Thompson, S | 2013 | No quantification done; |
| Human, animal and environmental contributors to antibiotic resistance in low-resource settings: integrating behavioural, epidemiological and One Health approaches. | Rousham, Emily K; Unicomb, Leanne; Islam, Mohammad Aminul | 2018 | Narrative Review; |
| Guideline recommendations for antimicrobial stewardship education for clinical nursing practice in hospitals: A scoping review. | Rout, J; Essack, S; Brysiewicz, P | 2021 | No drivers/impacts identified; |
| Novel approaches to decrease inappropriate ambulatory antibiotic use. | Rowe, Theresa A; Linder, Jeffrey A | 2019 | Narrative Review; |
| Infection control issues in patients with haematological malignancies in the era of multidrug-resistant bacteria | Ruhnke M.; Arnold R.; Gastmeier P. | 2014 | Narrative Review; |
| Experiences of carriers of multidrug-resistant organisms: a systematic review | Rump B.; Timen A.; Verweij M.; Hulscher M. | 2019 | No quantification done; |
| The role of food chain in antimicrobial resistance spread and One Health approach to reduce risks. | Sagar, Prarthi; Aseem, Ajmal; Banjara, Santosh Kumar; Veleri, Shobi | 2023 | Full text not available; |
| Genetically modified bacteriophages. | Sagona, Antonia P; Grigonyte, Aurelija M; MacDonald, Paul R; Jaramillo, Alfonso | 2016 | No Socioeconomic Analysis; |
| Knowledge, perceptions and practices of community pharmacists towards antimicrobial stewardship: A systematic scoping review | Saha S.K.; Barton C.; Promite S.; Mazza D. | 2019 | No quantification done; |
| Infections and antimicrobial resistance in intensive care units in lower-middle income countries: a scoping review | Saharman Y.R.; Karuniawati A.; Severin J.A.; Verbrugh H.A. | 2021 | No Socioeconomic Analysis; |
| Limiting and controlling carbapenem-resistant Klebsiella pneumoniae | Saidel-Odes L.; Borer A. | 2014 | Narrative Review; |
| Non-prescription sales of antimicrobial agents at community pharmacies in developing countries: a systematic review | Sakeena M.H.F.; Bennett A.A.; McLachlan A.J. | 2018 | No quantification done; |
| Medical and pharmacy students' knowledge, attitude and perception concerning antimicrobial use and resistance in Pakistan | Saleem Z.; Azmi Hassali M.; Hashmi F.; Azhar F.; Mubarak R.; Afzaal A.; Munawar U. | 2019 | No quantification done; |
| Point Prevalence Surveys Of Antimicrobial Use: A Systematic Review And The Implications | Saleem Z.; Hassali M.A.; Godman B.; Versporten A.; Hashmi F.K.; Saeed H.; Saleem F.; Salman M.; Rehman I.U.; Khan T.M. | 2020 | No drivers/impacts identified; |
| Assessment of the perception of physicians concerning antibiotic use and resistance along with the factors influencing the prescription of antibiotics: A situational analysis from Pakistan | Saleem Z.; Hassali M.A.; Hashmi F.; Azhar F.; Hasan H.; Zaheer S.; Rehman I.U. | 2019 | Wrong study design; |
| Prevalence of Multidrug-Resistant and Extended-Spectrum Beta-Lactamase-Producing Shigella Species in Asia: A Systematic Review and Meta-Analysis. | Salleh, Mohd Zulkifli; Nik Zuraina, Nik Mohd Noor; Hajissa, Khalid; Ilias, Mohamad Ikram; Banga Singh, Kirnpal Kaur; Deris, Zakuan Zainy | 2022 | No drivers/impacts identified; |
| Prevalence of Multidrug-Resistant Diarrheagenic Escherichia coli in Asia: A Systematic Review and Meta-Analysis. | Salleh, Mohd Zulkifli; Nik Zuraina, Nik Mohd Noor; Hajissa, Khalid; Ilias, Mohamad Ikram; Deris, Zakuan Zainy | 2022 | No drivers/impacts identified; |
| Antimicrobial Resistance in the Food Chain: Trends, Mechanisms, Pathways, and Possible Regulation Strategies. | Samtiya, Mrinal; Matthews, Karl R; Dhewa, Tejpal; Puniya, Anil Kumar | 2022 | Narrative Review; |
| Candida auris: An Overview of the Emerging Drug-Resistant Fungal Infection. | Sanyaolu, Adekunle; Okorie, Chuku; Marinkovic, Aleksandra; Abbasi, Abu Fahad; Prakash, Stephanie; Mangat, Jasmine; Hosein, Zaheeda; Haider, Nafees; Chan, Jennifer | 2022 | No Socioeconomic Analysis; |
| Antibiotic Use in Low and Middle-Income Countries and the Challenges of Antimicrobial Resistance in Surgery. | Sartelli, Massimo; C Hardcastle, Timothy; Catena, Fausto; Chichom-Mefire, Alain; Coccolini, Federico; Dhingra, Sameer; Haque, Mainul; Hodonou, Adrien; Iskandar, Katia; Labricciosa, Francesco M; Marmorale, Cristina; Sall, Ibrahima; Pagani, Leonardo | 2020 | Narrative Review; |
| Prevalence of antibiotics prescription amongst patients with and without COVID-19 in low- and middle-income countries: a systematic review and meta-analysis | Satria Y.A.A.; Utami M.S.; Prasudi A. | 2022 | No AMR Analysis; |
| Global review and analysis of erythromycin in the environment: Occurrence, bioaccumulation and antibiotic resistance hazards. | Schafhauser, Bruno Henrique; Kristofco, Lauren A; de Oliveira, Cintia Mara Ribas; Brooks, Bryan W | 2018 | Narrative Review; |
| What drives antibiotic use in the community? a systematic review of determinants in the human outpatient sector. | Schmiege, D.; Evers, M.; Kistemann, T.; Falkenberg, T. | 2020 | No quantification done; |
| Review of antimicrobial resistance surveillance programmes in livestock and meat in EU with focus on humans. | Schrijver, R; Stijntjes, M; Rodriguez-Bano, J; Tacconelli, E; Babu Rajendran, N; Voss, A | 2018 | No drivers/impacts identified; |
| Economic evaluation of procalcitonin-guided antibiotic therapy in acute respiratory infections: A US health system perspective | Schuetz P.; Balk R.; Briel M.; Kutz A.; Christ-Crain M.; Stolz D.; Bouadma L.; Wolff M.; Kristoffersen K.B.; Wei L.; Burkhardt O.; Welte T.; Schroeder S.; Nobre V.; Tamm M.; Bhatnagar N.; Bucher H.C.; Luyt C.-E.; Chastre J.; Tubach F.; Mueller B.; Lacey M.J.; Ohsfeldt R.L.; Scheibling C.M.; Schneider J.E. | 2015 | Wrong study design; |
| Current challenges in the management of sepsis in ICUs in resource-poor settings and suggestions for the future. | Schultz, Marcus J; Dunser, Martin W; Dondorp, Arjen M; Adhikari, Neill K J; Iyer, Shivakumar; Kwizera, Arthur; Lubell, Yoel; Papali, Alfred; Pisani, Luigi; Riviello, Beth D; Angus, Derek C; Azevedo, Luciano C; Baker, Tim; Diaz, Janet V; Festic, Emir; Haniffa, Rashan; Jawa, Randeep; Jacob, Shevin T; Kissoon, Niranjan; Lodha, Rakesh; Martin-Loeches, Ignacio; Lundeg, Ganbold; Misango, David; Mer, Mervyn; Mohanty, Sanjib; Murthy, Srinivas; Musa, Ndidiamaka; Nakibuuka, Jane; Serpa Neto, Ary; Nguyen Thi Hoang, Mai; Nguyen Thien, Binh; Pattnaik, Rajyabardhan; Phua, Jason; Preller, Jacobus; Povoa, Pedro; Ranjit, Suchitra; Talmor, Daniel; Thevanayagam, Jonarthan; Thwaites, C Louise | 2017 | Narrative Review; |
| Recent advances in addressing the market failure of new antimicrobials: Learnings from NICE's subscription-style payment model. | Schurer, Marieke; Patel, Renu; van Keep, Marjolijn; Horgan, Jake; Matthijsse, Suzette; Madin-Warburton, Matthew | 2023 | No quantification done; |
| Current evidence on hospital antimicrobial stewardship objectives: A systematic review and meta-analysis | Schuts E.C.; Hulscher M.E.J.L.; Mouton J.W.; Verduin C.M.; Stuart J.W.T.C.; Overdiek H.W.P.M.; van der Linden P.D.; Natsch S.; Hertogh C.M.P.M.; Wolfs T.F.W.; Schouten J.A.; Kullberg B.J.; Prins J.M. | 2016 | No Socioeconomic Analysis; |
| Travel and the Spread of Drug-Resistant Bacteria. | Schwartz, Kevin L; Morris, Shaun K | 2018 | Narrative Review; |
| The quality of studies evaluating antimicrobial stewardship interventions: a systematic review | Schweitzer V.A.; van Heijl I.; van Werkhoven C.H.; Islam J.; Hendriks-Spoor K.D.; Bielicki J.; Bonten M.J.M.; Walker A.S.; Llewelyn M.J.; Harbarth S.; Huttner B.; Little P.; Rodriguez-Bano J.; Savoldi A.; van Smeden M.; Tacconelli E.; Timsit J.F.; Wolkewitz M. | 2019 | No drivers/impacts identified; |
| Economic Incentives for Antibacterial Drug Development: Literature Review and Considerations From the Transatlantic Task Force on Antimicrobial Resistance. | Sciarretta, Kimberly; Rottingen, John-Arne; Opalska, Aleksandra; Van Hengel, Arjon J; Larsen, Joseph | 2016 | Narrative Review; |
| Is antimicrobial administration to food animals a direct threat to human health? A rapid systematic review | Scott A.M.; Beller E.; Glasziou P.; Clark J.; Ranakusuma R.W.; Byambasuren O.; Bakhit M.; Page S.W.; Trott D.; Mar C.D. | 2018 | Wrong study design; |
| Antimicrobial Resistance in the Tropics. | Semret, Makeda; Haraoui, Louis-Patrick | 2019 | Full text not available; |
| CL2 SYSTEMATIC REVIEW AND METANALYSIS ON THE IMPACT OF MULTI-DRUG RESISTANT BACTERIA ON ECONOMIC AND CLINICAL OUTCOMES OF HEALTHCARE ACQUIRED INFECTIONS | Serra-Burriel M. | 2019 | No drivers/impacts identified; |
| Determinants of antibiotic dispensing without prescription: A systematic review | Servia-Dopazo M.; Figueiras A. | 2018 | No quantification done; |
| Hospital-Based Antimicrobial Stewardship Programs Used in Low- and Middle-Income Countries: A Scoping Review. | Setiawan, Eko; Abdul-Aziz, Mohd-Hafiz; Roberts, Jason A; Cotta, Menino Osbert | 2022 | Full text not available; |
| Shigella sonnei: virulence and antibiotic resistance | Shad A.A.; Shad W.A. | 2021 | Narrative Review; |
| Systematic review of antimicrobial stewardship in long-term care facilities: An opportunity for intervention | Shaeer K.; Cho J.; Zmarlicka M.; Worley M.; Hong J.; Tesh L. | 2018 | No drivers/impacts identified; |
| Shortage of essential antimicrobials: a major challenge to global health security. | Shafiq, Nusrat; Pandey, Avaneesh Kumar; Malhotra, Samir; Holmes, Alison; Mendelson, Marc; Malpani, Rohit; Balasegaram, Manica; Charani, Esmita | 2021 | Narrative Review; |
| Knowledge and attitude of physicians about antimicrobial resistance and their prescribing practices in services hospital, Lahore, Pakistan | Shahid A.; Iftikhar F.; Arshad M.K.; Javed Z.; Sufyan M.; Ghuman R.S.; Tarar Z. | 2017 | Wrong study design; |
| A systematic review on antibiotics misuse in livestock and aquaculture and regulation implications in China | Shao Y.; Wang Y.; Yuan Y.; Xie Y. | 2021 | No quantification done; |
| Global prevalence and distribution of vancomycin resistant, vancomycin intermediate and heterogeneously vancomycin intermediate Staphylococcus aureus clinical isolates: a systematic review and meta-analysis | Shariati A.; Dadashi M.; Moghadam M.T.; van Belkum A.; Yaslianifard S.; Darban-Sarokhalil D. | 2020 | No Socioeconomic Analysis; |
| The global prevalence of Daptomycin, Tigecycline, Quinupristin/Dalfopristin, and Linezolid-resistant Staphylococcus aureus and coagulase-negative staphylococci strains: a systematic review and meta-analysis. | Shariati, Aref; Dadashi, Masoud; Chegini, Zahra; van Belkum, Alex; Mirzaii, Mehdi; Khoramrooz, Seyed Sajjad; Darban-Sarokhalil, Davood | 2020 | No quantification done; |
| Menace of antimicrobial resistance in LMICs: Current surveillance practices and control measures to tackle hostility. | Sharma, Ayush; Singh, Akanksha; Dar, Mukhtar Ahmad; Kaur, Rimple Jeet; Charan, Jaykaran; Iskandar, Katia; Haque, Mainul; Murti, Krishna; Ravichandiran, V; Dhingra, Sameer | 2022 | No quantification done; |
| Advances in therapeutic and managemental approaches of bovine mastitis: a comprehensive review. | Sharun, Khan; Dhama, Kuldeep; Tiwari, Ruchi; Gugjoo, Mudasir Bashir; Iqbal Yatoo, Mohd; Patel, Shailesh Kumar; Pathak, Mamta; Karthik, Kumaragurubaran; Khurana, Sandip Kumar; Singh, Rahul; Puvvala, Bhavani; Amarpal; Singh, Rajendra; Singh, Karam Pal; Chaicumpa, Wanpen | 2021 | Narrative Review; |
| Interventions to optimize the use of antibiotics in China: A scoping review of evidence from humans, animals, and the environment from a One Health perspective. | Shen, Liyan; Wei, Xiaolin; Yin, Jia; Haley, D Rob; Sun, Qiang; Lundborg, Cecilia Stalsby | 2022 | No quantification done; |
| Urinary tract infections: Raising problem in developing countries | Shiralizadeh S.; Taghizadeh S.; Asgharzadeh M.; Shokouhi B.; Gholizadeh P.; Rahbar M.; Kafil H.S. | 2018 | No AMR Analysis; |
| MRSA compendium of epidemiology, transmission, pathophysiology, treatment, and prevention within one health framework | Shoaib M.; Aqib A.I.; Muzammil I.; Majeed N.; Bhutta Z.A.; Kulyar M.F.-E.-A.; Fatima M.; Zaheer C.-N.F.; Muneer A.; Murtaza M.; Kashif M.; Shafqat F.; Pu W. | 2023 | Narrative Review; |
| Identifying research gaps in antimicrobial resistance (AMR): Literature review, potential research questions and study designs | Silveira D.S.; Leite B.F.; Alves M.D. | 2015 | Full text not available; |
| A review of antimicrobial stewardship training in medical education. | Silverberg, Sarah L; Zannella, Vanessa E; Countryman, Drew; Ayala, Ana Patricia; Lenton, Erica; Friesen, Farah; Law, Marcus | 2017 | No quantification done; |
| Factors affecting the cost effectiveness of antibiotics | Simoens S. | 2011 | No quantification done; |
| Guidance for Demonstrating the Societal Value of new Antibiotics | Simoens S.; Spriet I. | 2020 | No drivers/impacts identified; |
| Incentivising innovation in antibiotic drug discovery and development: progress, challenges and next steps. | Simpkin, Victoria L; Renwick, Matthew J; Kelly, Ruth; Mossialos, Elias | 2017 | No AMR Analysis; |
| Antimicrobial Prescribing in the Telehealth Setting: Framework for Stewardship During a Period of Rapid Acceleration Within Primary Care. | Sine, Kathryn; Appaneal, Haley; Dosa, David; LaPlante, Kerry L | 2022 | Narrative Review; |
| Impact Of Different Antimicrobial Stewardship Strategies For Reducing Antimicrobial Resistance And Antimicrobial Uses - A Systematic Literature Review | Singh T.; Hussain M.; Maity M.K.; Shandilya K.; Wazir K.; Malik A.; Ndang M.; Kaur P.; Kumar A.; Das P.; Agrawal B.K. | 2022 | No drivers/impacts identified; |
| How do aged-care staff feel about antimicrobial stewardship? A systematic review of staff attitudes in long-term residential aged-care. | Singh, Saniya; Degeling, Chris; Fernandez, Dominic; Montgomery, Amy; Caputi, Peter; Deane, Frank P | 2022 | No quantification done; |
| Does Irrigation with Treated and Untreated Wastewater Increase Antimicrobial Resistance in Soil and Water: A Systematic Review. | Slobodiuk, Stacy; Niven, Caitlin; Arthur, Greer; Thakur, Siddhartha; Ercumen, Ayse | 2021 | Not human health; |
| PIN49 EXAMINING THE CLINICAL AND ECONOMIC IMPACT OF ANTIMICROBIAL RESISTANCE AT ACUTE CARE FACILITIES: A PRACTICAL TOOL | Smallwood C.; Gala S.; Chandran A.; Nakamachi Y.; Morris A. | 2019 | Full text not available; |
| Infections with Helicobacter pylori and challenges encountered in Africa | Smith S.; Fowora M.; Pellicano R. | 2019 | Narrative Review; |
| Helicobacter pylori infection in Africa: 2018 literature update | Smith S.I.; Seriki A.; Ndip R.; Pellicano R. | 2018 | Full text not available; |
| Inpatient Antimicrobial Stewardship in Pediatrics: A Systematic Review. | Smith, Michael J; Gerber, Jeffrey S; Hersh, Adam L | 2015 | No drivers/impacts identified; |
| New business models for antibiotic innovation. | So, Anthony D; Shah, Tejen A | 2014 | Narrative Review; |
| Prevalence of Mycoplasma genitalium infection among HIV PrEP users: A systematic review and meta-analysis | Sokoll P.R.; Migliavaca C.B.; Siebert U.; Schmid D.; Arvandi M. | 2023 | No drivers/impacts identified; |
| Community-Acquired Pneumonia in the Asia-Pacific Region. | Song, Jae-Hoon; Huh, Kyungmin; Chung, Doo Ryeon | 2016 | Narrative Review; |
| Clinical and economic burden of community-acquired pneumonia amongst adults in the Asia-Pacific region. | Song, Jae-Hoon; Thamlikitkul, Visanu; Hsueh, Po-Ren | 2011 | No drivers/impacts identified; |
| Strategies and novel technologies to control Campylobacter in the poultry chain: A review. | Soro, Arturo B; Whyte, Paul; Bolton, Declan J; Tiwari, Brijesh K | 2020 | Narrative Review; |
| Measuring Appropriate Antimicrobial Use: Attempts at Opening the Black Box. | Spivak, Emily S; Cosgrove, Sara E; Srinivasan, Arjun | 2016 | Narrative Review; |
| Antibiotic use for childhood diarrhea in low-and-middle-income countries: Re-analyses of survey data and systematic review of literature | Sreeramareddy C.; Mittal P. | 2020 | Full text not available; |
| In-Hospital Macro-, Meso-, and Micro-Drivers and Interventions for Antibiotic Use and Resistance: A Rapid Evidence Synthesis of Data from Canada and Other OECD Countries. | Stalteri Mastrangelo, Rosa; Hajizadeh, Anisa; Piggott, Thomas; Loeb, Mark; Wilson, Michael; Lozano, Luis Enrique Colunga; Roldan, Yetiani; El-Khechen, Hussein; Miroshnychenko, Anna; Thomas, Priya; Schunemann, Holger J; Nieuwlaat, Robby | 2022 | No quantification done; |
| Opportunistic Pathogens of Recreational Waters with Emphasis on Antimicrobial Resistance-A Possible Subject of Human Health Concern. | Stec, Joanna; Kosikowska, Urszula; Mendrycka, Mariola; Stepien-Pysniak, Dagmara; Niedzwiedzka-Rystwej, Paulina; Bebnowska, Dominika; Hrynkiewicz, Rafal; Zietara-Wysocka, Joanna; Grywalska, Ewelina | 2022 | Narrative Review; |
| Clinical and economic implications of urinary tract infections. | Steiger, Samantha N; Comito, Rachel R; Nicolau, David P | 2017 | No AMR Analysis; |
| Is Procalcitonin Biomarker-Guided Antibiotic Therapy a Cost-Effective Approach to Reduce Antibiotic Resistant and Clostridium difficile Infections in Hospitalized Patients? | Steuten L.; Mewes J.; Lepage-Nefkens I.; Vrijhoef H. | 2018 | No drivers/impacts identified; |
| Public health service provision by community pharmacies: a systematic map of evidence. | Stokes, G.; Rees, R.; Khatwa, M.; Stansfield, C.; Burchett, H.; Dickson, K.; Brunton, G.; Thomas, J. | 2019 | No AMR Analysis; |
| Novel detection of nasty bugs, prevention is better than cure | Strom M.; Crowley T.; Shigdar S. | 2021 | Narrative Review; |
| Antibiotic Resistance during COVID-19: A Systematic Review | Sulayyim H.J.A.; Ismail R.; Hamid A.A.; Ghafar N.A. | 2022 | No quantification done; |
| Antibiotic prescription practices in primary care in low- And middle-income countries: A systematic review and meta-analysis | Sulis G.; Adam P.; Nafade V.; Gore G.; Daniels B.; Daftary A.; Das J.; Gandra S.; Pai M. | 2020 | No AMR Analysis; |
| Exposure to World Health Organization's AWaRe antibiotics and isolation of multidrug resistant bacteria: a systematic review and meta-analysis | Sulis G.; Sayood S.; Katukoori S.; Bollam N.; George I.; Yaeger L.H.; Chavez M.A.; Tetteh E.; Yarrabelli S.; Pulcini C.; Harbarth S.; Mertz D.; Sharland M.; Moja L.; Huttner B.; Gandra S. | 2022 | No Socioeconomic Analysis; |
| Helicobacter pylori recrudescence and its influencing factors | Sun Y.; Zhang J. | 2019 | No AMR Analysis; |
| Antibiotic use and antimicrobial resistance - A review | Sushma P.G.; Venkatesh; Sasanka L.K.; Kavitha S. | 2021 | Full text not available; |
| Efficacy of 3-day versus 5-day antibiotic therapy for clinically diagnosed nonsevere pneumonia in children from developing countries. | Sutijono, Darrell; Hom, Jeffrey; Zehtabchi, Shahriar | 2011 | No drivers/impacts identified; |
| Human Colonization with Antibiotic-Resistant Bacteria from Nonoccupational Exposure to Domesticated Animals in Low- and Middle-Income Countries: A Critical Review. | Swarthout, Jenna M; Chan, Elana M G; Garcia, Denise; Nadimpalli, Maya L; Pickering, Amy J | 2022 | Full text not available; |
| Antimicrobial resistance in India | Swetha N.B.; Shivani M.; Gopalakrishnan S. | 2019 | Narrative Review; |
| A review of current knowledge on staphylococcus agnetis in poultry | Szafraniec G.M.; Szeleszczuk P.; Dolka B. | 2020 | Narrative Review; |
| A Systematic Review of the Definitions, Determinants, and Clinical Outcomes of Antimicrobial De-escalation in the Intensive Care Unit | Tabah A.; Cotta M.O.; Garnacho-Montero J.; Schouten J.; Roberts J.A.; Lipman J.; Tacey M.; Timsit J.-F.; Leone M.; Zahar J.R.; De Waele J.J. | 2016 | No Socioeconomic Analysis; |
| Epidemiology of methicillin-resistant staphylococcus aureus in arab countries of the middle east and north african (mena) region | Tabaja H.; Hindy J.-R.; Kanj S.S. | 2021 | Wrong study design; |
| Antimicrobial resistance in Africa: a systematic review | Tadesse, Birkneh Tilahun; Ashley, Elizabeth A.; Ongarello, Stefano; Havumaki, Joshua; Wijegoonewardena, Miranga; Gonz√°lez, Iveth J.; Dittrich, Sabine | 2017 | No drivers/impacts identified; |
| A Rapid Review of Environmental Health Gaps in Antimicrobial Resistance and Water-Related Research from 1990-2020. | Taing, Lina; Bhatia, Himesh; Kaiser, Rachel A; Qadir, Manzoor; Mehmood, Hamid | 2022 | No drivers/impacts identified; |
| Surveillance system of healthcare-associated infection in high and upper-middle income countries: A scoping review | Takaya S.; Matsunaga N.; Hayakawa K.; Moriyama Y.; Katanami Y.; Tajima T.; Tanaka C.; Kimura Y.; Saito S.; Kusama Y.; Morioka S.; Fujitomo Y.; Ohmagari N. | 2018 | No drivers/impacts identified; |
| Antimicrobial Stewardship | Tamma P.D.; Cosgrove S.E. | 2011 | Narrative Review; |
| Antibiotic awareness and practices among the public and patients seeking healthcare in Southeast Asia: A review | Tan M. | 2019 | Full text not available; |
| Salmonella spp. in Chicken: Prevalence, Antimicrobial Resistance, and Detection Methods | Tan S.J.; Nordin S.; Esah E.M.; Mahror N. | 2022 | Narrative Review; |
| Impact of antimicrobial stewardship program on antimicrobial-resistance and prescribing in nursing homes: a systematic review and meta-analysis. | Tandan, Meera; Thapa, Poshan; Maharjan, Preeti; Bhandari, Buna | 2022 | No Socioeconomic Analysis; |
| Antimicrobial resistance in the environment: The Indian scenario. | Taneja, Neelam; Sharma, Megha | 2019 | Narrative Review; |
| Examination of unintended consequences of antibiotic use restrictions in food-producing animals: Sub-analysis of a systematic review | Tang K.L.; Caffrey N.P.; Nobrega D.B.; Cork S.C.; Ronksley P.E.; Barkema H.W.; Polachek A.J.; Ganshorn H.; Sharma N.; Kellner J.D.; Checkley S.L.; Ghali W.A. | 2019 | Not human health; |
| Restricting the use of antibiotics in food-producing animals and its associations with antibiotic resistance in food-producing animals and human beings: a systematic review and meta-analysis | Tang K.L.; Caffrey N.P.; Nobrega D.B.; Cork S.C.; Ronksley P.E.; Barkema H.W.; Polachek A.J.; Ganshorn H.; Sharma N.; Kellner J.D.; Ghali W.A. | 2017 | No drivers/impacts identified; |
| Contemporary situation of community-acquired pneumonia in China: A systematic review | Tang X.; Zhu Y.; Lu Y.; Zhang J.; Qu J. | 2017 | No AMR Analysis; |
| Impact of antimicrobial multidrug resistance on inpatient care cost: An evaluation of the evidence | Tansarli G.S.; Karageorgopoulos D.E.; Kapaskelis A.; Falagas M.E. | 2013 | No drivers/impacts identified; |
| Multidrug resistance: an emerging crisis. | Tanwar, Jyoti; Das, Shrayanee; Fatima, Zeeshan; Hameed, Saif | 2014 | Narrative Review; |
| Awareness of inappropriate use related to antimicrobial resistance among medical doctors by country economic status: A systematic review. | Taylor, Winthrop; Whittaker, Lisa-Marie; Fletcher, Trevon; Collins, Anthony; Grant, Ryan; Gossell-Williams, Maxine | 2022 | Full text not available; |
| A systematic review of the burden of multidrug-resistant healthcare-associated infections among intensive care unit patients in southeast asia: The rise of multidrug-resistant acinetobacter baumannii | Teerawattanapong N.; Panich P.; Kulpokin D.; Na Ranong S.; Kongpakwattana K.; Saksinanon A.; Goh B.-H.; Lee L.-H.; Apisarnthanarak A.; Chaiyakunapruk N. | 2018 | No drivers/impacts identified; |
| Understanding physician antibiotic prescribing behaviour: A systematic review of qualitative studies | Teixeira Rodrigues A.; Roque F.; Falcao A.; Figueiras A.; Herdeiro M.T. | 2013 | No quantification done; |
| Risk factors for aquiring multidrug-resistant organisms in urinary tract infections: A systematic literature review | Tenney J.; Hudson N.; Alnifaidy H.; Li J.T.C.; Fung K.H. | 2018 | No quantification done; |
| Antimicrobial resistance: impact on clinical and economic outcomes and the need for new antimicrobials. | Thabit, Abrar K; Crandon, Jared L; Nicolau, David P | 2015 | Narrative Review; |
| Global travel and Gram-negative bacterial resistance; implications on clinical management. | Theriault, Nicolette; Tillotson, Glenn; Sandrock, Christian E | 2021 | No quantification done; |
| Linking Sustainable Use Policies to Novel Economic Incentives to Stimulate Antibiotic Research and Development. | Theuretzbacher, Ursula; Ardal, Christine; Harbarth, Stephan | 2017 | Narrative Review; |
| Medicine 'misuse': Implications for health and environmental sustainability. | Thomas, Felicity; Depledge, Michael | 2015 | Narrative Review; |
| Hardwiring antimicrobial resistance mitigation into global policy. | Thornber, Kelly; Kirchhelle, Claas | 2022 | Narrative Review; |
| Infection Management in Patients with Sepsis and Septic Shock in Resource-Limited Settings.Sepsis Management in Resource-limited Settings | Thwaites, C. Louise; Lundeg, Ganbold; Dondorp, Arjen M.; Adhikari, Neill K. J.; Nakibuuka, Jane; Jawa, Randeep; Mer, Mervyn; Murthy, Srinivas; Schultz, Marcus J.; Thien, Binh Nguyen; Kwizera, Arthur | 2019 | Narrative Review; |
| Burden of antimicrobial resistance in an era of decreasing susceptibility | Tillotson G.S.; Zinner S.H. | 2017 | Narrative Review; |
| Wastewater surveillance of antibiotic-resistant bacterial pathogens: A systematic review. | Tiwari, Ananda; Kurittu, Paula; Al-Mustapha, Ahmad I; Heljanko, Viivi; Johansson, Venla; Thakali, Ocean; Mishra, Shyam Kumar; Lehto, Kirsi-Maarit; Lipponen, Anssi; Oikarinen, Sami; Pitkanen, Tarja; Heikinheimo, Annamari | 2022 | Not human health; |
| Clinician-targeted interventions to influence antibiotic prescribing behaviour for acute respiratory infections in primary care: An overview of systematic reviews | Tonkin-Crine S.K.G.; Tan P.S.; van Hecke O.; Wang K.; Roberts N.W.; Mccullough A.; Hansen M.P.; Butler C.C.; Del Mar C.B. | 2017 | No drivers/impacts identified; |
| Multidrug resistant Enterobacteriaceae in New Zealand: a current perspective. | Toombs-Ruane, L J; Benschop, J; Burgess, S; Priest, P; Murdoch, D R; French, N P | 2017 | Narrative Review; |
| Antimicrobial resistance in Colombia under the scope of One Health approach | Toro-Alzate L.F. | 2021 | No drivers/impacts identified; |
| The Pandemic beyond the Pandemic: A Scoping Review on the Social Relationships between COVID-19 and Antimicrobial Resistance. | Toro-Alzate, Luisa; Hofstraat, Karlijn; de Vries, Daniel H | 2021 | No drivers/impacts identified; |
| Evidence of factors influencing self-medication with antibiotics in low and middle-income countries: a systematic scoping review | Torres N.F.; Chibi B.; Middleton L.E.; Solomon V.P.; Mashamba-Thompson T.P. | 2019 | No quantification done; |
| The use of non-prescribed antibiotics; prevalence estimates in low-and-middle-income countries. A systematic review and meta-analysis. | Torres, Neusa F; Chibi, Buyisile; Kuupiel, Desmond; Solomon, Vernon P; Mashamba-Thompson, Tivani P; Middleton, Lyn E | 2021 | No quantification done; |
| Identification of cultural determinants of antibiotic use cited in primary care in Europe: a mixed research synthesis study of integrated design "Culture is all around us" | Touboul-Lundgren P.; Jensen S.; Drai J.; Lindbaek M. | 2015 | No quantification done; |
| Hospital antimicrobial stewardship in the nonuniversity setting. | Trivedi, Kavita K; Kuper, Kristi | 2014 | Narrative Review; |
| The effect of digital antimicrobial stewardship programmes on antimicrobial usage, length of stay, mortality and cost | Trotter N.E.; Slight S.P.; Karimi R.; Bates D.W.; Sheikh A.; Weir C.; Tolley C.L. | 2023 | No drivers/impacts identified; |
| State of the Art, Unresolved Issues, and Future Research Directions in the Fight against Hepatitis C Virus: Perspectives for Screening, Diagnostics of Resistances, and Immunization. | Trucchi, Cecilia; Orsi, Andrea; Alicino, Cristiano; Sticchi, Laura; Icardi, Giancarlo; Ansaldi, Filippo | 2016 | Narrative Review; |
| Antimicrobial Resistance (AMR) Systems Map | UK Department of Health | 2014 | Wrong study design; |
| Drivers that Increase the Burden of Infection Map | UK Department of Health | 2016 | Wrong study design; |
| Sexually transmitted infections: challenges ahead. | Unemo, Magnus; Bradshaw, Catriona S; Hocking, Jane S; de Vries, Henry J C; Francis, Suzanna C; Mabey, David; Marrazzo, Jeanne M; Sonder, Gerard J B; Schwebke, Jane R; Hoornenborg, Elske; Peeling, Rosanna W; Philip, Susan S; Low, Nicola; Fairley, Christopher K | 2017 | No AMR Analysis; |
| Gonorrhoea and gonococcal antimicrobial resistance surveillance networks in the WHO European Region, including the independent countries of the former Soviet Union. | Unemo, Magnus; Ison, Catherine A; Cole, Michelle; Spiteri, Gianfranco; van de Laar, Marita; Khotenashvili, Lali | 2013 | Narrative Review; |
| One Health approach to Clostridioides difficile in Japan | Usui M. | 2020 | Narrative Review; |
| Clinical impact of antimicrobial resistance in animals. | Vaarten, J | 2012 | Narrative Review; |
| Interplay between ESKAPE Pathogens and Immunity in Skin Infections: An Overview of the Major Determinants of Virulence and Antibiotic Resistance. | Vale de Macedo, Gustavo Henrique Rodrigues; Costa, Gabrielle Damasceno Evangelista; Oliveira, Elane Rodrigues; Damasceno, Glauciane Viera; Mendonca, Juliana Silva Pereira; Silva, Lucas Dos Santos; Chagas, Vitor Lopes; Bazan, Jose Manuel Noguera; Alianca, Amanda Silva Dos Santos; Miranda, Rita de Cassia Mendonca de; Zagmignan, Adrielle; Monteiro, Andrea de Souza; Nascimento da Silva, Luis Claudio | 2021 | Narrative Review; |
| Antibiotic use and resistance in long term care facilities. | van Buul, Laura W; van der Steen, Jenny T; Veenhuizen, Ruth B; Achterberg, Wilco P; Schellevis, Francois G; Essink, Rob T G M; van Benthem, Birgit H B; Natsch, Stephanie; Hertogh, Cees M P M | 2012 | No quantification done; |
| PIN132 HEALTH-ECONOMIC MODELLING OF INFECTIOUS DISEASE DIAGNOSTICS: CURRENT APPROACHES AND FUTURE OPPORTUNITIES | Van der Pol S.; Rojas P.; Juarez C.; van Asselt A.D.I.; Antonanzas F.; Postma M. | 2019 | Full text not available; |
| Systematic literature review of antibiotic stewardship interventions in hospitalized patients in low-and middleincome countries | Van Dijck C.; Vlieghe E.; Cox J.A. | 2017 | Narrative Review; |
| Assessment of the inclusion of vaccination as an intervention to reduce antimicrobial resistance in AMR national action plans: a global review. | van Heuvel, Lotte; Caini, Saverio; Duckers, Michel L A; Paget, John | 2022 | No drivers/impacts identified; |
| Government policy interventions to reduce human antimicrobial use: A systematic review and evidence map | Van Katwyk S.R.; Grimshaw J.M.; Nkangu M.; Nagi R.; Mendelson M.; Taljaard M.; Hoffman S.J. | 2019 | No drivers/impacts identified; |
| Prevalence and resistance of H. pylori in children and adults in Vietnam | Van Khien V.; Khanh P.H.; Nam D.V.; Ha N.T.V.; Ha D.T.; Ut N.T.; Trang T.H.; Dung H.D.Q.; Yamaoka Y. | 2021 | Wrong study design; |
| Modelling the transmission of healthcare associated infections: a systematic review. | van Kleef, Esther; Robotham, Julie V; Jit, Mark; Deeny, Sarah R; Edmunds, William J | 2013 | No drivers/impacts identified; |
| Antibiotic Use in Food Animals in the World with Focus on Africa: Pluses and Minuses | Van T.T.H.; Yidana Z.; Smooker P.M.; Coloe P.J. | 2019 | Narrative Review; |
| Animal Board Invited Review: Comparing conventional and organic livestock production systems on different aspects of sustainability. | van Wagenberg, C P A; de Haas, Y; Hogeveen, H; van Krimpen, M M; Meuwissen, M P M; van Middelaar, C E; Rodenburg, T B | 2017 | Not human health; |
| Antimicrobial resistance and prudent drug use for Streptococcus suis. | Varela, Norma P; Gadbois, Pierre; Thibault, Claude; Gottschalk, Marcelo; Dick, Paul; Wilson, Jeff | 2013 | Narrative Review; |
| Antimicrobial susceptibility profile & resistance mechanisms of Global Antimicrobial Resistance Surveillance System (GLASS) priority pathogens from India | Veeraraghavan B.; Walia K. | 2019 | Narrative Review; |
| Antimicrobial resistance in the food chain: A review | Verraes C.; Van Boxstael S.; Van Meervenne E.; Van Coillie E.; Butaye P.; Catry B.; de Schaetzen M.-A.; Van Huffel X.; Imberechts H.; Dierick K.; Daube G.; Saegerman C.; De Block J.; Dewulf J.; Herman L. | 2013 | Narrative Review; |
| Review of health economic models for antibiotics | Virhage M.; Polyzoi M.; Geale K.; Corcoran K.; Anell B. | 2016 | Full text not available; |
| Microbiology laboratories involved in disease and antimicrobial resistance surveillance: Strengths and challenges of the central African states. | Vounba, Passoret; Loul, Severin; Tamadea, Ludovic F; Siawaya, Joel F D | 2022 | Narrative Review; |
| Antimicrobial resistance and biological governance: explanations for policy failure. | Wallinga, D; Rayner, G; Lang, T | 2015 | Narrative Review; |
| Shifts in Geographic Distribution and Antimicrobial Resistance during a Prolonged Typhoid Fever Outbreak - Bundibugyo and Kasese Districts, Uganda, 2009-2011 | Walters M.S.; Routh J.; Mikoleit M.; Kadivane S.; Ouma C.; Mubiru D.; Mbusa B.; Murangi A.; Ejoku E.; Rwantangle A.; Kule U.; Lule J.; Garrett N.; Halpin J.; Maxwell N.; Kagirita A.; Mulabya F.; Makumbi I.; Freeman M.; Joyce K.; Hill V.; Downing R.; Mintz E. | 2014 | Wrong study design; |
| Efficacy of antibiotic prophylaxis in children with vesicoureteral reflux: Systematic review and meta-analysis | Wang H.-H.S.; Gbadegesin R.A.; Foreman J.W.; Nagaraj S.K.; Wigfall D.R.; Wiener J.S.; Routh J.C. | 2015 | No Socioeconomic Analysis; |
| Literature review on the distribution characteristics and antimicrobial resistance of bacterial pathogens in neonatal sepsis | Wang J.; Zhang H.; Yan J.; Zhang T. | 2022 | No Socioeconomic Analysis; |
| Antimicrobial resistance in enterococcus faecalis and enterococcus faecium in children hospitals in China: A meta-analysis | Wang J.; Zhou M.; Liu F.; Lee Y.F. | 2021 | Full text not available; |
| Prevalence and serotype distribution of nasopharyngeal carriage of Streptococcus pneumoniae in China: A meta-analysis | Wang L.; Fu J.; Liang Z.; Chen J. | 2017 | No AMR Analysis; |
| Inventory of antibiotic stewardship programs in general practice in France and abroad. | Wang, S; Pulcini, C; Rabaud, C; Boivin, J-M; Birge, J | 2015 | No drivers/impacts identified; |
| Determinants of in-hospital antibiotic prescription behaviour: a systematic review and formation of a comprehensive framework. | Warreman, E. B.; Lambregts, M. M. C.; Wouters, R. H. P.; Visser, L. G.; Staats, H.; Dijk, E. van; Boer, M. G. J. de | 2019 | No quantification done; |
| Landscape of Push Funding in Antibiotic Research: Current Status and Way Forward. | Wasan, Himika; Singh, Devendra; Reeta, K H; Gupta, Yogendra Kumar | 2023 | Narrative Review; |
| Tackling antibiotic resistance in India | Wattal C.; Goel N. | 2014 | Narrative Review; |
| Theory-based explanation as intervention. | Weisman, Kara; Markman, Ellen M | 2017 | Narrative Review; |
| A Pandemic Instrument Can Start Turning Collective Problems into Collective Solutions by Governing the Common-Pool Resource of Antimicrobial Effectiveness | Weldon, Isaac; Liddell, Kathy; Katwyk, Susan Rogers Van; Hoffman, Steven J.; Minssen, Timo; Outterson, Kevin; Palmer, Stephanie; Viens, A. M.; Vi√±uales, Jorge | 2022 | Narrative Review; |
| Clinical and economic burden of community-acquired pneumonia among adults in Europe. | Welte, T; Torres, A; Nathwani, D | 2012 | No drivers/impacts identified; |
| Gram-negative neonatal sepsis in low- And lower-middle-income countries and WHO empirical antibiotic recommendations: A systematic review and meta-analysis | Wen S.C.H.; Ezure Y.; Rolley L.; Spurling G.; Lau C.L.; Riaz S.; Paterson D.L.; Irwin A.D. | 2021 | No drivers/impacts identified; |
| Is antimicrobial susceptibility testing necessary before first-line treatment for Helicobacter pylori infection? - Meta-analysis of randomized controlled trials | Wenzhen Y.; Yumin L.; Quanlin G.; Kehu Y.; Lei J.; Donghai W.; Lijuan Y. | 2010 | No drivers/impacts identified; |
| Bacteriophage therapy to combat bacterial infections in poultry. | Wernicki, Andrzej; Nowaczek, Anna; Urban-Chmiel, Renata | 2017 | Narrative Review; |
| Clinical Factors Implicated in Antibiotic Resistance in Helicobacter pylori Patients. | White, Brian; Winte, Maria; DeSipio, Joshua; Phadtare, Sangita | 2022 | Narrative Review; |
| Role of Physiology, Immunity, Microbiota, and Infectious Diseases in the Gut Health of Poultry. | Wickramasuriya, Samiru S; Park, Inkyung; Lee, Kyungwoo; Lee, Youngsub; Kim, Woo H; Nam, Hyoyoun; Lillehoj, Hyun S | 2022 | No AMR Analysis; |
| Self-medication and self-treatment with short-term antibiotics in asian countries: A literature review | Widowati I.G.A.R.; Budayanti N.N.S.; Januraga P.P.; Duarsa D.P. | 2021 | No quantification done; |
| Methicillin-resistant staphylococci (MRS) and extended-spectrum beta-lactamases (ESBL)-producing Enterobacteriaceae in companion animals: nosocomial infections as one reason for the rising prevalence of these potential zoonotic pathogens in clinical sampl | Wieler, Lothar H; Ewers, Christa; Guenther, Sebastian; Walther, Birgit; Lubke-Becker, Antina | 2011 | Narrative Review; |
| Food Safety: at the center of a One Health approach for combating zoonoses. | Wielinga, Peter R; Schlundt, Jorgen | 2013 | Narrative Review; |
| Multiresistant bacteria and current therapy - the economical side of the story. | Wilke, Michael H | 2010 | No drivers/impacts identified; |
| Interventions to reduce antibiotic prescribing in LMICs: A scoping review of evidence from human and animal health systems | Wilkinson A.; Ebata A.; Macgregor H. | 2019 | No drivers/impacts identified; |
| A review of national action plans on antimicrobial resistance: strengths and weaknesses. | Willemsen, Angela; Reid, Simon; Assefa, Yibeltal | 2022 | No quantification done; |
| Interventions for primary vesicoureteric reflux | Williams G.; Hodson E.M.; Craig J.C. | 2019 | No Socioeconomic Analysis; |
| Novel strategies for prevention and treatment of antimicrobial resistance in sexually-transmitted infections. | Williams, Eloise; Fairley, Christopher K; Williamson, Deborah | 2021 | Full text not available; |
| Helicobacter pylori infection: a review of current scientific research on the efficacy or potential of herbal medicine for the treatment of H. pylori infection of the gastric mucosa. | Williams, L. | 2011 | No drivers/impacts identified; |
| Lessons learned from COVID-19 for the post-antibiotic future | Wilson L.A.; Rogers Van Katwyk S.; Fafard P.; Viens A.M.; Hoffman S.J. | 2020 | Narrative Review; |
| Analysing policy interventions to prohibit over-the-counter antibiotic sales in four Latin American countries | Wirtz V.J.; Herrera-Patino J.J.; Santa-Ana-Tellez Y.; Dreser A.; Elseviers M.; Vander Stichele R.H. | 2013 | No drivers/impacts identified; |
| Prevalence and risk factors of community-associated methicillin-resistant Staphylococcus aureus carriage in Asia-Pacific region from 2000 to 2016: a systematic review and meta-analysis. | Wong, Jonathan Wh; Ip, Margaret; Tang, Arthur; Wei, Vivian Wi; Wong, Samuel Ys; Riley, Steven; Read, Jonathan M; Kwok, Kin On | 2018 | No drivers/impacts identified; |
| Approaches to multidrug-resistant organism prevention and control in long-term care facilities for older people: a systematic review and meta-analysis. | Wong, Valerie Wing Yu; Huang, Ying; Wei, Wan In; Wong, Samuel Yeung Shan; Kwok, Kin On | 2022 | No drivers/impacts identified; |
| Tackling antimicrobial resistance (AMR) together: working paper 5.0: enhancing the focus on gender and equity | World Health Organization | 2018 | Narrative Review; |
| Using the best available data to estimate the cost of antimicrobial resistance: A systematic review | Wozniak T.M.; Barnsbee L.; Lee X.J.; Pacella R.E. | 2019 | No drivers/impacts identified; |
| Review of the epidemiological data regarding antimicrobial resistance in Gram-negative bacteria in Australia | Wozniak T.M.; Paterson D.; Halton K. | 2017 | No drivers/impacts identified; |
| Potential negative effects of antimicrobial allergy labelling on patient care: A systematic review | Wu J.H.-C.; Langford B.J.; Schwartz K.L.; Zvonar R.; Raybardhan S.; Leung V.; Garber G. | 2018 | No drivers/impacts identified; |
| Barriers and facilitators of implementing interventions to improve appropriate antibiotic use in low- and middle-income countries: a systematic review based on the Consolidated Framework for Implementation Research. | Wu, Shishi; Tannous, Elias; Haldane, Victoria; Ellen, Moriah E; Wei, Xiaolin | 2022 | No quantification done; |
| Economic evaluation of antimicrobial stewardship in primary care: a systematic review and quality assessment | Wubishet B.L.; Merlo G.; Ghahreman-Falconer N.; Hall L.; Comans T. | 2022 | No drivers/impacts identified; |
| Epidemiology of carbapenem resistant Enterobacteriaceae (CRE) during 2000-2012 in Asia | Xu Y.; Gu B.; Huang M.; Liu H.; Xu T.; Xia W.; Wang T. | 2015 | No drivers/impacts identified; |
| Economic burden of antimicrobial resistance in low and middle-income countries: A systematic review and expert consultation | Ya K.Z. | 2019 | Full text not available; |
| Mechanism of resistance acquisition and treatment of macrolide-resistant Mycoplasma pneumoniae pneumonia in children | Yang H.-J.; Song D.J.; Shim J.Y. | 2017 | Narrative Review; |
| A comprehensive review on quinolone contamination in environments: current research progress. | Yang, Chendong; Wu, Tianyu | 2023 | Narrative Review; |
| Situational analysis of antibiotic use and resistance in Ghana: policy and regulation. | Yevutsey, Saviour Kwame; Buabeng, Kwame Ohene; Aikins, Moses; Anto, Berko Panyin; Biritwum, Richard B; Frimodt-Moller, Niels; Gyansa-Lutterodt, Martha | 2017 | No drivers/impacts identified; |
| Antibiotic resistance amongst healthcare-associated pathogens in China. | Yezli, Saber; Li, Han | 2012 | Narrative Review; |
| The Progress of Global Antimicrobial Resistance Governance and Its Implication to China: A Review. | Yin, Jia; Wang, Yu; Xu, Xueran; Liu, Yinqi; Yao, Lu; Sun, Qiang | 2021 | No quantification done; |
| Enhancing the role of pharmacy students in appropriate use of antibiotics and preventing its resistance - a systematic review | Yokesh M.; Shanmugasundaram P. | 2021 | Full text not available; |
| Financial strategies targeting health care providers to promote the prudent use of antibiotics: a systematic review of the evidence | Yoshikawa Y.; Feldhaus I.; Ozcelik E.; Hashiguchi T.C.O.; Cecchini M. | 2021 | No drivers/impacts identified; |
| Antibiotic resistance of helicobacter pylori in Iranian children: A systematic review and meta-analysis | Yousefi-Avarvand A.; Vaez H.; Tafaghodi M.; Sahebkar A.H.; Arzanlou M.; Khademi F. | 2018 | No drivers/impacts identified; |
| Carbapenamase-Producing Acinetobacter baumannii in China, Latin America and the Caribbean: A Systematic Review and Meta-Analysis | Yu H.; Ezpeleta-Lobato G.; Han X.; Carmona-Cartaya Y.; Quinones-Perez D. | 2022 | No drivers/impacts identified; |
| Global status of antimicrobial resistance among environmental isolates of Vibrio cholerae O1/O139: a systematic review and meta-analysis. | Yuan, Xin-Hui; Li, Yu-Mei; Vaziri, Ali Zaman; Kaviar, Vahab Hassan; Jin, Yang; Jin, Yu; Maleki, Abbas; Omidi, Nazanin; Kouhsari, Ebrahim | 2022 | Not human health; |
| Recent trends in the epidemiology, diagnosis, treatment, and mechanisms of resistance in clinical Aspergillus species: A general review with a special focus on the Middle Eastern and North African region. | Zakaria, Ayate; Osman, Marwan; Dabboussi, Fouad; Rafei, Rayane; Mallat, Hassan; Papon, Nicolas; Bouchara, Jean-Philippe; Hamze, Monzer | 2020 | Narrative Review; |
| Multidrug-resistant typhoid fever: a review. | Zaki, Syed Ahmed; Karande, Sunil | 2011 | No quantification done; |
| Antibiotics and Antibiotic Resistance Genes in Animal Manure - Consequences of Its Application in Agriculture. | Zalewska, Magdalena; Blazejewska, Aleksandra; Czapko, Agnieszka; Popowska, Magdalena | 2021 | Narrative Review; |
| Human use of antimicrobial agents. | Zarb, P; Goossens, H | 2012 | Narrative Review; |
| Association Between Antimicrobial Stewardship Programs and Antibiotic Use Globally: A Systematic Review and Meta-Analysis | Zay Ya K.; Win P.T.N.; Bielicki J.; Lambiris M.; Fink G. | 2023 | No drivers/impacts identified; |
| Effects of social norm feedback on antibiotic prescribing and its characteristics in behaviour change techniques: a mixed-methods systematic review | Zeng Y.; Shi L.; Liu C.; Li W.; Li J.; Yang S.; Yang X.; Huang Q.; Yang L. | 2023 | No drivers/impacts identified; |
| Outpatient Antibiotic Use and the Need for Increased Antibiotic Stewardship Efforts. | Zetts, Rachel M; Stoesz, Andrea; Smith, Brian A; Hyun, David Y | 2018 | Narrative Review; |
| Situation analysis of antibiotic resistance of Helicobacter pylori in Asia: A systematic review | Zhang L.; Li H.; Liu C.-X.; Sun B.-Y.; Wang H.; Huang X.-M.; Lv M.-H. | 2019 | Full text not available; |
| Antibiotic Stewardship in Retail Pharmacies and the Access-Excess Challenge in China: A Policy Review | Zhang T.; Lambert H.; Zhao L.; Liu R.; Shen X.; Wang D.; Cabral C. | 2022 | No quantification done; |
| Antimicrobial Resistance of Streptococcus uberis Isolated from Bovine Mastitis: A Review | Zhang T.; Tao L.; Boonyayatra S.; Niu G. | 2022 | Narrative Review; |
| Infection Prevalence and Antibiotic Resistance Levels in Ureaplasma urealyticum and Mycoplasma hominis in Gynecological Outpatients of a Tertiary Hospital in China from 2015 to 2018 | Zhang W.; Li L.; Zhang X.; Fang H.; Chen H.; Rong C. | 2021 | Wrong study design; |
| Systematic review of the economic burden of antibiotic resistance | Zhen X.; Hu X.; Dong H. | 2018 | Full text not available; |
| Economic burden of antibiotic resistance in ESKAPE organisms: A systematic review | Zhen X.; Lundborg C.S.; Sun X.; Hu X.; Dong H. | 2019 | No drivers/impacts identified; |
| The Clinical and Economic Impact of Antibiotic Resistance in China: A Systematic Review and Meta-Analysis. | Zhen, Xuemei; Lundborg, Cecilia Stalsby; Sun, Xueshan; Hu, Xiaoqian; Dong, Hengjin | 2019 | No drivers/impacts identified; |
| A systematic review of antibiotics and antibiotic resistance genes in estuarine and coastal environments. | Zheng, Dongsheng; Yin, Guoyu; Liu, Min; Chen, Cheng; Jiang, Yinghui; Hou, Lijun; Zheng, Yanling | 2021 | No drivers/impacts identified; |
| Insects represent a link between food animal farms and the urban environment for antibiotic resistance traits. | Zurek, Ludek; Ghosh, Anuradha | 2014 | Narrative Review; |
| Antimicrobial resistance: global report on surveillance. |  | 2014 | Narrative Review; |
| Global burden of bacterial antimicrobial resistance in 2019: a systematic analysis |  | 2022 | No drivers/impacts identified; |

Supplementary Table 6: Detailed list of drivers

| **Dimension** | **Theme** | **Risk Factor** | **Protective Factor** |
| --- | --- | --- | --- |
| People & Public | Age | Being a child (3,6–8,20,21)  Being middle age (20)  Being of older age (8,16,20) | Being of middle age (20)  Being of older age (16,20) |
|  | Sex and Gender | Being male (2,8,11,20,23)  Being female (8,11,20)  Being a female carer (11,20) | Being male (19,20)  Being a male carer (20)  Being female (19,20)  Being a female carer (20) |
|  | Ethnicity | Being African-American compared to Caucasian (8,9)  Being First Nations compared to Caucasian (17)  Being White compared to POC (8) |  |
|  | Migrant Status | Immigration (all) (8)  Recent immigration (11,18,20)  Forcible displacement (18)  Living in a high-migrant community setting (18)  Being born on Indian subcontinent (8) | Immigration (all) (20) |
|  | Marginalisation | Being Intravenous Drug User (IVDU) (1,8)  Current homelessness (1,8)  Previous homelessness (8)  Working or residing in prison (8) |  |
|  | Sexual Orientation | Being MSM (8) |  |
|  | Socioeconomic Status | Low Socio-Economic Status (1,3)  Low income (8)  High income (20) | High income (20)  High Socio-Economic Status (3) |
|  | Educational Attainment | Being a university student (13)  Higher parental education (3) (8) (20) | Having a university education (20)  Higher parental education (3) (20) |
|  | Household Composition | Having no children (8,20)  Child having no siblings (8)  Having any children in the household (21)  Having older children vs younger children (11)  Having multiple children (3,11,20)  Having >5 household members (14)  Having >8 household member (1,8)  Having any household member with AMR (14)  Room-sharing with a colonised person (14) | Having a grandparent as the caregiver (20) |
|  | Maternity Factors | Breastfeeding (14)  Delivery (14)  Mother-to-child/child-to-mother transmission (14) | Breastfeeding (6,14,21) |
|  | Personal Hygiene | Sharing hygiene equipment (14)  Child wearing nappies (8) | Daily bathing/showering (14)  Antibacterial use (14) |
|  | Lifestyle Behaviours | Smoking (8)  Contact sport (14)  Sauna Use (8)  Not using condoms (8) | Smoking (21) |
| System & Environment | Household Transmission | Having a household member working in healthcare (11,14,20,21)  Having a household member working with animals (14) | Having a household member working in healthcare (20)  Having no household member in healthcare (14) |
|  | Healthcare Occupation | Working in healthcare (20)  Parents working in healthcare (3,20)  Treating infected patients (14)  Assisting infected person with grooming (14)  Being a medical student (11,20) | Parents not working in healthcare (3)  Parents working in healthcare (20)  Being a medical student (20) |
|  | Urbanicity | Living in a rural location (3,7,8,11,13,20)  Living in an urban location (8,13,20,21) | Living in a rural location (20)  Living in an urban location (20) |
|  | Day-care Attendance | Having children in day-care (6,8,21)  Using public day care vs private day care (8) |  |
|  | Environmental Hygiene | Human transmitted fomites in home environment (14)  Human transmitted fomites in clinical environment (14) |  |
|  | Regional poverty | Low regional income per capita (1)  Living in deprived area (1) |  |
|  | Recreational and Medical Tourism | Recreational travel (non-specific) (8,14)  Recreational travel to multiple regions  (8,14)  Recreational travel to Asia (all) (4,8,14)  Recreational travel to Eastern Mediterranean / Middle East  (8,14)  Recreational travel to Africa (all) (8,14)  Recreational travel to Latin America / Central & South America (4,8,14)  Recreational travel to Southern Europe  (8,14)  Travel and STDs (12)  Mass pilgrimages (8)  Eating food with locals while travelling (14)  Medical travel (any) (4,14)  Antibiotic use while travelling (22) |  |
|  | Farming and Animal Husbandry | Any contact with animals (14,21)  Contact with swine/pigs (8,14,21)  Contact with cattle  (8,14)  Contact with poultry  (8,14,21)  Contact with mink (14)  Contact with hog (14)  Contact with horses (14)  Contact with goats (14)  Contact with pets (8)  Working as a veterinarian (14)  Working in agriculture (non-specific) (14)  Working in a pigsty (14)  Contact with manure (14)  Visiting farms regularly (14) | Contact with swine/pigs (14)  Contact with cattle (14)  Contact with pets (14)  Working as a veterinarian (14)  Working on a small farm (vs big farm) (14) |
|  | Food Supply Chain | Working in a slaughterhouse  (8,14)  Handling raw meat regularly (8,14)  Being a foodhandler (14)  Working in food distribution (14)  Eating pork (14)  Eating dried poultry (14)  Drinking raw milk (14)  Giving food-producing animals antibiotics (8) |  |
|  | Water Contamination | Drinking water from wild sources (8,14)  Recreational bathing in wild waters (14)  Sharing water sources with livestock (14) |  |
|  | Climate | Living in tropical vs non-tropical climates (5) |  |
| Institution & Policies | Poor Antibiotic Quality | Using expired antibiotics (10) |  |
|  | Healthcare Financing | Having child health insurance (3,11)  Using an out-of-pocket health expenditure (8) | Having child health insurance (3) |
|  | Healthcare Governance | Lack of licensed pharmacist in pharmacy (11)  Health facility type (11)  Private sector vs public sector primary care (11) |  |
|  | National Income | Lower national income level (5,15,20)  Higher national income level (16) |  |

Supplementary Table 7: Full quantitative data extraction table

| **People & Public** | **Age** | Antibiotic Self-Medication (ASM) in children:  - Prevalence: children vs adults (60% vs 47%) (p<0.0001) (7)  - OR: 1.3 (1.1-1.4), OR 1.5 (1.04-1.27), OR 1.82 (1.15-3.02), OR 2.19 (1.4-3.6) (3), OR 1.62 (1.54-1.71), OR 1.9 (1.77-2.03) (20)    AMR in children (all organisms)  - Prevalence: MRSA 18% (11-26) in children vs non-children 13% (9-18) (21)  - Prevalence: H Pylori resistance in children 40% vs adults 17.5% (8)  - MRSA: OR 3.54 (2.38-5.26), OR 2.24 (1.73–2.9) (21), OR 2.74 (1.75-4.29) (6), OR 2.03 (1.24-3.31), OR 2.24 (1.73 - 2.9), OR 9.63 (1.37-67.48) (8)  - AMR, various organisms: OR 2 (1-4), OR 1 (1-1.1), OR 2.2 (1.1 - 4.8), 1.8 (1.3-2.4), 1.5 (1.1-2), 1.3 (1-1.8), OR 14.95 (3.24-68.8), OR 7.45 (2.11-26.28), OR 7.09 (1.71-29.31), OR 2.07 (1.77-2.41), OR 1.95 (1.66-2.28), OR 1.39 (1.13-1.71) (8), 1.62 (1.54-1.71), 1.9 (1.77-2.03), 1.146 (1.037-1.266), OR 1.05 (1.04-1.07), OR 1.02 (1.01-1.04), OR 1.08 (1.02-1.14) (20)    ASM and middle-age: 21-30 (compared to 16-20) OR 1.5 (1.00-2.2) (20)    ASM and older age:  - worldwide OR 1.1 (1.02-1.18), mostly driven by data from LMICs where OR 1.12 (1.02-1.23), OR 1.25 (1.12-1.38) (20)  - ASM compared to <30: 30-40 OR 0.76 (0.76-3.03), 40-50 OR 0.23 (0.07-0.78), 50-60 OR 0.01 (0.00-0.04), >60 OR 0.07 (0.02-0.22) (20)    AMR in older age:  - MRSA: OR 0.097 (0.002-0.192) (16)  - MRSA: OR 2.03 (1.24-3.31), OR 1.09 (1.02-1.16), OR 3.51 (1.38-8.91) (8)  - AMR, various organisms: OR 6.98 (2.45 - 19.89), 3.18 (1.19 - 8.48), 3.62 (1.45 - 9.04), 2.37 (1.28 - 4.39), 7.78 (3.78 - 16.02), 2.89 (1.55-5.38),  OR 1.49 (1.02-2.16), OR 1.6 (1.08-2.47), OR 3.5 (1.1-7.4), OR 3.1 (1.1-8.7), OR 2.8 (1.1-7.4), OR 2.4 (1.1-5.49), OR 1.67 (1.23-2.26) (8),    Antibiotic Storage and older age:  - OR in HICs 0.92 (0.88-0.97). OR in LMICs 1.04 (1.01-1.07) (20) |
| --- | --- | --- |
|  | **Sex and Gender** | Male, younger than 40, primary education or less, low or middle income, Traditional Chinese Medicine user, found less likely to complete an antibiotic course: aOR 0.67 (11)    AMR and males:  - OR 1.41 (1.21-1.64) (23), OR 1.40 (1.09-1.80) (2),OR  2.2 (1.4-3.3), aOR 14.5 (2.1-100.1), OR 2.284(1.213-4.301), OR 2.7 (1.3-5.8), OR 3.8 (1.4-10.2), OR 3 (1.3-6.8), OR 2.4 (1.1-5.4), OR 3.1 (1.2-7.8), OR 1.33 (1.14-1.56), OR 2.03 (1.21-3.39), OR 3.1 (2.3-4.1), 1.59 (1.05-2.41), OR 2.44 (1.04-5.72), OR 2.1 (1.2-3.7), aOR  2.59(1.3-5.14), OR 2.1 (1.3-3.6), OR 3.6 (1.2-11), OR 1.9 (1.3-2.7) (8)  - RR 2.61 (1.08-6.28) (8)    Knowledge of AMR and males:  - male farmers compared to female farmers: 74% vs 39% (19)    ASM and males:  - OR 1.65 (1.12-2.43), OR 1.81 (1.01-3.26), OR 4.54, OR 0.7 (0.61-0.8) (20)  - Hofstede’s model of cultural dimension of masculinity : r=0.81 (20)  - Being a male carer: OR 0.1257 (20)    AMR and females:  - H Pylori: OR 3.28 (1.07-10.05), 2.78 (1.23-6.26) (8)  - various organisms: OR 5.5 (1.1-27.1), OR 3.2 (1.5-6.9), aOR 2.1 (1.14-4.04), aOR 4.49 (1.49-13.51), RR 4.3 (3.1-6.1) (8), OR 4.54 (p=0.004) (20)  - MRSA: OR 2.52 (1.2-5.3), 3.6 (1.03-11.37), 3 (3-1.3) (8)    ASM and females:  - OR: 1.12 (1.02-1.23), OR 1.44 (1.01-2.05), OR 1.43 (1.04-1.98), OR 1.47 (1.35-1.59), OR 1.2 (1.04-1.56) (20)  - being a female carer: aOR 1.25, (11,20), OR 1.22 (1.08-1.39), OR 1.17 (1.06-1.29), OR 0.83 (0.74-0.94) (20)  - being a female child: OR 0.92 (0.96- 0.99) (20)    Knowledge of antibiotic use and females:  - OR 1.18 of obtaining information on appropriate antibiotic use compared to males (in higher SES and education levels) (19) |
|  | **Ethnicity** | AMR and race:  - African-Americans vs Caucasian Americans: incidence: 2.59 vs 0.7 per 100,000 person-year  (9), aOR 1.8 (1.1-2.94), OR 1.91 (1.28-2.87), OR 2.5 (1.25-5.013), OR 13.4 (3.3-53.8), MRSA colonisation 4.7% vs 1.2% (8)  - MRSA in First Nations (Canada): prevalence: 14.78%  (17)  - MRSA in Whites vs non-whites: 3.2 (2-5.2) (8) |
|  | **Migrant Status** | ASM in immigrants vs local-born:  - in Hong Kong: aOR 2.37 (1.28 - 4.15) among recent immigrants (11)  - compared to local-born: new immigrants: OR 2.205 (1.203-3.953), OR 2.37 (1.28-4.15), 2.49 (1.385-4.477), OR 0.407 (20)  - local-born compared to immigrant: OR 0.407 (20)  - in Qatar: OR 0.29 (0.11-0.75) among non-arabs vs Qataris (20)  - compared to local-born: all immigrants 0.601 (0.436-0.829) (20)    Antibiotic storage among recent immigrants:  - in Hong Kong aOR 2.37 (1.29, 4.15) (11)    AMR (carriage or infection) among migrants (all):  - prevalence, any AMR: 25.4% (19.1–31.8) (18)  - prevalence, MRSA: 7.8% (4.8–10.7) (18)  - prevalence, Gram-Negative Bacteria: 27.2% (17.6–36.8) (18)  - H Pylori in Spanish natives vs non-natives : 33.7% vs 14.3%  (8)  - MRSA in non-Danish vs Danish patients: OR 30. 5 (3.6-257.3) (8)  - ESBL in patients born on Indian sub-continent: OR 11.13 (2.17-56.98) (8)    AMR (carriage or infection) in Forcibly Displaced Populations:  - prevalence, any AMR: 33.0% (18.3–47.6). Compared to other migrants: 6.6% (1.8-11.3) (18)  - prevalence, MRSA: 8.2% (5.0–11.3). Compared to other migrants: 6% (1.3-10.7) (18)    AMR (carriage or infections) in high-migrant community settings vs hospital settings:  - prevalence, any AMR: 33.1% (11.1-55.1) vs 24.3% (16.1-32.6) (18)  - prevalence, MRSA: 9.8% (0.0-20.3) vs 7.4% (4.2-10.6) (18)    PVL-MRSA and migrants:  - prevalence: migrants account for 62.7% (50.2-75.3) of all PVL-MRSA cases reported (18) |
|  | **Marginalisation** | MRSA in IVDU:  - in USA: OR 1.8 (1), OR 2.61 (1.1-6.15), aOR 2.11 (8)  - Prevalence in Canada: 7.4% (2000), 18.6% (2006) (1)    MRSA in homelessness:  - OR: 1.5 (1), OR 26.13, OR 2.04 (1.09-3.82) (8)  - Previous homelessness: OR 3.32 (1.27-8.7) (8)    MRSA and residence or work in prison:  - aOR 2.9 (1-7.7) (8)  - OR 1.92 (1-3.67) (8) |
|  | **Sexual Behaviours** | AMR and MSM:  - being MSM and/or IVDU in context of HIV positive: Hazard Ratio 5 (1.5-16.3) (8)  - Being MSM and having MDR Sexually Transmitted Illness: aOR 23.9 (2.2-261) (8) |
|  | **Socioeconomic Status** | AMR and low SES:  - Carbapenem-resistant A Baumannii in Israel: OR 2.18 (1.02-5) (1)  - Prevalence of AMR in children in Brazilian slums vs private schools: 65% vs 16%  (1)    ASM and low SES:  - OR 4.44 (1.52-18.95), 3.6 (1.3-9.7), OR 2 (1.1-3.8) (3)    AMR and low income:  - OR 8.9 (1.8-43.9) (8)    ABU and high income:  - OR 0.65 (0.46-0.91), OR 0.75 (0.65-0.86), 0.86 (0.77-0.96), OR 0.88 (0.79-0.99), OR 1.71 (1.5-1.94), OR 2.18 (1.29-3.68), 1.3 (1.1-1.53), 1.15 (1.04-1.27), 1.36 (1.16-1.6) (20)  - Antibiotic storage: OR 0.88 (0.79-0.99) (20)    ASM and high SES:  - In China: OR 0.66 (0.5-1) (3) |
|  | **Educational Attainment** | Higher parental education:  - ABU in UMICs: 0.75 (0.57-0.98) (20)  - ASM OR: 1.37 (1.19 - 1.57), OR: 0.71 (0.5-1), 0.34 (0.2-0.5) (3,20), OR 0.75 (0.57-0.98), OR 0.82 (0.79-0.86), OR 0.6 (0.55-0.66), OR 0.85 (0.73-0.99), OR 0.7 (0.49-0.99), OR 0.46 (0.27-0.78), OR 0.6 (0.55-0.66), OR 1.94 (1.35-2.8), OR 1.6 (1.1-2.3), 2.03 (1.53-2.69), 1.79 (1.51-2.13), 1.5 (1.51-2.13), 1.39 (1.01-1.91), 1.55 (1.33-1.81), 2.47 (1.38-4.44) (20)  - AMR prevalence: OR 2.99 (1.1-8.07) (8)    AMR and education level:  - OR: 2.43 (1.21-4.85), 2.245 (1.297-3.884) (8)    ABU and students:  - Prevalence of demanding antibiotics: 23% (13-34) (13)  - Prevalence of OTC antibiotics: 49% (35-62) (13)  - Prevalence of antibiotic non-adherence: 43% (27-61) (13)  - Prevalence of prophylactic antibiotic use: 34% (25-44) (13) |
|  | **Household Composition** | AMR and children in household (any):  - (<7 years old) OR: 2.23 (1.53-3.29) (21)    AMR and older child vs younger child:  - In China: aOR 1.15 (11)    SMA and multiple children:  - In China(>1): OR 2.17 (1.48–3.18) (3,11,20)  - In Saudi (>2): OR 1.68 (0.99–2.85) (3)  - in Western Pacific: (>1): OR 2.174 (1.485-3.183) (20)    AMR and less children:  - Having no offspring: OR 8.6 (2.2-33) (8), OR 2.428 (1.477-3.991), OR 11 (2.8-43.5) (20)  - Child with no siblings: OR 2.1 (1.3-3.4) (8)    Caregiver is grandparent:  - OR 0.68 (0.49-0.94) (20)    AMR and overcrowded household:  - in Indonesia (>8 household members): OR: 4.5 (1.4-15.1) (1,8)  - >5 household members: OR 6.63 (1,19-36,80) (14)    AMR and room-sharing with colonised person:  - OR 1.5 (1.1-2.2), OR 3 (1.43-6.31), OR 3.09 (1,52-6,23) (14)  - Risks: 5.6% (1/18) (14)    AMR and having any household member with known AMR:  - OR 9.37 (1.12-78.05), OR 13.3 (1.3-172.6), OR 7.5 (2.7-20.7), OR 6.9 (3.1-15.5), OR 3.5 (1,5-11,1), OR 2.59 (1,65-4,07), OR 12 (4 to 37), OR 1.87 (1,03-3,4) (14)  - PR 3.4 (1.3-9.0), 1.8 (1.3-2.5) (14)  - Risks: 31.37% (16/51) (14) |
|  | **Maternity Factors** | AMR and breastfeeding:  - OR 0.69 (0.52-0.93), OR: 0.65 (0.53-0.8) (14,21)  - OR 5.81 (1.65-20.44), 49.17 (6.02-354.68) (14)  - against MRSA carriage (2- to 6-month olds): OR: 0.69 (0.60-0.81) (6)  - Risks: 100% (mother’s milk to child) (4/4) (14)    AMR and delivery:  - OR 7.42 (2.06-26.71), OR 1.81 (1.12-2.93), OR 2.2 (1.1-4.3), OR 5.1 (1.8-13.7) (14)  - Risks: 88.4% (61/69), 42.1% (8/19), 15.2% (7/46), 13.8% (30/217), 14% (8/59), 54.2% (26/48)60% (12/20), 5.6% (16/318), 33% (2/6) (14)    AMR transmission risk Mother-to-child or child-to-mother:  - Risks: 2.1% (6/311), 14.29% (7/49), 11.2%, 25% (1/4), 20.6% (55/267), 19.9% (53/267) (14) |
|  | **Personal Hygiene** | AMR and sharing hygiene equipment:  - Sharing soap: OR 5.6 (1.95-16.03) (14)  - Sharing washcloth: OR 2.14 (1.08-4.22) (14)  - Sharing lotion/balm OR 1.95 (1.18-3.22) (14)  - Risks (sharing towels): 60% (3/5) (14)    AMR and daily bathing/showering:  - OR: OR 0.7 (0.6-0.98), OR 0.34 (0.16-0.84) (14)    AMR and antibacterial soap use:  - OR: OR 0.44 (0.24-0.78), 0.7 (0.5-0.97)(14)    AMR and nappy-wearing child:  - PR 1.33 (1.01-1.75) (8) |
|  | **Lifestyle Behaviours** | AMR and smoking:  - (yes vs no) OR: 0.44 (0.24-0.82)(21)  - (heavy vs non) OR 14.4 (8)    AMR and contact sports:  - OR 2.36 (1.13-4.93) (14)    AMR and sauna use:  - OR 19.1 (2.7-206.1) (8)    AMR and no condom use:  - aOR 4.3 (1.7-10.4) (8) |
| **System & Environment** | **Household Transmission** | AMR and having a household member working in healthcare:  - OR: 8.98 (1.4–55.63) (21), OR 4.7 (1-20.6), OR 3.74 (1.467-9.507) (14), OR 1.8 (1.34-2.42), OR 0.69 (0.49-0.97) (20)  - RR 3.3 (0.7-14.8) (14)  - aOR 1.38 (1.14-1.66) (11,20)  - Risks: 7.3% (10/137) (14)    AMR and having no household member working in healthcare:  - protective OR 0.19 (0.063-0.553) (14)    AMS and having a relative working in healthcare:  - Worldwide OR 1.27 (1.00-1.53). HICs OR 1.45 (0.95-1.96). LMICs OR 1.21 (0.85-1.57)  (20)    Antibiotic storage and having a relative working in healthcare:  - Worldwide OR 1.60 (1.37-1.83). HICs OR 1.80 (1.26-2.34). LMICs OR 1.56 (1.30-1.81) (20)    AMR and having a household member working with animals:  - working with swine/pigs: OR 8 (2-37) (14)  - working as a vet or with livestock (any): OR 3.7 (1.20-11.37) (14) |
|  | **Healthcare Occupation** | AMS and parents not working in medical field:  - In Jordan: correlated (p-value = 0.009) (3)    ABU and working in medical field (non-parent):  - OR 1.54 (1.35-1.75) (20)    ABU and parents working in medical field:  - AMS: OR: 2.74 (1.080-7.077), OR: 1.38 (1.14 - 1.66) (3,20), OR 3.01 (1.66-5.47), OR 0.69 (0.49-0.97), OR 0.71 (0.52-0.97), OR 1.45 (1.08-1.95), OR 1.47 (1.26-1.72), OR 0.62 (0.52-0.75), OR 1.6 (1.37-1.88), OR 0.62 (0.43-0.89), OR 0.53 (0.32-0.88), OR 1.68 (1.24-2.27), OR 1.56 (1.33-1.84) (20)    AMR and treating infected patients:  - OR 25.9 (3.1-208.8), OR 2.1 (1.1-4.0), OR 4.3 (2.0-9.1), OR 9.8 (2.0-48.9) (14)  - Risks of transmission: 2.6% (2/78), 38.7% (77/199), 2.5% (1/40), 7.7% (1/13) (14)  - Risks of contamination from glove removal: 4.5% (9/199), 8.2% (11/134), 0.75% (1/134) (14)    AMR and assisting infected person with washing/showering:  - OR 2.22 (1.34-3.69) (14)    ABU and medical students:  - Demanding antibiotics (vs non-medical students) 19% vs 12% (11)  - OR 1.612 (1.193-2.178), OR 1.18 (1.04-1.34), OR 0.84 (0.75-0.95), OR 0.52 (0.44-0.6), OR 0.69 (0.55-0.87), OR 0.49 (0.26-0.93), OR 0.71 (0.56-0.90), OR 0.64 (0.47-0.87) (20) |
|  | **Urbanicity** | ASM in rural locations:  - aOR 1.64 (11)    AMR and rural location:  - In Uganda: OR 3.7 (1.86 - 7.22) (3)  - In Tanzania (distance >30km): OR 1.2 (1.1-1.3) (3)  - In China: OR 1.6 (1.1-2.4) (3)  - OR 12.48 (1.01-153.7) (8), OR 2.01 (1.053.84), OR 0.67 (0.54-0.82), 0.64 (0.54-0.76), 0.88 (0.78-0.98) (20)    - Living in villages in Western Pacific: OR 1.643 (1.108-2.436) (20)  - In Europe: OR 2.002 (1.343-2.985) (20)    Hospital antibiotic prescription, rural vs urban  - 72% vs 66% (p=0.003) (7)    Non-adherence to prescribed antibiotic instructions among rural residents (vs urban):  - In China: 67% (48-84) vs 45 (36-55) (13)    Demanding antibiotics among urban residents (vs rural):  - In China: 48%(33-64) vs 28% (13-45) (13)    OTC antibiotic purchasing among urban residents (vs rural):  - In China: 54% (32-76) vs 32% (15-52) (13)    AMR in urban locations:  - ASM Worldwide OR: 0.79 (0.63-0.95). Mainly driven by HICs: OR 0.5 (0.3-0.7) (20)  - OR 0.65 (0.49-0.88), OR 0.79 (0.76-0.83), OR 0.69 (0.5-0.94), 0.8 (0.71-0.9), 0.56 (0.32-0.95), OR 1.495 (1.103-2.026), 1.5 (1.35-1.66), 1.6 (1.2-1.9), OR 1.643 (1.108-2.436) (20)  - Prevalence: in Northern Taiwan (urban): OR 1.45 (1.19–1.77) (8,21)  - RR 2.2 (1.4-3.6) (8)  - antibiotic storage : OR 0.8 (0.71-0.9) (20) |
|  | **Day-care Attendance** | AMR and day-care attendance:  - OR: 1.95 (1.4–2.72) OR: 1.53 (1.2–1.95), OR 4.2 (1.74-10.12) (8,21)  - OR: 1.49 (1.17-1.91) (6)  - Public day care OR 3.1 (1.6-6), OR 3.8 (2.4-6) compared to private day care: OR 2.4 (1.3-4.4), OR 1.7 (1.2-2.4) (8) |
|  | **Environmental Hygiene** | Risks of finding human transmitted AMR fomites (home environment):  - House/room (general): 51% (20/39), 68% (65/95) (14)  - Keyboard: 36% (4/11) (14)  - Faucet/sink: 36% (4/11), 7.87% (7/89) (14)  - Curtains: 33% (1/3) (14)  - Bedside cupboard: 17.33% (13/75) (14)    Risks of finding human transmitted AMR fomites (clinical environment):  - Suction with vacuum: 100% (2/2) (14)  - Ventilation filter: 100% (3/3) (14)  - Ambu-Bags: 100% (3/3) (14)  - Bedrails: 44/9% (3/7) (14)  - healthcare surfaces: 10.6% (16/151), 36%, 58%, 48%, 425, 45%, 2.1% (11/513) (14)  - healthcare clothes (gown, gloves): 20%, 4%, 30%, 6.2% (5/81), 4.3% (4/94) (14)  - clinical gloves: 17.52% (14/79), 7.7% (7/91) (14)  - bed chart: 6.45% (2/31) (14) |
|  | **Regional Poverty** | Antibiotic use and regional per capita income:  - in Sweden: r = 0.597, p<0.05 (1)    AMR and living in deprived areas:  - In UK: OR 1.33 (1.07-1.75), OR 2.47 (1.08-5.66) (1) |
|  | **Recreational and Medical Tourism** | AMR and travel to multiple regions:  - OR 6.24 (1.45-26.86), OR 2.91 (1,73-4,76), OR 10.6 (3.0-67.5),  PR 1.92 (1.24-2.96), OR 1.69 (1,17-2,43)(14), 3.089 (1.29-7.38) (8)  - Risks: 5.9% (1/17), 28.6% (12/36) (14)    AMR and travel to South-East Asia:  - OR 61.67 (5.82-653.13), OR 100 (3.34-2997.80), OR 4.94 (1.80-13.6) (14)  - Risks: 34% (37/110), 37% (200/540), 32.4% (22/46), 87.1% (27/31) (14)    AMR and Travel to East Asia:  - Risks: 67% (22/33) (14)    AMR and travel to South Asia (incl India):  - OR 2 (1.2-3.4), OR 4.09 (1.87-8.98), OR 5.3 (2.4-11.8), OR 13.6 (3.0-75.0), OR 19.9 (4,5-88,8), OR 24.8 (4.98-122) (14), OR 145.6 (77.7-252.1), 3.8 (1.5-9.5)  (8)  - Risks: 88% (7/8), 100% (15/15), 38% (3/8), 72% (18/25), 75.1% (136/181), 89% (16/18), 73.3% (11/15) (14)    AMR and travel to Central Asia / Western Asia:  - Risks: 30% (1/3), 42/9% (12/28) (14)    AMR and travel to Asia:  - OR 3.1 (1.1-8.9), OR 4.72 (2.11-10.55), OR 2.64 (1.71-4.07), OR 8.63 (3.42-21.7) (14)  - Risks: 32% (10/31), 15% (3/20), 48.8% (41/84), 11.76% (2/17), 12.8% (25/195), 13% (4/30)  (14)  - Asia as region of provenance of AMR enteric bacteria (as % of a sample): 35.24% (n=9725) (4)  - Asia as region of provenance of resistant Shigella spp. (as % of a sample): 24.48% (n=1697) (4)    AMR and travel to Easter Med / Middle East:  - OR 19.2 (1.0417 -353.8717), OR 20.56 (1.1873-355.8783), OR 75.3 (13.0-434.5) (14), OR 18.1 (8.1-35.2) (8)  - Risks: 29% (4/14), 50% (1/2), 13% (2/15), 88% (29/33) (14)    AMR and travel to Northern Africa:  - OR 3.32 (3.32-8.99) (14)  - Risks: 40% (4/10), 42% (34/81) (14)    AMR and travel to Central & Eastern Africa:  - Risks: 30% (17/56), 27.8% (57/205), 25.5% (12/47) (14)    AMR and travel to Western Africa:  - Risks: 18.9% (20/106), 49% (39/80) (14)    AMR and travel to Southern Africa:  - Risks: 12% (3/26), 6% (7/116), 11% (2/18) (14)    AMR and travel to Africa:  - OR 11.8 (2.78-50.4) (14), OR 14.8 (2-110.2), OR 7.7 (2.8-17.2) (8)  - Risks: 4% (1/25), 11% (2/18), 6.25% (1/16), 5.1% (10/195), 38.5% (5/13) (14)    AMR and travel to Latin America / Central & South America:  - OR 1.4 (1,13-1,74), OR 5.47 (2.09-24.33), OR 5.3 (2.1-13.5) (8,14)  - Risks: 25% (7/28), 6% (2/32),  27.9% (24/86), 18.3% (33/180), 5.56% (1/18), 50% (1/2), 7.6% (14/184), 8% (5/63), 7% (1/15), 5% (4/76) (14)  - Central & South America as region of provenance of AMR enteric bacteria (as % of a sample): 13.13% (n=3623) (4)  - Central & South America as region of provenance of resist ant Shigella spp. (as % of a sample): 17.92% (n=1242) (4)    AMR and travel to Southern Europe:  - Risks: 13% (2/16), 25% (2/8) (14)  - OR 15.2 (2.8-83.4) (8)    AMR and travel destination (not specified):  - PR 2.4 (1.4-4.2), PR 7.63 (1.4976 to 38.8224) (14), PR 1.33 (1.04-1.15) (8)  - OR 27.01 (2.38-1773.28), 5.7 (4.1-7.8) (8)    Sexually transmitted illnesses and travel:  - 16 of18 studies on N Gonorrhoea reported probable link with international travel (12)    AMR and pilgrimages:  - OR 2.5 (1.6-3.8), OR 3.2 (1.4-7.5) (8)    AMR and eating food with local population while travelling:  -  6.9 (1.2-39.6) (14)    AMR in medical travellers:  - OR: compared to recreational travellers OR 1.99 (p < 0.001) (4)  - prevalence: nearly all AMR A Baumannii and P Aeruginosa were in medical travellers (4)  - OR: travellers with a hospital stay while travelling vs not: OR 18.7 (2.9-115.8) (14)    AMR and travel and ESBL Enterobacteriaceae:  - any antimicrobials: OR 2.37 (1.69 - 3.33) (22)    Nosocomial MRSA regions of provenance (as % of a sample):  - 18.52% from Asia (n=15) (4)  - 27.16% from Europe (n=22) (4)  - 18.52% from North Africa and West Asia (n=15) (4) |
|  | **Farming and Animal Husbandry** | AMR and contact with animals, any:  - OR 5.28 (1.14-24.47), OR 11.2 (3.90-33.20) (14)  - OR: 4.50 (0.88-22.98) (21)  - Risks: 11.5% (11/99) (14)    AMR and contact with livestock:  - OR 7.6 (3-26), OR 30.98 (4.06-236.39), OR 3.26 (2.12-5.00), OR 2.04 (1.46-2.86), 6.31 (3.44 - 11.57) (14)    AMR and contact with swine/pigs:  - OR 288.79 (30.3- 2750.7), OR 1.28 (1.09-1.50), OR 0.77 (0.65-0.92), OR 15.2 (1.2-33.4), OR 17.8 (2.4-795.3), OR 2.4 (1.2-4.8), OR 1.04 (1.02-1.06), OR 12.5 (1.14-111.7), OR 326.7 (37.9-2514.0), OR 2.51 (1.26-4.98), OR 4.41 (2.62-7,41), OR 5.11 (2,16-12,09), OR 9.54 (4,77-19,06), OR 5.48 (1.65-18.3) (14),  OR 6.58 (3.5 - 12.38) (21), OR 20.455 (7.831-64.386),  5.48 (1.65-18.3) (8)  - PR 8.4 (5.6-12.6), PR 6.1 (3.8-10.0), PR 5.8 (3.9-8.4), PR 2.4 (1.0- 5.7), PR 2.5 (1.0- 6.4), PR 1.67 (1.32-2.11), PR 1.77 (1.36-2.30), PR 1.91 (1.42-2.57), PR 1.51 (1.24-1.84), PR 1.14 (1.07-1.20), PR 1.62 (1.22-2.17), PR 1.73 (1.25-2.38), PR 1.83 (1.27-2.63), PR 1.49 (1.16-1.90), PR 1.13 (1.05-1.22), PR 2.03 (1.21-3.41), PR 2.2 (1.23-3.93), PR 2.52 (1.31-4.84), PR 1.76 (1.12-2.74), PR 1.18 (1.04-1.35), PR 7.42 (3.71- 14.83), PR 3.38 (2.07-5.53), PR 16.3 (3.75-70.6) (14)  - Risks: 22%, 53%(42/79), 19.2%(10/52), 92.45%(49/53), 29.14%(7/24), 20%(5/25), 60%(3/5), 100%(7/7), 100% (2/2), 13% (7/52), 27% (7/26), 10.42% (5/48), 50% (24/48), 20% (12/48), 8.3% (1/12) (14)    AMR and contact with cattle:  - OR 6 (1,2-61,9), OR 5.4 (1,1-33,4), OR 6.9 (1,4-67,8), OR 0.7 (0.1-0.8), OR 2.16 (1.11-4.20), OR 3.56 (1,12-11,34) (14), OR 8.6 (1.7-42.9), 591 (5.1-69112.7) (8)    AMR and contact with poultry:  - OR 6 (2.302-15.640), OR 7.33 (1.804-29.816), OR 10.56 (2.539-43.918), OR 4.09 (1.020-16.403), OR 35 (5.617-218.106), OR 6.29 (1.598-24.727), OR 10.56 (4.154-43.918), OR 33.1 (3.47-316), OR 4.61 (1.23-17.2), OR 5.3 (2.2-12.7), OR 1.9 (1.01-3.60), OR 2.1 (1.13- 3.95), OR 2.97 (1.48-5.94) (14), OR: 4.94 (1.32-18.41) (21), OR 32 (3.21-1462), 2.97 (1.48-5.94) (8)  - PR 13.2 (1,8-98,2) (14)    AMR and contact with mink:  - OR 2.26 (1.29-3.98) (14)    AMR and contact with hog:  - PR 2.13 (1.14- 4.92), PR 2.64 (1.47- 4.75) (14)    AMR and contact with horses:  - Risks: 19.2% (67/349), 13.8% (4/29) (14)    AMR and contact with goats:  - Risks: 50% (1/2)  (14)    AMR and working as a veterinarian:  - with swine: OR 0.1 (0-0,5) (14)  - with small animals: OR 6.6 (14)  - Risks with dogs & cats: 16.7% (3/18)  - with any: 6 (1.23-58 ), 0.34 (0.14-0.66) (14)  - Risks, any: 1.4%, 7.5%, 9.5%, 2.3% (4/169) (14)    AMR and contact with pets (dogs/cats):  - OR 0.24 (0,06-0,09), 0.6 (0.4-0.9), 0.5 (0,4-0,8) (14)  - Risks of AMR if pets vs no pets at home: 2% (1/49) vs 4% (2/41) (14)  - OR 6.7 (1-42.6) (8)    AMR and working in agriculture, any:  - OR 13 (4.9-36) (14)    AMR and pigsty washing:  - OR 2.82 (1.160 - 6.873) (14)    AMR and working on farm:  - small vs big form: OR 0.2 (0.07-0.53) (14)  - Risks with exposure to small/medium farm: 9% (20/222) (14)    AMR and contact with manure:  - OR 3.73 (1.477-9.425) (14)    AMR and regularly visitor to farms  - Risk: 18.8% (14) |
|  | **Food Supply Chain** | AMR and handling raw meat regularly:  - OR 1.6 (1.2-2.0) (14)  - PR 1.32 (1.03-1.70), PR 1.1 (1.02-1.19)  (8,14)    AMR and being a foodhandler:  - Risks: 3.1% (70/2230), 20.37% (33/162), 1.5% (16/1091) (14)    AMR and slaughterhouse:  - OR 1.7 (1.44-1.9) (8)  - vs farm: OR 2.11 (1.07-4.19), PR 2.54 (1.16-5.56), Risks of transmission from slaughterhouse: 4% (4/134) (14)    AMR and food distribution:  - OR 3.47 (1.341-8.988) (14)    AMR and eating pork:  - OR 3.5 (1.8-6.6) (14)    AMR and eating dried poultry:  - OR 9 (1.8-45.2) (14)    AMR and raw milk drinking:  - 1.38 (7,89-13,66) (14)    AMR from food-producing animals consuming antibiotics:  - OR 1.09 (1.03-1.153) (8) |
|  | **Water Contamination** | AMR and drinking water from wild (wells) vs tap:  - OR 6.57 (1.41-30.7) (14)  - PR 1.26 (1.03-1.54) (8,14)  - Risks: 35% (233/657) (14)    AMR and recreational bathing in wild waters:  - OR 4.09 (1.02-16.4) (14)  - Risks (seawater vs non-seawater): 5% (2/42) vs 2% (1/58) (14)    AMR and water source sharing with livestock:  - OR 1.89 (1,10-2,78), OR 7.54 (2,41-23,45), OR 6.23 (2,05-19,02), OR 9.23 (3,19-26,75), OR 11.7 (2,74-50,01) (14) |
|  | **Climate** | Resistance Rates and tropical climate vs non-tropical climate:  - Carbapenem-resistance rate: 77.9% vs 47.3% (5)  - Carbapenem-resistant P aeruginosa: 43% vs 34% (5) |
| **Institution & Policies** | **Poor Antibiotic Quality** | Resistance rates and expired antibiotics:  - Resistance rates increase 2-6-fold with expired vs unexpired antibiotics (10) |
|  | **Healthcare Financing** | ASM and child having health insurance:  - OR 1.30 (1.05-1.61) (3,11,20),  OR 2.31 (1.38-4.02) (3)  - OR 0.36 (0.1-1) (3)    Out-of-pocket system:  - 10-point increase in percentage of out-of-pocket health expenditure associated with 3.2% (1.17-5.15) increase in AMR  (8) |
|  | **Healthcare Governance** | Inappropriate antibiotic sales with licensed pharmacist vs without:  -In China: 34% with, vs 60% without (11)     Inappropriate antibiotic prescriptions (for common cold) depending on health facility type:  - In China: county hospitals 47%, township hospitals 44%, village clinics 71% (11)    Prescribing antibiotics in private vs public sector primary care practitioners  - In China: aOR 9. (11) |
|  | **National Income** | SMA and lower national income level  - OR in HIC: 2.13 (1.65-2.61). OR in LMICs:2.68 (1.30-4.06) (20)  - OR of high income vs low-income: 0.95 (0.92-0.99) (20)    MRSA and Gross National Income per capita  - in Asia Pacific: GNI per capita positively associated with MRSA prevalence: b=0.022 (0.005-0.039) (16)  - However, inversely associated with resistant proportion of Staph Aureus b= -0.024 (-0.041 - -0.007) (16)  - S Aureus resistance rates in LMICs 50%, in UMICs 67%, in HICs 69%. (5)    Resistance rates per national income levels:  - Carbapenem resistance in A Baumannii rate in LMICs was 58%, in UMICs 65%, in HICs 26% (5)  - Carbapenem resistance rate in P Aeruginosa rate in LMIcs was 41%, in UMICs 42%, in HICs 14% (5)  - MDR Klebsiella in Sub-Saharan Africa neonatal units vs in HICs: 3-20-fold higher (15) |
